# Long‐Acting Growth Hormone Versus Daily Growth Hormone for Growth Hormone Deficiency Patients: A Network Meta‐Analysis of Clinical Trials

**DOI:** 10.1002/edm2.70301

**Published:** 2026-07-31

**Authors:** Amir Abadi, Usra Ghanem, Hamid Ghanem, Ahmed Jalal Sawafta, Ayah Abulehia, Mohammed Ataya, Rahma Nairat, Alhammam Mohammad Sammour, Hazem Ayesh

**Affiliations:** ^1^ Faculty of Medicine and Allied Medical Sciences An‐Najah National University Nablus Palestine; ^2^ Faculty of Medicine Al‐Quds University Jerusalem Palestine; ^3^ Deaconess Health System Evansville Indiana USA; ^4^ Indiana University School of Medicine Indianapolis Indiana USA

**Keywords:** growth hormone deficiency, hormone replacement therapy, long‐acting growth hormone, somatropin

## Abstract

**Introduction:**

Current guidelines recognize daily recombinant human growth hormone (rhGH) as standard care and long‐acting growth hormone (LAGH) as an alternative for paediatric growth hormone deficiency (CHD). In this network meta‐analysis (NMA), we updated the evidence to evaluate comparative efficacy and safety of multiple dosing nodes of LAGH versus daily somatropin.

**Methods:**

Major databases, including PubMed, Embase, Cochrane Central, Scopus, Web of Science, and http://ClinicalTrials.Gov, were searched through April 2026 for randomized controlled trials (RCTs)in children with CHD. Outcomes included height velocity (HV), height SDS, and adverse events. Direct and indirect evidence was synthesized using a frequentist random‐effects NMA (OSF: https://doi.org/10.17605/osf.io/nugpe).

**Results:**

Eighteen RCTs (*n* = 3137) were analysed. In the primary random‐effects model for HV, weekly YPEG‐rhGH 0.1 mg/kg/week showed a significantly higher velocity compared to daily Somatropin (SMD: 5.02), whereas Somatrogon 0.25/0.48/0.66 mg/kg/week and Somapacitan 0.04 mg/kg/week showed lower HV. No significant differences were observed across treatments for height SDS. For IGF‐1 SDS, Lonapegsomatropin 0.24 mg/kg/week significantly increased levels (SMD: 0.74 [0.36–1.12]), while Somatrogon and Somapacitan 0.04 mg/kg/week demonstrated reductions. Safety profiles showed that Somatrogon significantly increased the risk of localized injection site erythema (RR: 10.55) and pain. However, overall discontinuation rates did not differ significantly between LAGH formulations and daily therapy across the network.

**Conclusions:**

Once‐weekly LAGH provides an effective frontline alternative to daily acting growth hormone, without compromising overall linear growth or safety, while displaying no differences in overall treatment discontinuation. Selection should be based on the patients' clinical profiles, specifically considering higher localized reactions with Somatrogon. Clinicians should adhere to formulation‐specific sampling windows (2–5 days post dose for Lonapegsomatropin; 96 h for Somatrogon) to accurately monitor steady‐state IGF‐1 levels.

## Graphical abstract

This network meta‐analysis of 18 randomized controlled trials demonstrates that once‐weekly long‐acting growth hormone formulations offer comparable efficacy and safety to daily somatropin for paediatric growth hormone deficiency. While discontinuation rates remain similar, agent selection should consider specific local tolerability profiles, notably higher injection site reactions with Somatrogon.

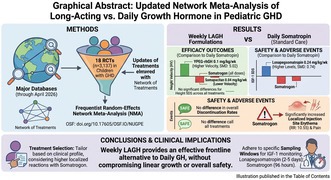

## 1 Introduction

Growth hormone deficiency (GHD) is a rare endocrine condition that can be subcategorized into childhood‐onset and adult‐onset GHD [1]. Prevalence of GHD was estimated to be 0.2–37/100,000 [1]. The management for GHD includes short‐acting and long‐acting growth hormone (LAGH) replacement therapies.

For many decades, the standard of care for children with GHD has been daily subcutaneous injections of recombinant human growth hormone (rhGH) or somatropin [2]. However, the 2024 International Consensus Statement now recognizes once‐weekly LAGH as a frontline alternative to daily rhGH for paediatric GHD [3]. Guidelines emphasize that while LAGH simplifies the treatment burden, clinical management must shift from daily monitoring to specialized protocols, for example, measuring IGF‐1 levels at specific ‘steady‐state’ intervals (typically 2–5 days post‐dose) to ensure levels remain within physiological range [3, 4].

While daily rhGH is highly effective in promoting linear growth and achieving near‐normal adult height, literature reports that this daily regimen carries a significant treatment burden (requiring ~365 injections per year), leading to a high non‐adherence rate (as high as ~82%), but recent studies claim a suboptimal adherence of 37% [5, 6]. In fact, studies have demonstrated that poor adherence can result in a loss of height velocity (HV) of approximately 1.1–1.3 cm/year compared to adherent patients, representing a substantial deviation from the predicted growth rate [7].

In recent years, the landscape of GHD treatment has been revolutionized by the approval and commercial availability of several distinct LAGH formulations, each designed for once‐weekly administration [8]. These include: Lonapegsomatropin, Somatrogon, Somapacitan, and various Pegylated (PEG‐LAGH) formulations. These drugs utilize different molecular mechanisms to prolong half‐life, creating a unique pharmacokinetic/pharmacodynamic profile compared to daily rhGH [4]. Prior meta‐analyses showed no significant difference in efficacy between long‐acting and short‐acting GH [9]. However, prior NMAs were conducted before the full Phase 3 data and regulatory approval of all three primary LAGH products.

An up‐to‐date review and network meta‐analysis are needed to pool the results of all recent pivotal trials, including those for Lonapegsomatropin (FDA/EMA approved), somatrogon (FDA/EMA approved), PEG‐rhGh and YPEG‐rhGH (not approved yet) and somapacitan (EMA approved), to provide a comprehensive comparison against the standard daily GH. A critical area of concern remains the long term safety of LAGH as highlighted in the 2024 International Consensus Statement on LAGH [3]. The safety of DGH is supported by decades of post‐marketing registries and long‐term follow‐up studies. Conversely, the long‐term impact of chronic exposure to the non‐physiological GH/IGF‐1 fluctuations resulting from LAGH is unclear. By conducting an updated network meta‐analysis of randomized controlled trials (RCTs), this review seeks to address this gap in the literature.

This network meta‐analysis aims to evaluate the efficacy and safety of LAGH in comparison to short‐acting growth hormone replacement therapies to treat GHD in children. We focused on key outcomes such as HV, height SDS, chronological age, height SDS bone age, change in height SDS, insulin‐like growth factor 1 (IGF‐1) SDS, and incidence of adverse events such as fasting glucose, haemoglobin A1c, and thyroid function. This analysis includes only RCTs and synthesizes direct and indirect evidence through a frequentist NMA framework.

Direct head‐to‐head trials comparing LAGH with daily rhGH therapies are limited, leading to uncertainty in clinical decision‐making. A network meta‐analysis enables the integration of both direct and indirect evidence across multiple interventions. This approach facilitates treatment ranking and provides clinicians with a clearer understanding of how LAGH therapy compares within the broader therapeutic landscape.

## 2 Methods

This systematic review and network meta‐analysis were done according to the PRISMA‐NMA guidelines (Supplementary S10). The protocol was registered on the Open Science Framework (OSF registration DOI: https://doi.org/10.17605/OSF.IO/NUGPE). The study design incorporated a framework using R to synthesize direct and indirect evidence from RCTs.

We included RCTs assessing therapies in children with GHD. Eligible interventions included LAGH (Lonapegsomatropin, Somatrogon, Somapacitan) compared with daily‐acting growth hormone (Somatropin) subcutaneous injection or placebo. Studies were required to report at least one of these outcomes: Height Velocity, HV Standard deviation Score, Height SDS (chronological age), Height SDS (bone age), Change in height SDS, Insulin‐like growth factor 1 (IGF‐1) SDS, Safety: Fasting glucose, Haemoglobin A1c (HbA1c), Thyroid function or adverse events. Studies focusing on other therapies or adults were excluded.

We systematically searched PubMed, Embase, Cochrane Central Register of Controlled Trials, Scopus, Web of Science and clinicaltrials.gov from database inception to April 2026. Search terms included a combination of LAGH, daily‐acting growth hormone, GHD, children and RCTs. The query strings used are available in Supplement S1. Only English articles were included, and any case reports, cohort studies, abstracts, and grey literature were excluded. Six reviewers independently screened titles and abstracts, followed by full‐text screening to identify eligible studies. Discrepancies were resolved by a third reviewer or by consensus.

Two independent reviewers extracted data using a standardized and piloted form. Collected variables included study characteristics (e.g., sample size, duration, region), patient demographics, intervention details, and outcome measures. The Cochrane Risk of Bias (ROB2) tool was used to assess study quality, with each domain scored as 1 (low), 2 (moderate), or 3 (high) risk [10]. We also evaluated overall confidence in network estimates using the CINeMA (Confidence in Network Meta‐Analysis) framework [11]. We conducted a frequentist random‐effects network meta‐analysis using the netmeta package in R. Risk ratios (RRs) were used for dichotomous outcomes, and mean differences (MDs) were used for continuous outcomes, each with 95% confidence intervals. We also used the Standard Mean Difference (SMD) when units of the outcomes were different. Somatropin was used as the reference comparator. When multiple doses were available for an intervention, all mentioned doses were used for primary analysis; sensitivity analyses included all doses. Treatment rankings were estimated using P‐scores derived from the netrank function.

Heterogeneity across studies was assessed using the I^2^ statistic, Tau^2^, and Cochran's *Q* test. Global inconsistency was evaluated via design‐by‐treatment interaction models using the decomp.design() function. We assessed transitivity by comparing baseline covariates (e.g., age, BMI, ALT levels) across treatment comparisons using ANOVA and boxplot visualizations. Sensitivity analyses included exclusion of high‐risk bias studies and leave‐one‐out analyses to evaluate the influence of individual trials on overall estimates.

## 3 Results

A total of 667 records were identified through database searching, then 90 full‐text articles were assessed for eligibility. A total of 18 RCTs with a total of 3137 patients were included [3, 8, 12, 13, 14, 15, 16, 17, 18, 19, 20, 21, 22, 23, 24, 25, 26, 27].

**FIGURE 1:**
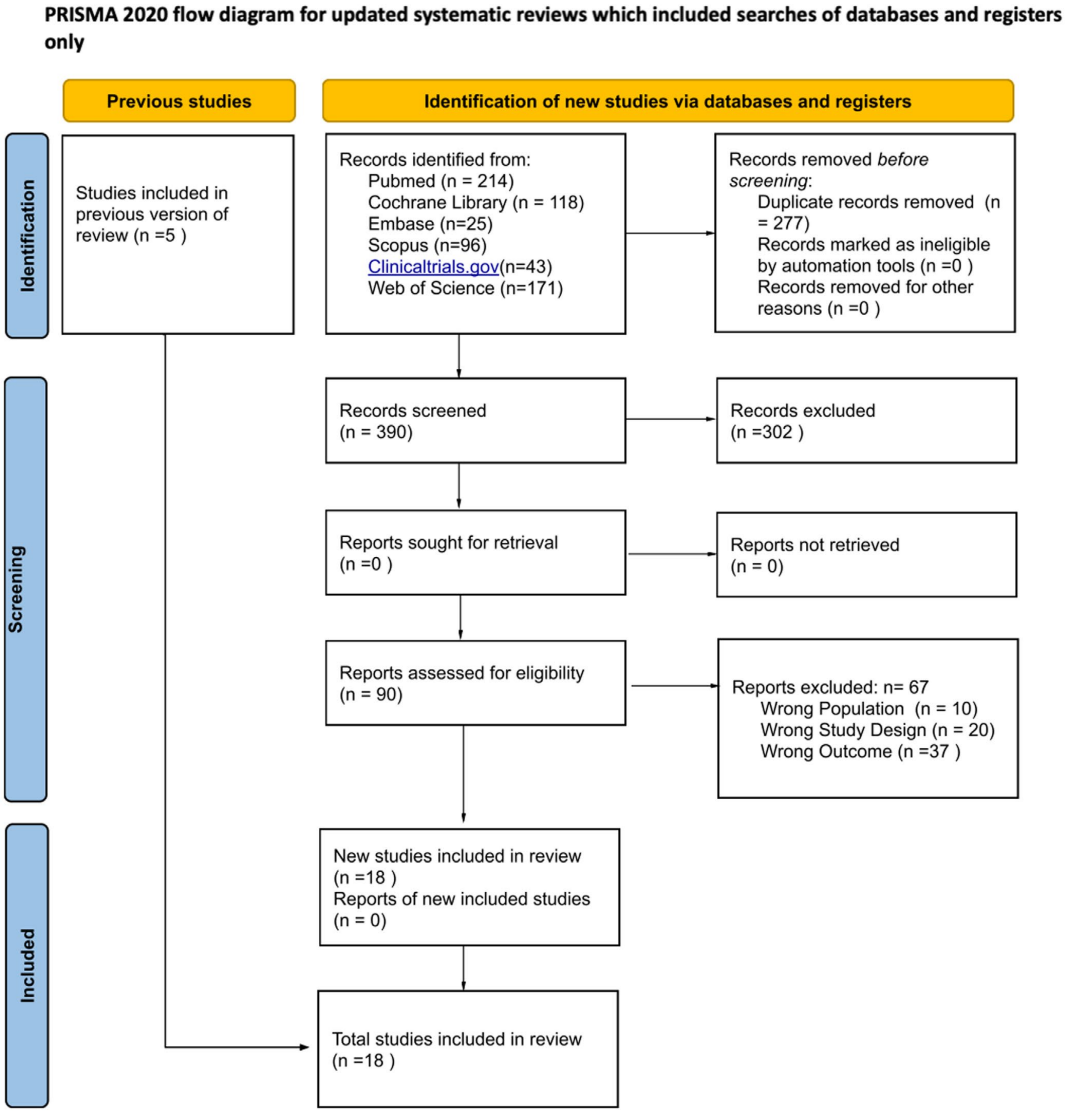
PRISMA flow diagram for this NMA.

These studies compared Lonapegsomatropin, PEG‐rhGH, Somapacitan, Somatrogon, YPEG‐rhGH, and Somatropin. Study durations ranged from 25 to 272 weeks. All studies enrolled patients with confirmed GHD. PRISMA flowchart can be seen in Figure 1. Study and patient characteristics can be viewed in Supplements S2 and S3. Risk of bias assessment is in Supplement S4.

### 3.1 Height Velocity

**FIGURE 2:**
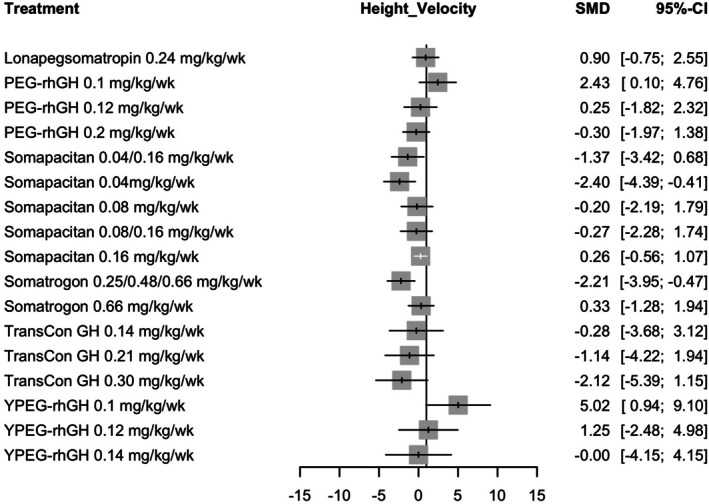
Forest plot of height velocity.

Various treatments from 18 studies were compared using a primary random effects model; YPEG‐rhGH 0.1 mg/kg/week showed a significantly higher velocity compared to daily rhGH 0.1 mg/kg/week (SMD: 5.02, 95% CI: 0.94–9.10, *p* = 0.015) (Figure 2). Nonetheless, two treatments showed significantly lower HV compared to daily rhGH: Somatrogon 0.25/0.48/0.66 mg/kg/mg (SMD: −2.21, *p* = 0.012) and Somapacitan 0.04 mg/kg/week (SMD: −2.40, *p* = 0.0183).

Among all interventions, Somapacitan 0.04 mg/kg/week ranked the highest based on P‐score (P‐score: 0.88) followed by Somatrogon 0.25/0.48/0.66 mg/kg/week (P‐score: 0.86) and YPEG‐rhGH 0.1 mg/kg/week (P‐score: 0.02) ranked the lowest. There is high heterogeneity in this network (*I* ^2^ = 68.5%, Tau^2^ = 0.59). Based on the CINeMA framework, the certainty of HV effectiveness in LAGH agents vs. Daily rhGH was very low due to high risk of bias according to ROB2. When excluding studies with a high risk of bias, heterogeneity became lower (*I* ^2^ = 58.6%) and several studies showed statistically significant differences compared to Somatropin, including Somatrogon (*p* = 0.00) and YPEG‐rhGH 0.1 mg/kg/week (0.01). In a common fixed effects model, Lonapegsomatropin 0.24 mg/kg/week became statistically significant (SMD: 0.90, 95% CI: 0.25–1.55, *p* = 0.006). The results were not consistently robust across all sensitivity analyses; significant differences emerged in multiple subsets. These variations across models indicate that while LAGH agents generally aim for equivalent clinical efficacy, specific formulations and doses yield statistically significant gains or losses in HV compared to daily Somatropin.

Finally, there is no evidence of publication bias detected using Egger's test (test statistic 0.76; *p*‐value of 0.45). The univariable meta‐regression indicated that the baseline size (*p* = 0.02) and baseline HV (*p* = 0.02) significantly influenced treatment effects. Other baseline covariates, including chronological age, bone age, BMI, weight, height SDS, treatment duration, study phase and growth hormone type did not show statistically significant influence and were balanced across treatment comparisons. These exploratory findings suggest that participants' initial growth parameters were important impacting the relative efficacy results observed within the network.

### 3.2 Height SDS

**FIGURE 3:**
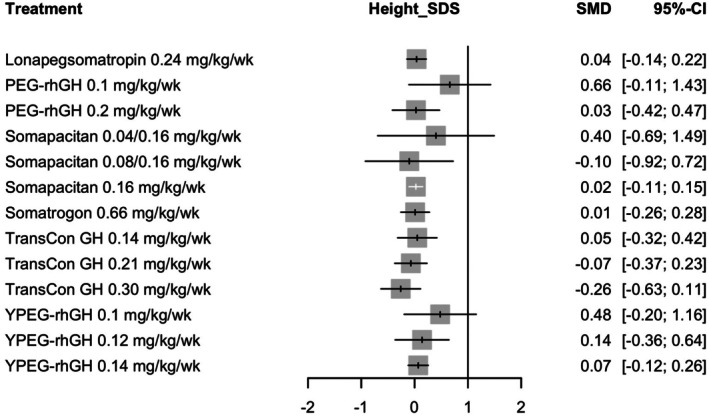
Forest plot of height SDS.

Under the random effects model, no treatment showed a statistically significant difference compared to daily rhGH. For instance, Lonapegsomatropin 0.24 mg/kg/week showed a SMDof 0.03 [95% CI: −0.14;0.21] (Figure 3). Among all interventions, TransCon GH 0.30 mg/kg/week ranked highest (P‐score: 0.87), followed by TransCon GH 0.21 mg/kg/week (P‐score: 0.69), while PEG‐rhGH 0.1 mg/kg/week (P‐score: 0.11) ranked the lowest. The network demonstrated low heterogeneity with an *I* ^2^ = 31.2% and Tau^2^ = 0.0087. Based on the CINeMA framework, 52 comparisons were rated as moderate confidence, 38 as low and 1 as very low. Univariate meta‐regression indicated that baseline covariates including chronological age and bone age were balanced and did not significantly influence effect sizes.

Sensitivity analyses revealed that the results were not entirely robust; excluding one specific trial in the leave‐one‐out analysis resulted in Lonapegsomatropin 0.24 mg/kg/week becoming statistically significant (SMD: 0.14, *p* = 0.01). Egger's test showed no evidence of publication bias (*p* = 0.07).

### 3.3 Insulin‐Like Growth Factor‐1 (IGF‐1) SDS

**FIGURE 4:**
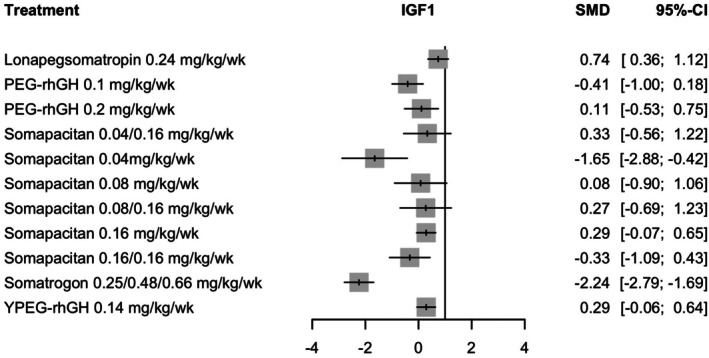
Forest plot of IGF‐1 SDS.

The network for IGF‐1 SDS incorporated 12 treatment regimens. In the primary random‐effects model, Lonapegsomatropin 0.24 mg/kg/week resulted in a statistically significant increase in IGF1 levels compared to Daily rhGH (SMD: 0.74, 95% CI: 0.36 to 1.12, *p* = 0.0002) (Figure 4). Conversely, two treatments showed statistically significant reductions compared to Daily rhGH: Somatrogon 0.25/0.48/0.66 mg/kg/mg (SMD: −2.24, 95% CI: −2.79 to −1.69, *p* = 0.00) and Somapacitan 0.04 mg/kg/week (SMD: −1.65, 95% CI: −2.88 to −0.42, *p* = 0.008). Among all interventions, Somatrogon ranked highest (P‐score: 0.98) while Lonapegsomatropin 0.24 mg/kg/week ranked lowest (P‐score: 0.05).

Heterogeneity for this outcome was low (*I* ^2^ = 14.1%, and Tau^2^ = 0.02) and tests for global inconsistency were non‐significant (*Q* = 3.49, *p* = 0.32). Based on the CINeMA framework, the certainty of evidence was rated as moderate for 24 comparisons, low for 33, and very low for 9. Transitivity checks via univariable meta‐regression indicated that baseline covariates, including chronological age, bone age, and baseline HV, were balanced across treatment comparisons and did not significantly influence the effect sizes.

Sensitivity analyses confirmed the robustness of these findings. When excluding studies with a high risk of bias (*k* = 8), results remained stable, with Lonapegsomatropin (*p* < 0.0001) and Somatrogon (*p* < 0.0001) maintaining statistical significance. Analysis excluding small sample size (*k* = 6) and leave‐one‐out approach also yielded consistent estimates, reinforcing the stability of the network. Finally, Egger's test detected no evidence of publication bias (test statistic: −0.78, *p* = 0.44).

### 3.4 Safety Outcomes

The treatment arms across the safety networks ranged from 4 to 11 providing a comprehensive view of the therapeutic safety landscape for GH treatment. For Fever, there was no significant difference between treatments. In terms of fasting plasma glucose (FPG), Lonapegsomatropin 0.24 mg/kg/week demonstrated a statistically significant reduction compared to daily rhGH (SMD: −3.10, 95% CI: −5.96 to −0.23, *p* = 0.03). It was also the highest ranked treatment for FPG with a P‐score of 0.97. For HbA1c, no significant differences were observed between any long‐acting GH treatment and daily rhGH. Nonetheless, there was no statistical difference between LAGH formulations and daily rhGH for causing headaches, and risk estimates for hypothyroidism were also non‐significant.

As for respiratory side effects, there were no LAGH formulations that showed a statistical difference in risk compared to daily rhGH for cough, influenza, and upper respiratory tract infections.

Regarding injection site erythema, Somatrogon 0.66 mg/kg/week was associated with a significantly higher risk of erythema compared to daily rhGH (RR: 10.55, 95% CI: 1.37–81.04, *p* = 0.0002). Somapacitan 0.16% mg/kg/week ranked as the most tolerable for this outcome (P‐score: 0.86). For injection site pain, Somatrogon 0.25 and 0.48 mg/kg/week doses showed a significantly higher risk of pain (RR: 5.33, 95% CI: 1.58–17.92, *p* = 0.004). TransCon GH 0.21 mg/kg/week ranked as the best‐tolerated treatment (P‐score: 0.72). Heterogeneity for injection pain was low (*I* ^2^ = 13%, and Tau^2^ = 0.02) Sensitivity, CINeMA framework, meta‐regression and publication bias for these outcomes can be in Supplements S5–S9.

### 3.5 Treatment Discontinuation

**FIGURE 5:**
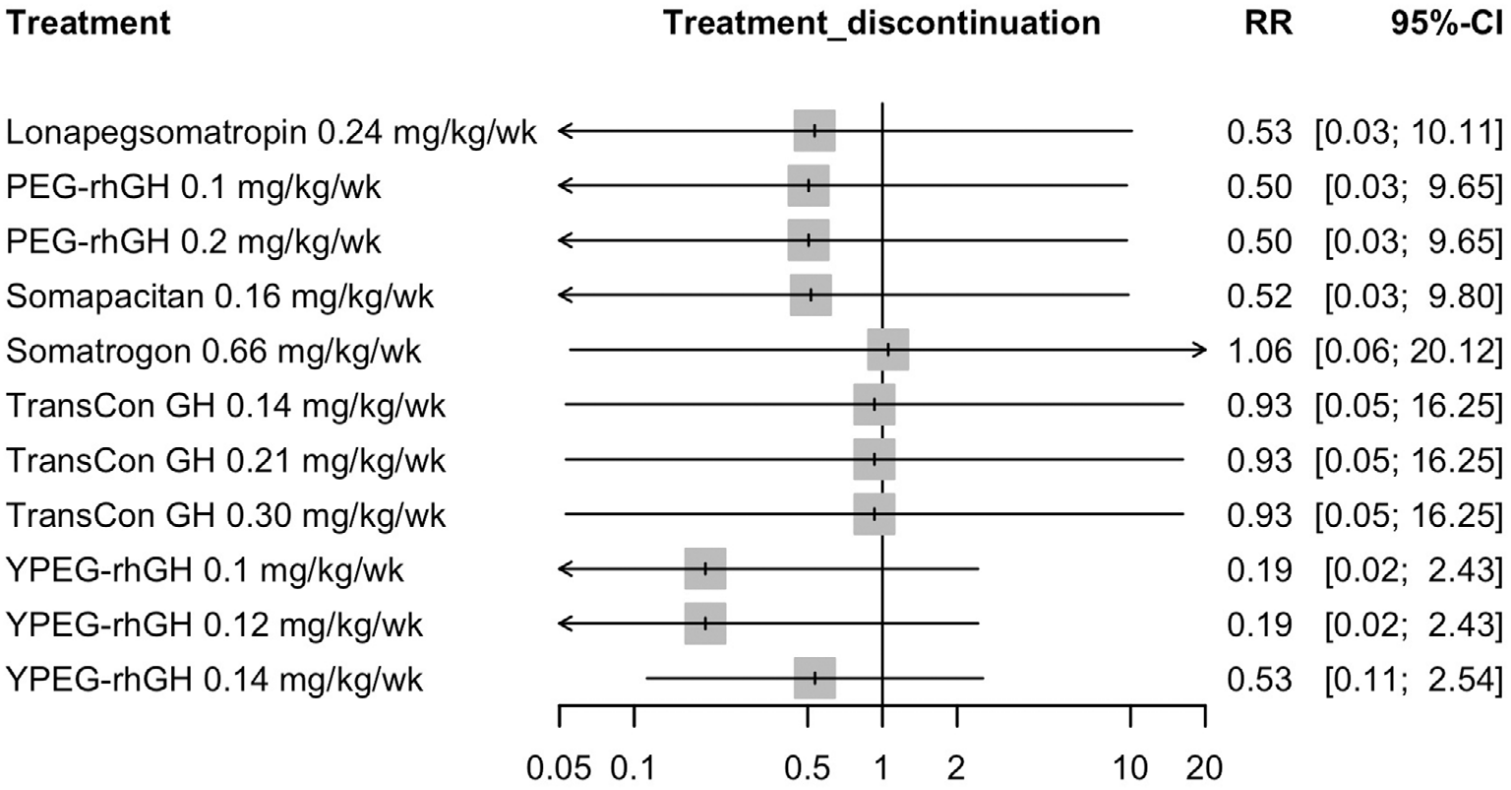
Forest plot of treatment discontinuation.

This network meta‐analysis compared various LAGH therapies to Daily rhGH across 12 studies. There was no statistically significant difference in discontinuation rates (Figure 5). YPEG‐rhGH 0.1 and 0.12 mg/kg/week ranked the highest (P‐score: 0.73) followed by PEG‐rhGH 0.1 and 0.2 mg/kg/week (P‐score: 0.52), while Somatrogon 0.66 mg/kg/week (P‐score: 0.37) and Somatropin (P‐score: 0.33) ranked the lowest. The analysis detected low heterogeneity (*I* ^2^ = 20.9% and Tau^2^ = 0.28). According to the CINeMA framework, the certainty of evidence was quite limited, with 63 comparisons rated as very low confidence and 3 rated as low confidence, due to low precision, limiting the confidentiality of this evidence. Univariable meta‐regression was conducted to explore the impact of several baseline covariates (including age, size, weight, BMI, chronological age, bone age, HV, height SDS, Duration, and trial phase). There were no significant covariate imbalances found to influence treatment discontinuation, supporting transitivity assumption.

Sensitivity analyses further confirmed the robustness of these primary findings. When studies with a high‐risk of bias were excluded, the estimates for treatments like Lonapegsomatropin 0.24 mg/kg/week (RR 0.53) and PEG‐rhGH (RR 0.5044) remained identical to the primary analysis. Excluding small sample sizes reduced the network to 5 studies and while effect sizes shifted for Somatrogon (RR: 2.1101 95% CI: 0.19–22.93), the findings remained statistically not significant (*p* = 0.96). Leave‐One‐Out analysis was largely robust, with *I* ^2^ remaining 0.0%. Results for common and random effects models were identical due to a lack of heterogeneity. The consistency for non‐significant findings across all sensitivity analyses increases confidence that there are no major discontinuation rates between these growth hormone treatments. Finally, Egger's test (12 studies) indicated that there is no evidence of publication bias (*p* = 0.81), with a low concern level.

## 4 Discussion

This network meta‐analysis (NMA) of 18 RCTs involving 3137 patients demonstrates that LAGH therapies, including Lonapegsomatropin, PEG‐rhGH, Somapacitan, Somatrogon, and YPEG‐rhGH, provided comparable efficacy to daily Somatropin in patients with GHD.

In terms of HV, weekly YPEG‐rhGH 0.1 mg/kg/week had a significantly higher HV compared to Daily rhGH 0.1 mg/kg/week (SMD: 5.02, 95% CI: 0.94–9.10, *p* = 0.01). Yet, two treatments showed a significantly lower HV compared to daily rhGH; Somatrogon o.25/0.48/0.66 mg/kg/week and Somapacitan 0.04 mg/kg/week. However, on network rankings, Somapacitan 0.04 mg/kg/week ranked the highest (P‐score: 0.88) while YPEG‐rhGH 0.1 mg/kg/week ranked the lowest (P‐score: 0.02). The sensitivity analyses highlighted this discrepancy. When excluding studies with a high risk of bias, network heterogeneity became lower (*I* ^2^ = 58.6%) and several studies showed statistically significant differences compared to Somatropin, including Somatrogon (*p* = 0.00) and YPEG‐rhGH 0.1 mg/kg/week (*p* = 0.01). Furthermore, switching to a common fixed‐effects model shifted the significance profile, rendering Lonapegsomatropin 0.24 mg/kg/week statistically significant (SMD: 0.90, 95% CI: 0.25–1.55). These variations across models indicate that while LAGH agents generally aim for equivalent clinical efficacy, specific formulations doses demonstrated statistical significance compared to daily Somatropin depending on the analytical model applied.

The significantly higher HV observed with YPEG‐rhGH 0.1 mg/kg/week (SMD: 5.02, [0.94–9.10]) compared to daily rhGH represents the most striking finding in the HV network and warrants careful interpretation. YPEG‐rhGH (pegpesen) is a novel Y‐shape branched PEGylated rhGH with a mean half‐life of 65 to 120 h (~20 times longer than that of daily rhGH) [17]. The Phase 3 clinical trial by Liang et al. was conducted across 23 centers in China over 52 weeks, enrolling 391 paediatric patients randomized 2:1 to weekly pegpesen or daily rhGH. However, the primary endpoint of that same trial showed a growth velocity difference of only −0.27 cm/year [−0.48, 0.23], confirming non‐inferiority but providing no evidence of superiority over daily rhGH [17].

The large SMD of 5.02 observed in our NMA model therefore reflects the influence of dose heterogeneity across network nodes rather than a true superior biological effect, as the earlier Phase 2 pharmacokinetic study by Liang et al. used the lower 0.1 mg/kg/week dose in a 12 weeks trial of only 43 patients [18]. The extremely wide confidence interval (0.94–9.10) further reflects this statistical uncertainty. The inconsistency between this large SMD and YPEG‐rhGH's lowest P‐score ranking in the network (P‐score: 0.02) further supports that estimates and network rankings can diverge substantially under high heterogeneity when comparator dosing differs across studies. Furthermore, YPEG‐rhGH remains unapproved by any western regulatory authority, limiting its immediate clinical applicability. This finding should therefore be regarded as a hypothesis rather than confirmatory and dedicated head‐to‐head trials with standardized dosing are needed before YPEG‐rhGH can be positioned confidently relative to approved LAGH formulations.

In contrast to HV, the network for Height SDS showed lower heterogeneity with no statistically significant difference compared to daily rhGH. For instance, Lonapegsomatropin 0.24 mg/kg/week showed an SMD of 0.03 [−0.14, 0.21]. Among these interventions, TransCon GH 0.30 mg/kg/week ranked the highest (P‐score: 0.87), while PEG‐rhGH 0.1 mg/kg/week ranked the lowest (P‐score: 0.11).

A major strength of this study was the use of univariate meta‐regression to evaluate transitivity and identify modifiers of treatment effect. The baseline size (*p* = 0.02) and baseline HV (0.02) significantly influenced the observed treatment effects. Other variables did not show statistically significant influence and were balanced across treatment comparisons. These findings suggest that participants' initial growth parameters were highly important in impacting the relative efficacy results observed within the network, signalling a potential critical catch‐up or floor/ceiling effect. Children presenting with more severe baseline growth restriction may exhibit a more pronounced relative response to specific formulations.

The network of IGF‐1 SDS revealed a low heterogeneity (*I* ^2^ = 14.1%) and non‐significant global inconsistency (*Q* = 3.49). In the primary random‐effects model, Lonapegsomatropin 0.24 mg/kg/week resulted in a statistically significant increase in IGF‐1 levels compared to daily rhGH (SMD: 0.74). Analysis excluding small sample sizes and the leave‐one‐out approach also yielded consistent estimates.

The IGF‐1 SDS profiles across LAGH agents represent one of the most clinically important mechanistically informative findings of this NMA. Lonapegsomatropin 0.24 mg/kg/week produced a significant increase in IGF‐1 SDS (SMD: 0.74) compared to daily rhGH. This is mechanistically consistent with its prodrug design. Lonapegsomatropin release fully active unmodified Somatropin via autocleavage of the TransCon Linker, exhibiting tissue distribution and GH receptor affinity identical to that of endogenous GH, while its ~25 h half‐life sustains hepatic IGF‐1 stimulation throughout the weekly dosing interval [8]. Consistently, the enliGHten 6‐year trial confirmed that mean weekly average IGF‐1 SDS remained within the 0–2 SDS target range through treatment with Lonapegsomatropin and that growth was maintained during pubertal development without dose escalation [28]. In contrast, Somatrogon 0.25/0.48/0.66 mg/kg/week showed a statistically significant reduction in IGF‐1 SDS in our NMA (SMD: −2.24). This apparent paradox is explained by the time‐dependent pharmacokinetics of Somatrogon's fusion architecture: IGF‐1 peaks at ~48 h post‐dose and then declines, such that measurements not taken at the protocol specific 96‐h post‐dose window will systematically underestimate mean IGF‐1 exposure [29].

This pharmacokinetic feature has direct clinical implications. A recent 2025 study by Nayak et al. developed a population PK/PD model confirming that only samples drawn at exactly 96 h post dose represent the mean IGF‐1 SDS across the weekly Somatrogon dosing interval. Same reference Additionally, real‐data show that dose reductions due to transiently elevated IGF‐SDS occur in approximately 55.5% of Somatrogon treated patients, particularly those with severe GHD or in advanced puberty [30]. This means that the lower aggregate IGF‐1 SDS observed in our NMA partly reflects protocol‐driven dose titration across trials rather than a genuinely suppressed IGF‐1 response. The Albers et al. NMA similarly found that the change from baseline in average IGF‐1SDS was significantly higher for Somatrogon versus all comparators, confirming that the timing of measurements and dose adjustments substantially influence network‐level IGF‐1 estimates [9]. Taken together, clinicians must apply formulation‐specific sampling windows (~96 h post‐dose for somatrogon, and 2–5 days post‐dose for lonapegsomatropin) to obtain clinically meaningful IGF‐1 measurements that accurately reflect mean exposure, as mandated by the 2024 International Consensus Statement [3].

From a safety perspective, FPG, HbA1c, headaches, hypothyroidism and systemic side effects were analysed. In terms of FPG, Lonapegsomatropin 0.24 mg/kg/week demonstrated a statistically significant reduction compared to daily rhGH (SMD: −3.10) and was the highest ranked treatment for FPG. For HbA1C, no significant differences were observed. Likewise, there was no statistical difference between LAGH formulations and daily rhGH for causing headaches, and risk estimates for hypothyroidism were also non‐significant. Systemic respiratory side effects including cough, influenza, and upper respiratory infections showed no statistical difference in risk between any LAGH formulation and daily rhGH.

However, Somatrogon 0.66 mg/kg/week was associated with a significantly higher risk of erythema compared to daily rhGH (RR: 10.55), while Somapacitan 0.16 mg/kg/week ranked as the most tolerable for this outcome (P‐score: 0.86). For injection site pain, Somatrogon 0.25 and 0.48 mg/kg/week doses showed a significantly higher risk of pain (RR: 5.33, *p* = 0.004). TransCon GH 0.21 mg/kg/week ranked as the best tolerated treatment for pain (P‐score: 0.72), with a low heterogeneity network (*I* ^2^ = 13%, Tau^2^ = 0.02).

Despite these striking differences in local injection site pain and localized erythema, this network meta‐analysis revealed a critical clinical finding: across 12 studies, there was no statistically significant difference in treatment discontinuation rates between various LAGH agents and daily rhGH.

The finding that most LAGH therapies showed no statistically significant difference to daily rhGH in terms of HV and Height SDS suggests that the clinical burden of daily injections can be reduced without compromising linear growth [21]. However, the high risk of injection site erythema and pain associated with Somatrogon (RR10.55) is a critical clinical consideration. While LAGH offers convenience, clinicians must weigh the benefit of reduced injection frequency against the potential of localized adverse reactions.

The clinical management of paediatric GHD is currently undergoing a paradigm shift to include once‐weekly LAGH. In contemporary practice, clinicians must navigate a landscape featuring three primary FDA‐approved agents: lonapegsomatropin (a prodrug), somatrogon (a fusion protein), and somapacitan (albumin‐binding analogue). While this network meta‐analysis showed no statistically significant difference of LAGH agents to daily somatropin in terms of HV and SDS, the choice of agent should be adjusted to the individual patient's clinical profile [3]. A central advantage of LAGH is its potential to overcome the well‐documented barriers to adherence associated with daily injections. While earlier reviews reported a high non‐adherence rate (82%) in long‐term daily therapy [5], a recent prospective study reports a 37% suboptimal adherence, which correlated with significantly lower mean IGF‐1 SDS levels (−1.1 vs. −0.2) and poorer metabolic profiles compared to the optimal adherence group [31]. Despite these real‐world challenges, our study found no statistically significant difference in treatment discontinuation rates between daily and once‐weekly LAGH within the controlled environment of RCTs. This suggests that while LAGH is designed to improve persistence, the structural nature of clinical trials may mask adherence benefits typically observed in clinical practice. However, practical application requires a nuanced approach by monitoring IGF‐1 levels at the steady‐state (2–5 days post dose) and specifically counsel patients on Somatrogon regarding its higher risk of injection site erythema and pain [6, 8]. Our findings align with recent large‐scale RCTS and smaller pairwise meta‐analyses [7, 9]. However, this NMA provides a more comprehensive landscape by integrating direct and indirect evidence from all 5 different Long‐acting formulations. While previous clinical Trials such as REAL and enliGHT often focused on a single LAGH, our NMA highlights that no single agent provides a statistically superior LAGH over daily Somatropin in terms of efficacy and safety [6, 8].

A clinically important limitation of this and all prior NMAs in this field is the inability to perform subgroup analyses by pubertal status, GHD aetiology, or baseline IGF‐1 levels, factors that may substantially influence treatment response. All pivotal trials included in this NMA enrolled exclusively prepubertal children, consistent with the eligibility criteria of the phase 3 Somatrogon, Lonapegsomatropin, and Somapacitan trials. While this enrollment homogeneity supports internal validity, it limits extrapolation to pubertal patients. The enliGHten long‐term extension provides the most relevant data in this regard: growth was maintained throughout pubertal development with lonapegsomatropin, and the dose remained stable without escalation across pubertal stages [28]. However, comparable pubertal efficacy data across all five formulations in a unified NMA framework are not yet available. In GHD aetiology, childhood GHD encompasses congenital, acquired, and idiopathic forms, with organic and severe etiologies associated with more profound IGF‐1 deficits [13]. Children with organic GHD may exhibit a more pronounced initial response to GH replacement due to deeper pre‐treatment IGF‐1 suppression, whereas those with idiopathic GHD may show greater variability in response. Our univariate meta‐regression identified baseline HV (*p* = 0.02) and baseline sample size (*p* = 0.02) as significant moderators of treatment effect across the HV network, consistent with a catch‐up growth mechanism that is expected to be more pronounced in children with greater severity of growth restriction, a characteristic that overlaps with, but is not identical to, GHD aetiology. However, aetiology‐level data were not available in an extractable form from the included trials, precluding formal subgroup analysis. Similarly, baseline IGF‐1 SDS varied across included studies but was unavailable at the individual patient level for meta‐regression. Addressing these subgroup questions definitively requires individual patient data (IPD) meta‐analysis, which we identify as the highest methodological priority for future synthesis in this field.

This study has several strengths. We conducted a comprehensive literature search across major databases with a total of 3137 patients, providing an adequate sample size across 18 RCTs for efficacy and safety outcomes. The use of a frequentist network meta‐analysis allowed us to simultaneously compare multiple interventions and rank them based on efficacy. We applied robust methodological tools, including the CINeMA framework, to evaluate the certainty of evidence and a structured transitivity assessment using baseline covariate balance. Our sensitivity analyses, including exclusion of high‐risk studies and leave‐one‐out tests, confirmed the stability of our conclusions. To our knowledge, this the first NMA to simultaneously compare all five distinct LAGH formulations (Lonapegsomatropin, Somatrogon, Somapacitan, PEG‐rhGH and YPEG‐rhGH) using a dose‐disaggregated approach across both efficacy and safety outcomes, while aligning findings with the 2024 International consensus on LAGH [3]. Prior pair‐wise meta‐analysis and smaller NMAs either predated the full Phase 3 approval datasets for all against or were restricted to two or three formulations limiting their ability to generate comparative treatment rankings across the full current therapeutic landscape [7, 9].

Despite these strengths, our analysis has limitations that must be considered when interpreting the findings. First, heterogeneity in HV network remained high (*I* ^2^ = 68.5%), and while univariate meta‐regression identified baseline sample size and baseline HV as significant moderators, these are exploratory findings from aggregate data and do not fully explain the observed variance. Differences in trial populations, GHD severity and dosing protocols across studies likely contribute to this heterogeneity and limit the precision of cross‐formulation comparisons. Second, the certainty of evidence, as assessed by the CINeMA framework, was rated low to very low for the majority of comparisons, high risk of bias in several trials per ROB2 and reliance on indirect evidence. Clinicians should therefore treat the P‐score rankings reported here as exploratory signals rather than definitive guidance. Third, this NMA was not designed with a predefined non‐inferiority margin; consequently, the conclusion that most LAGH formulations showed no statistically significant difference compared to daily rhGH should not be interpreted as formal non‐inferiority. Future trials should prospectively define a clinically meaningful threshold to enable rigorous non‐inferiority conclusions. Fourth, sub‐group analyses by clinically relevant factors (Pubertal status, GHD aetiology and baseline IGF‐1 levels) were not feasible from the available published data, leaving an open question of which patient subgroups may respond differently to specific formulations. Fifth, although all approved doses were included as separate network nodes in the primary analysis, trial durations ranged from 25 to 272 weeks and shorter trials may not capture long‐term growth trajectories or delayed safety signals, especially given the non‐physiological GH/IGF‐1 fluctuation profiles characteristic of long‐acting agents. Finally, the long‐term safety of LAGH remains insufficiently characterized; while this NMA found no significant differences in systemic adverse events, the evidence base for chronic outcomes (glucose metabolism, thyroid function, and IGF‐1) relies on post‐marketing registries and extended follow‐up studies that were outside the scope of this RCT‐based analysis.

While our network meta‐analysis confirms that LAGH is a viable alternative to daily Somatropin, several gaps remain. Future research should focus on head‐to‐head RCTs between LAGH to move beyond indirect comparisons. Finally, investigating the cause of high heterogeneity in HV through individual patient data meta‐analysis could help identify which subgroups benefit the most.

## 5 Conclusion

This network meta‐analysis of 18 RCTs (*n* = 3137) found that most LAGH formulations demonstrated no statistically significant differences in HV or height SDS compared to daily rhGH. Lonapegsomatropin was associated with a significant increase in IGF‐1 SDS levels and a favourable fasting plasma glucose profile. Safety profiles were broadly comparable. However, Somatrogon was associated with a significantly higher risk of injection site erythema and pain relative to daily rhGH, which represents an important clinical consideration in agent selection. Treatment discontinuation rates did not differ significantly across groups. These findings support once‐weekly LAGH as a clinically viable alternative to daily somatropin in paediatric GHD, with agent selection guided by individual patient profile and local tolerability. Future head‐to‐head trials with predefined non‐inferiority margins and individual patient data are needed to confirm these findings and identify subgroups that benefit most.

## Author Contributions

**Usra Ghanem:** investigation, conceptualization, writing – review and editing, writing – original draft, methodology. **Amir Abadi:** methodology, conceptualization, investigation, writing – original draft, writing – review and editing, visualization, data curation. **Mohammed Ataya:** investigation, methodology, writing – review and editing. **Hamid Ghanem:** methodology, investigation, writing – review and editing. **Ayah Abulehia:** methodology, investigation, writing – review and editing. **Rahma Nairat:** writing – review and editing, methodology, investigation. **Hazem Ayesh:** software, formal analysis, project administration, supervision, resources, writing – review and editing, validation. **Alhammam Mohammad Sammour:** methodology, investigation, writing – review and editing. **Ahmed Jalal Sawafta:** investigation, methodology, writing – original draft, writing – review and editing, data curation, resources.

## Funding

The authors have nothing to report.

## Conflicts of Interest

The authors declare no conflicts of interest.

## Supporting information


**Data S1:** edm270301‐sup‐0001‐DataS1.docx.

## Data Availability

Data available on request from the authors.
