# Supplementary material for: Long‐Acting Growth Hormone Versus Daily Growth Hormone for Growth Hormone Deficiency Patients: A Network Meta‐Analysis of Clinical Trials
Source: Endocrinol Diabetes Metab. 2026 Jul 31;9(5):e70301. doi: 10.1002/edm2.70301 (PMC13428039; doi:10.1002/edm2.70301)
Supplement: Supplementary file 1 — Data S1: edm270301‐sup‐0001‐DataS1.docx. [file EDM2-9-e70301-s001.docx]

**Supplementary Material**

**Content Table**

[**Supplement 1: Search Strategy ……………………………………………………………………………………………. 3**](#lfma04lw3u5)

[**Supplement 2: Study Characteristics Table ……………………………………………………………………………... 4**](#kxe6t7r2kj49)

[**Supplement 3: Patient Characteristics Table …………………………………………………………………………….18**](#2pujbe72h6b6)

[**Supplement 4: Risk of Bias Table for each outcome ………………………………………………………………….. 27**](#mcpm48eetbjj)

[**Supplement 5: Publication bias -funnel plot- for each outcome ……………………………………………………. 28**](#wt534z4u1fzv)

[S5.1 Publication bias -funnel plot- for Height Velocity](#5fscpvexq0aa)

[S5.2 Publication bias -funnel plot- for Height SDS](#hsimr5k8guf1)

[S5.3 Publication bias -funnel plot- for treatment discontinuation](#1gssyhexa35x)

[S5.4 Publication bias -funnel plot- for Injection site erythema](#ut31724kwqfi)

[S5.5 Publication bias -funnel plot- for Influenza](#ljkui2bsgayp)

[S5.6 Publication bias -funnel plot- for Headache](#trj0gtaocrj6)

[S5.7 Publication bias -funnel plot- for Fever](#73lebuyshuyo)

[S5.8 Publication bias -funnel plot- for hypothyroidism](#539jdab6seds)

[S5.9 Publication bias -funnel plot- for Injection site pain](#ux6k62uzfb2e)

[S5.10 Publication bias -funnel plot- for IGF-1](#nesziqcexz96)

[S5.11 Publication bias -funnel plot- for HbA1C%](#bnktsmj5xscs)

[S5.12 Publication bias -funnel plot- for cough](#yxhcra5ddk65)

[S5.13 Publication bias -funnel plot- for URTI](#8ih3weakcg8t)

[S5.14 Publication bias -funnel plot for FPG](#gcaayhjdmmuy)

[**Supplement 6: League table for each outcome ………………………………………………………………………..34**](#g6ekd07b5et2)

[S6.1 League table for Height Velocity](#kbclknm67f84)

[S6.2 League table for Height SDS](#ovz4t4eahuh1)

[S6.3 League table for Treatment discontinuation](#otdcxvsy9b2y)

[S6.4 League table for Injection site erythema](#efonpgot4ym9)

[S6.5 League table for Influenza](#e1rpj75yg0u5)

[S6.6 League table for Headache](#zflf0gj10ehb)

[S6.7 League table for Fever](#nxjsfrrabe5x)

[S6.8 League table for hypothyroidism](#s7fx8i72qn15)

[S6.9 League table for Injection site pain](#ewih1b6zcavo)

[S6.10 League table for IGF-1](#psmqo1mmo39u)

[S6.11 League table for HbA1C%](#rugoqa5xmqou)

[S6.12 League table for cough](#vk7e0yza5cai)

[S6.13 League table for URTI](#am5zb8jhim0m)

[S6.14 league table for FPG](#jc0uzsw5g7gs)

[**Supplement 7: Treatment ranking for each outcome ………………………………………………………………. 50**](#8alyw7f1xfmm)

[S7.1 Treatment ranking for Height Velocity](#nml10l6umfzw)

[S7.2 Treatment ranking for Height SDS](#fs734tmjkct5)

[S7.3 Treatment ranking for Treatment discontinuation](#1u2xx4109ime)

[S7.4 Treatment ranking for Injection site erythema](#ypd9uee4ifv0)

[S7.5 Treatment ranking for Influenza](#z3744rxdxyq4)

[S7.6 Treatment ranking for Headache](#6gnhnxa6cd4b)

[S7.7 Treatment ranking for Fever](#6xlf0vuwkqvk)

[S7.8 Treatment ranking for hypothyroidism](#dku4w5fi7kvu)

[S7.9 Treatment ranking for Injection site pain](#pxcio1ylwpy3)

[S7.10 Treatment ranking for IGF-1](#rzxithf2nq4n)

[S7.11 Treatment ranking for HbA1C%](#9b8z0pz53fjg)

[S7.12 Treatment ranking for cough](#bjp0lki15o5d)

[S7.13 Treatment ranking for URTI](#wmo5p0vnqesv)

[S7.14 Treatment ranking for FPG](#itjn4hs93lo1)

[**Supplement 8: Certainty of evidence for each outcome …………………………………………………………… 58**](#i9ykb3wv7c6a)

[S8.1 Certainty of evidence for Height Velocity](#7hr0iaczrko4)

[S8.2 Certainty of evidence for Height SDS](#iuoqwzctf1am)

[S8.3 Certainty of evidence for Treatment discontinuation](#z0v2nmikm7pu)

[S8.4 Certainty of evidence for Injection site erythema](#l3kmtpom6xha)

[S8.5 Certainty of evidence for Influenza](#fmf0zx1pil55)

[S8.6 Certainty of evidence for Headache](#5uf8m1qkjx5e)

[S8.7 Certainty of evidence for Fever](#mu8xkv8n2yck)

[S8.8 Certainty of evidence for hypothyroidism](#mzmuf4oeyiqt)

[S8.9 Certainty of evidence for Injection site pain](#ngww5omv7jrw)

[S8.10 Certainty of evidence for IGF-1](#i55g2mj4nlks)

[S8.11 Certainty of evidence for HbA1C%](#kgsymzioqtkq)

[S8.12 Certainty of evidence for cough](#agtzastm4fri)

[S8.13 Certainty of evidence for URTI](#q3nkluhrkz39)

[S8.14 Certainty of evidence for FPG](#fbc9pc9zmgvc)

[**Supplement 9: Network Geometry Summary …………………………………………………………………………158**](#nhah81kjm1bv)

[S9.1 Network Geometry Summary for Height Velocity](#3lpzznu3ylgk)

[S9.2 Network Geometry Summary for Height SDS](#nnd4fem3kkjy)

[S9.3 Network Geometry Summary for Treatment Discontinuation](#8qj9b4cx0zkb)

[S9.4 Network Geometry Summary for Injection Site Erythema](#sr5who3uv04i)

[S9.5 Network Geometry Summary for Influenza](#r5ca9lolrd6k)

[S9.6 Network Geometry Summary for Headache](#l6aiocipxcay)

[S9.7 Network Geometry Summary for Fever](#dcjrobv2gntj)

[S9.8 Network Geometry Summary for hypothyroidism](#jfecqcmnvpad)

[S9.9 Network Geometry Summary for Injection Site Pain](#ht5fbgt02eeu)

[S9.10 Network Geometry Summary for IGF-1](#6jx9khknukhb)

[S9.11 Network Geometry Summary for HbA1C%](#qlce1o7lm0ka)

[S9.12 Network Geometry Summary for cough](#i95tozfxlx9r)

[S9.13 Network Geometry Summary for URTI](#w74cgvrzcek7)

[S9.14 Network Geometry Summary for FPG](#hwqrt8ew8i41)

[**Supplement 10: PRISMA checklist …………………………………………………………………………………… 165**](#ge162r2zob7i)

**Supplement 1: Search Strategy**

From inception to April 15th, 2026

**PubMed Query**: ((((((((((((((((((((lonapegsomatropin) OR (somatrogon)) OR (somapacitan)) OR (Skytrofa)) OR (Ngenla)) OR (Sogroya)) OR (long-acting growth hormone)) OR (long acting growth hormone)) OR (LAGH)) OR (once-weekly growth hormone)) OR (weekly growth hormone)) OR (long-acting somatropin)) OR (sustained-release growth hormone)) OR (depot growth hormone)) OR (PEGylated)) ) OR (Long-Acting Preparations [MeSH])) AND (((((((((((somatropin) OR (recombinant human growth hormone)) OR (rhGH)) OR (Humatrope)) OR (Genotropin)) OR (Genotonorm)) OR (Norditropin)) OR (Saizen)) OR (Omnitrope)) OR (Nutropin AQ)) OR (Zomacton))) AND (((((growth hormone deficiency[MeSH]) OR (pituitary dwarfism)) OR (IGHD)) OR (isolated growth hormone deficiency)) OR (growth hormone deficiency[tiab]))) AND ((((((((((((compare) OR (comparison)) OR (versus)) OR (vs)) OR (noninferior)) OR (randomized)) OR (randomised)) OR (trial)) OR (head-to-head)) OR (open-label)) OR (efficacy)) OR (effectiveness))) NOT ((animals[MeSH]) NOT (humans))

**Embase:**

('lonapegsomatropin'/exp OR 'somatrogon'/exp OR 'somapacitan'/exp OR skytrofa:ti,ab OR ngenla:ti,ab OR sogroya:ti,ab OR 'long acting growth hormone':ti,ab OR lagh:ti,ab OR 'once weekly growth hormone':ti,ab OR 'weekly growth hormone':ti,ab OR 'long acting somatropin':ti,ab OR 'sustained release growth hormone':ti,ab OR 'depot growth hormone':ti,ab OR pegylated:ti,ab)AND('somatropin'/exp OR 'recombinant human growth hormone':ti,ab OR rhgh:ti,ab OR humatrope:ti,ab OR genotropin:ti,ab OR norditropin:ti,ab OR saizen:ti,ab OR omnitrope:ti,ab OR 'nutropin aq':ti,ab OR zomacton:ti,ab)AND('growth hormone deficiency'/exp OR 'pituitary dwarfism':ti,ab OR ighd:ti,ab OR 'isolated growth hormone deficiency':ti,ab)

AND ('comparative study'/exp OR comparison:ti,ab OR versus:ti,ab OR vs:ti,ab OR noninferior:ti,ab OR randomized:ti,ab OR randomised:ti,ab OR trial:ti,ab OR 'head to head':ti,ab OR open-label:ti,ab OR efficacy:ti,ab OR effectiveness:ti,ab) NOT ('animal'/exp NOT 'human'/exp)

**Cochrane Query:**

("lonapegsomatropin" OR "somatrogon" OR "somapacitan" OR "Skytrofa" OR "Ngenla" OR "Sogroya" OR "long-acting growth hormone" OR "LAGH" OR "once-weekly growth hormone" OR "weekly growth hormone" OR "long-acting somatropin" OR "sustained-release growth hormone" OR "depot growth hormone" OR "PEGylated")AND

("somatropin" OR "recombinant human growth hormone" OR "rhGH" OR "Humatrope" OR "Genotropin" OR "Genotonorm" OR "Norditropin" OR "Saizen" OR "Omnitrope" OR "Nutropin AQ" OR "Zomacton")AND("growth hormone deficiency" OR "pituitary dwarfism" OR "IGHD" OR "isolated growth hormone deficiency") AND ("comparison" OR "versus" OR "vs" OR "noninferior" OR "randomized" OR "randomised" OR "trial" OR "head-to-head" OR "open-label" OR "efficacy" OR "effectiveness")

**Scopus Query:**

TITLE-ABS-KEY (lonapegsomatropin OR somatrogon OR somapacitan OR Skytrofa OR Ngenla OR Sogroya OR "long acting growth hormone" OR LAGH OR "once weekly growth hormone" OR "weekly growth hormone" OR "long acting somatropin" OR "sustained release growth hormone" OR "depot growth hormone" OR PEGylated) AND TITLE-ABS-KEY (somatropin OR "recombinant human growth hormone" OR rhGH OR Humatrope OR Genotropin OR Genotonorm OR Norditropin OR Saizen OR Omnitrope OR "Nutropin AQ" OR Zomacton)

AND TITLE-ABS-KEY ("growth hormone deficiency" OR "pituitary dwarfism" OR IGHD OR "isolated growth hormone deficiency") AND TITLE-ABS-KEY (compare OR comparison OR versus OR vs OR noninferior OR randomized OR randomised OR trial OR "head to head" OR "open-label" OR efficacy OR effectiveness)

**Supplement 2: Study Characteristics Table**

| **Study ID** | **Trial Phase** | **Year** | **NCT Code** | **Intervention, dose** | **Comparator** | **Population Included** | **Population Excluded** | **Number of participants (n)** | **Duration (weeks)** |
| --- | --- | --- | --- | --- | --- | --- | --- | --- | --- |
| Luo, 2017_a | Phase II | 2017 | NCT01342146 | PEG-rhGH, 0.1 mg/kg/week ,0.2 mg/kg/wk | Daily rhGH, 0.25 mg/  kg/wk | Confirmed GH deficiency: GH peak <7.0 ng/mL in two different stimulation tests. Delayed bone age: ≤9 years (girls) or ≤10 years (boys) and at least 2 years behind chronological age. Short stature and slow growth: height SDS < −2 and height velocity <4.0 cm/year. Prepubertal: testicular volume <4 mL (boys) or Tanner stage 1 breasts (girls)  Age >3 years. Patients with multiple pituitary hormone deficiencies included only if other hormones were well controlled with treatment | Chronic diseases (including hematological diseases or  malignancies) or have participated in other clinical trials. | 108 | 25 |
| Luo, 2017_b | Phase III | 2017 | NCT01495468 | PEG-rhGH, 0.2 mg/kg/  wk | Daily rhGH, 0.25 mg/kg/wk | Confirmed GH deficiency: GH peak <7.0 ng/mL in two different stimulation tests  Delayed bone age: ≤9 years (girls) or ≤10 years (boys) and at least 2 years behind chronological age  Short stature and slow growth: height SDS < −2 and height velocity <4.0 cm/year  Prepubertal: testicular volume <4 mL (boys) or Tanner stage 1 breasts (girls)  Age >3 years  Patients with multiple pituitary hormone deficiencies included only if other hormones were well controlled with treatment | Chronic diseases (including hematological diseases or  malignancies) or have participated in other clinical trials. | 343 | 25 |
| Chatelain, 2017 | Phase 2 | 2017 | NCT01947907. | TransCon GH, 0.14, 0.21, or 0.30 mg GH/kg/wk (cohorts 1 to 3) | Genotropin, 0.03 mg GH/kg/d | Male and female prepubertal children with growth hormone deficiency (GHD). Tanner stage 1, Boys aged 3–12 years, girls aged 3–11 years. Diagnosis of GHD based on auxological and biological criteria. Short stature: height ≤ –2.0 SD for age and sex, Inadequate height velocity: ≤ –1.0 SD for age and sex, Body mass index (BMI) within ±2.0 SD for age and sex, Bone age not greater than chronological age, Determined by X-ray of, the left hand and wrist, Assessed by a central bone age reader. Biological criteria: Two GH stimulation tests with peak GH ≤ 10 ng/mL;Second test performed during screening and centrally assayed;Baseline IGF-1 ≤ –1.0 SD for age- and sex-matched norms. | Prior treatment with growth hormone (GH) or IGF-1  Psychosocial dwarfism  Idiopathic short stature or other causes of short stature  Cranial tumor detected by head MRI  GHD secondary to malignancy  Abnormal fundoscopy  Abnormal SHOX1 gene analysis  Turner syndrome confirmed by karyotype  Presence of anti-GH binding antibodies  Closed epiphyses | 53 | 26 |
| Deal, 2022 | Phase 3 | 2022 | NCT02968004 | Somatrogon 0.25,0.48, and 0.66 mg/kg/wk | Daily somatropin, 0.24 mg/kg/wk | Prepubertal children with GHD. Boys aged 3–11 years, Girls aged 3–10 years. Impaired growth: Height velocity (HV) < 25th percentile for age (< −0.7 SDS); Baseline IGF-1 ≤ −1 SDS for age and sex. No prior rhGH treatment; Height not required to be < −2 SDS. IGF-1 measured using the same validated assay across all laboratories. GHD confirmed by 2 different GH stimulation tests. Peak plasma GH ≤ 10 ng/mL. Children with congenital multiple pituitary hormone deficiencies eligible if: Hydrocortisone and/or L-thyroxine doses stable for ≥ 3 months; Children treated for ADHD eligible if medication stable for ≥ 3 months | Cancer; Prior radiation therapy or chemotherapy  BMI < −2 SDS (age- and sex-standardized)  Presence of anti-rhGH antibodies at screening  Psychosocial dwarfism; Turner syndrome, Laron syndrome  Noonan syndrome, Prader–Willi syndrome, Silver–Russell syndrome  SHOX mutations or deletions, Skeletal dysplasia, Born small for gestational age  Birth weight and/or length < −2 SDS for gestational age, Type 1 or type 2 diabetes mellitus if:  Not receiving standard of care. Noncompliant with treatment. In poor metabolic control | 228 | 52 |
| Du, 2022 | Phase 1 | 2022 | NR | PEG-rhGH, 0.12, 0.2 mg/kg/wk | Daily rhGH, 0.28 mg/kg/wk | Short stature: height ≤ −2.0 SD for age and sex; Reduced height velocity (HV): ≤ −2.0 SD for age and sex; BMI within ±2.0 SD for age and sex  Bone age ≤ chronological age; Assessed by left hand and wrist X-ray; Read by a central bone age reader; Two different GH stimulation tests on separate days; Peak GH < 10 ng/mL; Prepubertal status: Boys: testicular volume < 4 mL. Girls: Tanner stage 1 breast development, Age > 3 years  Multiple pituitary hormone deficiency (MPHD);Eligible only if levothyroxine and/or glucocorticoid therapy, Stable for ≥ 3 months prior to enrollment | Chronic diseases;Prior recombinant human GH (rhGH) treatment  Psychosocial dwarfism, Idiopathic short stature, Cranial tumor  GHD secondary to malignancy, SHOX1 gene abnormalities  Turner syndrome, Epiphyseal closure | 71 | 52 |
| Fu, 2025 | Phase 3 | 2025 | NCT04970654 | Somapacitan, 0.16 mg/kg/wk | Daily GH, 0.034 mg/kg/day | For Girls: Tanner stage 1 for breast development (no palpable glandular breast tissue), Age 2.5–10 years at screening  For Boys: Testes volume <4 mL, Age 2.5–11 years at screening  For All Children:Treatment-naïve for GH (growth hormone) or IGF-I, Confirmed growth hormone deficiency (GHD) diagnosis via two different GH stimulation tests within 12 months before randomization. Peak GH ≤10.0 ng/mL (WHO International Somatropin 98/574 standard), Impaired height: ≥2 SD below the mean, Impaired height velocity (HV):<7 cm/year if aged 2.5–3 years;<5 cm/year if aged ≥3 years; Bone age (BA) < chronological age (CA); IGF-I levels < −1.0 SD | Had any clinically significant abnormality that could affect growth or make growth evaluation unreliable (especially standing height measurements).Were born small for gestational age (SGA).  diabetes mellitus. ADHD. Were taking medications that affect growth, e.g., methylphenidate for ADHD.History or current presence of malignancy or intracranial tumors. | 110 | 52 |
| Garner, 2023 | Phase 3 | 2023 | NCT03811535 | Somapacitan, 0.16 mg/kg/wk | Daily GH, 0.034 mg/kg/day | Girls: Tanner stage 1 breast development, age 2.5–10 years  Boys: Testes volume <4 mL, age 2.5–11 years. GH- or IGF-I treatment-naïve. Confirmed GHD via two GH stimulation tests (peak GH ≤10 ng/mL)  Impaired height (≥2 SD below mean). Impaired height velocity: <7 cm/year (age 2.5–3) or <5 cm/year (age ≥3)  Bone age < chronological age. IGF-I < −1.0 SDS | Clinically significant abnormality affecting growth or growth evaluation  Born small for gestational age  Diabetes mellitus  ADHD diagnosis  Treatment with medications affecting growth (e.g., methylphenidate)  History or presence of malignancy or intracranial tumor | 128 | 52 |
| Horikawa, 2022 | Phase 3 | 2022 | NCT03874013 | Somatrogon, 0.25, 0.48, or 0.66 mg/  kg/wk | Genotropin, 0.24 mg/kg/wk | Prepubertal children: Boys: 3 to <11 years, Girls: 3 to <10 years  Confirmed diagnosis of growth hormone deficiency (GHD)  Height SDS ≤ −2. Impaired height velocity (HV) below the 25th percentile for age  Baseline IGF-1 ≤ −1 SDS (at least 1 SD below age- and sex-adjusted mean)  No prior recombinant human GH (rhGH) therapy. GHD confirmed by 2 different GH provocation tests:  Peak serum GH ≤6.0 ng/mL Or ≤16 ng/mL if GH-releasing peptide-2 provocation test | Cancer, or previous radiation therapy or chemotherapy. Malnutrition (BMI < −2 SDS for age and sex). Born small for gestational age, Presence of anti-hGH antibodies at screening  Diabetes mellitus or psychosocial dwarfism. Known or suspected chromosomal or genetic/epigenetic disorders, including:Turner syndrome, Laron syndrome, Noonan syndrome  Prader-Willi syndrome, Silver-Russell syndrome, SHOX mutations/deletions, Skeletal dysplasias | 44 | 52 |
| Liang, 2022 | Phase 1 | 2022 | NCT04513171 | YPEG-rhGH, 100, 120, and 140 mg/kg/wk | Daily rhGH, 35 mg/kg/day | Confirmed GHD: Height SDS (HTSDS) < –2 based on Chinese population standards for age. Height velocity (HV) ≤ 5.0 cm/year, Bone age (BA) delayed > 2 years compared to chronological age (CA). Peak GH level <10 ng/mL in two different GH stimulation tests, IGF-1 level below the age-appropriate median Older than 3 years; Prepubertal (Tanner stage 1):Boys: pubic hair Tanner stage 1, testis volume <4 mL  Girls: breast Tanner stage 1, pubic hair Tanner stage 1, Maximum age: 10 years for girls, 11 years for boys  Proportionate short stature with normal intelligence | Prior administration of recombinant human growth hormone (rhGH). Sex hormone treatments, Malignancy and/or intracranial tumor, Severe allergic constitution, Idiopathic short stature, Turner syndrome, Thyroid hormone deficiency, Hypoadrenalism, Antidiuretic hormone deficiency. Liver dysfunction; Diabetes; Malnutrition; Deformities; Scoliosis with Cobb’s angle > 15 | 40 | 12 |
| Liang, 2024 | Phase 3 | 2024 | NCT04513171 | Pegpesen, 0.14 mg/kg/wk | Daily rhGH, 0.245 mg/kg/wk | Confirmed diagnosis of GHD before screening, defined by: Height SDS (HTSDS) < −2 (Chinese population standard)  Height velocity (HV) ≤ 5.0 cm/year, Bone age (BA) delayed > 2 years vs chronological age (CA), Peak GH < 10 ng/mL on two different GH stimulation tests, IGF-1 level below the age-appropriate median. Age: > 3 years. Prepubertal status (Tanner stage 1); Boys: pubic hair Tanner 1, testicular volume < 4 mL, age ≤ 11 years. Girls: breast and pubic hair Tanner 1, age ≤ 10 years, Proportionate short stature, Normal intelligence | Prior treatment with: Recombinant human GH (rhGH), Sex hormone therapy  Malignancy, Intracranial tumor, Severe allergic constitution, Idiopathic short stature  Turner syndrome, Thyroid hormone deficiency, Hypoadrenalism, Antidiuretic hormone deficiency  Liver dysfunction, Diabetes, Malnutrition, Scoliosis with Cobb’s angle > 15. | 391 | 52 |
| Maniatis, 2022_b | Phase 3 | 2022 | NCT03344458 | Lonapegsomatropin, 0.24 mg hGH/kg/wk | None | Males and females, 6 months to 17 years, Tanner stage <5. Diagnosed with Growth Hormone Deficiency (GHD). Clinical Indicators of GHD (at least 1 required): Two GH stimulation tests with peak GH ≤10 ng/mL. ≤ −2 height SDS or ≥1.5 height SDS below midparental height IGF-1 ≤ −1 SDS. Delayed bone age: ≥6 months relative to chronological age Additional pituitary hormone deficiency, Congenital hypopituitarism. Prior Treatment Requirements: Ages 3–17: Previously treated with daily somatropin ≥0.20 mg/kg/week for 13–130 weeks, Ages 6 months–3 years: Treated ≤130 weeks or treatment-naïve | Weight: <5.5 kg or >80 kg, malignant disease, Clinically significant abnormalities or concomitant medications affecting growth (except hormone replacement for hypopituitarism), Poorly controlled diabetes mellitus, Known neutralizing antibodies against somatropin, Need for high-dose inhaled glucocorticoid therapy, Closed epiphyses. Prior exposure to investigational GH or participation in another trial within 30 day | 298 | 104 |
| Maniatis, 2022 | Phase 3 | 2022 | NCT03305016 | Lonapegsomatropin, 0.24 mg hGH/kg/wk | None | 6 months to 17 years old (Tanner stage <5) diagnosed with GHD. At least 1 of the following before initiating daily somatropin therapy: 2 GH stimulation tests with peak GH levels ≤10 ng/mL, impaired height (≤ −2 height SDS and/or ≥1.5 height SDS below midparental height), IGF-1 ≤ −1 SDS, delayed bone age (≥6 months relative to chronologic age), diagnosis of an additional pituitary hormone deficiency, and/or congenital hypopituitarism. | weighed <5.5 or >80 kg, malignant disease, had any clinically significant abnormality or concomitant medication that may affect growth (hormone replacement for hypopituitarism was allowed), had poorly controlled diabetes mellitus, had known neutralizing antibodies against somatropin, required high-dose inhaled glucocorticoid therapy, had closed epiphyses, had prior exposure to investigational GH or participated in another trial within 30 days. | 146 | 26 |
| Maniatis, 2025 | Phase 3 | 2025 | NCT03344458 | Lonapegsomatropin, 0.24 mg hGH/kg/wk | None | Participants who completed the heiGHt or fliGHt trial and met all other eligibility criteria | closed epiphyses (defined as bone age >14.0 years for females and >16.0 for males), poorly controlled diabetes mellitus (HbA1c ≥8.0%) or diabetic complications, or other major medical conditions at enliGHten baseline | 298 | 272 |
| Miller, 2022 | Phase 3 | 2022 | NCT03811535 | Somapacitan, 0.16 mg/kg/wk) | Daily GH, 0.034 mg/kg/d | Girls—Tanner stage 1 for breast development and age between 2.5 years and 10 years at screening; boys: testes volume <4 mL and age between 2.5 years and 11 years at screening. For all children: impaired height (at least 2.0 SD below the mean) for chronological age (CA) and sex; impaired height velocity (HV) (annualized HV below the 25th percentile) for CA and sex; and IGF-I <−1.0 SDS at screening. | Any clinically significant abnormality affecting growth or growth assessment  Born small for gestational age, Diabetes mellitus, ADHD diagnosis or treatment affecting growth (e.g., methylphenidate), malignancy or intracranial tumour. | 200 | 52 |
| Miller, 2023 | Phase 3 | 2023 | NCT03811535 | Somapacitan, 0.16 mg/kg/wk) | Daily GH, 0.034 mg/kg/d | Girls—Tanner stage 1 for breast development and age between 2.5 years and 10 years at screening; boys: testes volume <4 mL and age between 2.5 years and 11 years at screening. For all children: impaired height (at least 2.0 SD below the mean) for chronological age (CA) and sex; impaired height velocity (HV) (annualized HV below the 25th percentile) for CA and sex; and IGF-I <−1.0 SDS at screening. | Any clinically significant abnormality affecting growth or growth assessment  Born small for gestational age, Diabetes mellitus, ADHD diagnosis or treatment affecting growth (e.g., methylphenidate), malignancy or intracranial tumour. | 200 | 104 |
| Miller, 2025 | Phase 3 | 2025 | NCT03811535 | Somapacitan, 0.16 mg/kg/wk) | Daily GH, 0.034 mg/kg/d | Girls—Tanner stage 1 for breast development and age between 2.5 years and 10 years at screening; boys: testes volume <4 mL and age between 2.5 years and 11 years at screening. For all children: impaired height (at least 2.0 SD below the mean) for chronological age (CA) and sex; impaired height velocity (HV) (annualized HV below the 25th percentile) for CA and sex; and IGF-I <−1.0 SDS at screening. | Any clinically significant abnormality affecting growth or growth assessment  Born small for gestational age, Diabetes mellitus, ADHD diagnosis or treatment affecting growth (e.g., methylphenidate), malignancy or intracranial tumour. | 200 | 208 |
| Sävendahl,2020 | Phase 2 | 2020 | NCT02616562 | Somapacitan, 0.04, 0.08, or 0.16 mg/  kg/wk | Daily GH, 0.034 mg/kg/d | Confirmed GHD diagnosis Within 12 months before, Determined by 2 different unprimed GH stimulation tests (peak GH ≤7.0 ng/mL) for most children, For children with ≥3 pituitary hormone deficiencies: only 1 GH stimulation test required. In Japan: 1 GH stimulation test sufficient if intracranial organic disease or symptomatic hypoglycemia; 2 GH stimulation tests for other patients (peak GH ≤6 ng/mL using recombinant GH standard). Height ≥2 SD below mean for chronological age (below 3rd percentile), Annualized height velocity (HV) below 25th percentile for age or ≤ –0.7 SD, calculated over 6–18 months. No prior GH or IGF-I therapy | Clinically significant abnormalities (chromosomal aneuploidy, gene mutations, syndromes causing short stature like Turner, Laron, Noonan), congenital skeletal abnormalities (Russell-Silver syndrome, skeletal dysplasias), Significant spinal abnormalities (scoliosis, kyphosis, spina bifida variants). Small for gestational age (birth weight and/or length < –2 SD)  Other treatments affecting growth: E.g., methylphenidate for ADHD. Malignancy or intracranial tumor. | 59 | 52 |
| Sävendahl,2021 | Phase 2 | 2021 | NCT02616562 | Somapacitan, 0.04, 0.08, or 0.16 mg/  kg/wk | Daily GH, 0.034 mg/kg/d | Prepubertal children with confirmed GHD. Diagnosis confirmation: Within 12 months prior to screening, Two GH stimulation tests showing peak GH ≤7.0 ng/mL, For children with ≥3 pituitary hormone deficiencies, only one GH stimulation test was required. Prior treatment: No previous GH or IGF-I therapy | Malignancy chronic illness affecting growth, syndromes affecting growth (Turner, Noonan, etc.), Small for gestational age at birth, Severe malnutrition | 59 | 156 |
| Thornton,2021 | Phase 3 | 2021 | NCT02781727 | Lonapegsomatropin 0.24 mg/kg/week | Somatropin 0.24 mg/kg/week | Treatment-naïve, prepubertal children with GHD (males 3-12y, females 3-11y) | Prior GH/IGF-1 therapy, history of malignancy, non-GHD short stature | 161 | 52 |

**Supplement 3: Patient Characteristics Table**

| Study ID | Chrono Age (mean ± SD) | Bone Age (mean ± SD) | Race, % | Male (%) | BMI (kg/m2) (mean ± SD) | Height (mean ± SD) | Weight (mean ± SD) | Height Velocity (mean ± SD) | Height SDS (mean ± SD) | Height Velocity SDS (mean ± SD) | IGF-1 (mean ± SD) | GH Peak (ug/L) (mean ± SD) | HbA1c (Mean ±SD) | Insulin level (mean ± SD) | Fasting Blood Sugar Level (mean ± SD) | Idiopathic % | Organic % | n1 | Treat1, Dose | n2 | Treat2,Dose |
| --- | --- | --- | --- | --- | --- | --- | --- | --- | --- | --- | --- | --- | --- | --- | --- | --- | --- | --- | --- | --- | --- |
| Luo, 2017 | 11.04±3.78 | 6.30±2.37 | NR | 73.19 | NR | 112.26±13.32 | 21.19±6.81 | 2.71±0.86 | -4.60±1.76 | NR | -2.30±0.83 | NR | NR | NR | NR | NR | NR | 63 | PEG-rhGH, 0.1, 0.2mg/kg/week | 34 | Daily rhGH, 0.25mg/kg/week |
| Luo, 2017 | 11.45±3.53 | 7.01±2.23 | NR | 81.6 | NR | 117.28±12.72 | 23.72±6.97 | 2.3±0.86 | -4.49±1.97 | NR | -1.74±1.08 | 2.50±2.35 | 5.36±0.57 | 3.84±3.23 | 4.56±0.60 | NR | NR | 109 | PEG-rhGH ,0.2mg/kg/week | 115 | Daily rhGH, 0.25mg/kg/week |
| Chatelain, 2017 | 7.94±2.53 | 5.33±2.37 | White, 100 | 71.69 | 15.86±1.74 | 109.52±14.59 | 19.38±5.72 | NR | -3.09±0.92 | NR | -2.17±0.78 | 5.61±2.70 | 5.39±0.33 | 4.1±2.39 | 84.66±8.03 | NR | NR | NR | TransCon GH 0.14,0.21,0.30 mg/kg/wk | 32 | Genotropin,0.03 mg GH/kg/d |
| Deal, 2022 | 7.7±1.61 | 5.33± 2.59 | White 74.6,Black 0.9, Asian 20.1, American Indian 0.4, Native Hawaiian 0.4, Other 3.6 | 71.9 | -0.24±1.02 | -2.86±1.28 | -2.53±1.76 | NR | NR | NR | NR | NR | NR | NR | NR | NR | NR | 109 | Somatrogon 0.66 mg/kg/week | 115 | Daily GH ,0.034 mg/kg/day |
| Du, 2022 | 9.42±4.25 | 5.56±2.46 | NR | 50.7 | 16.63±2.96 | 116.4± 14.7 | 25.30±10.27 | NR | -2.99±1.48 | NR | -1.98±0.93 | NR | 5.12±0.27 | 7.88±5.73 | 4.87±0.39 | NR | NR | NR | Combined PEG-rhGH 0.12, 0.20 mg/kg/wk | 23 | Daily rhGH, 0.28 mg/kg/w |
| Fu, 2025 | 6.56±2.15 | NR | Asian 100 | 71.7 | 15.26±1.09 | 107.04± 12.62 | 17.7±4.49 | 3.43±1.38 | -2.78± 0.72 | -3.16±1.77 | -1.58±0.76 | 6.26±2.58 | NR | NR | NR | 94.5 | 3.64 | 74 | Somapacitan 0.16 mg/kg/week | 36 | Daily GH Norditropin, 0.034 mg/kg/day |
| Garner, 2023 | 6.31±2.33 | NR | 63.958 white, 18.562 Asian, 0.3719 Black or African american, 0.628 Not reported, 2.884 Other | 72.47 | 15.63±1.51 | 101.06±12.92 | 16.3±4.61 | 4.37±1.40 | -3.12±1.06 | -2.29±1.41 | -2.15±1 | 4.68±2.58 | NR | 4.1±2.39 | 84.66±8.03 | 56.58 | 7.88 | 76 | Somapacitan 0.16 mg/kg/week | 45 | Daily GH 0.034 mg/kg/day |
| Horikawa, 2022 | 6.03±2.21 | NR | Japanese,100 | 47.7 | NR | NR | NR | NR | -2.57±0.42 | NR | -1.50±0.87 | NR | NR | NR | NR | NR | NR | 22 | Somatrogon 0.25 mg/  kg/week | 22 | Genotropin 0.025 mg/kg/day or 0.175 mg/kg/week, |
| Liang, 2022 | 7.12±2.82 | 4.69±2.72 | NR | 69.78 | 15.02±1.21 | NR | 18.92±4.49 | 3.63±1.47 | -2.71±0.74 | -2.71±0.74 | NR | NR | NR | NR | NR | NR | NR | 31 | YPEG-rhGH , 100 mg/kg/week | 12 | Daily rhGH, 0.035 mg/kg/day |
| Liang, 2024 | 6.79±2.14 | 4.57±10.30 | NR | 66.23 | 15.42±1.55 | 108.89±11.65 | 18.58±4.86 | NR | NR | NR | NR | 105.21±43.36 | NR | NR | NR | NR | NR | 261 | Pegpesen140 μg/kg/week | 130 | Daily rhGH 0.245 mg/kg/week |
| Maniatis, 2022_b | 10.3±3.4 | NR | White 90.6 , 2.7 Unknown, 2.3 Multiple/other, 2.0 Asian, 1.7 Black or African American, 0.7 Native Hawaiian or other Pacific Islander. | 78.9 | NR | NR | NR | NR | -1.6 ±0.9 | NR | 324.9±169.0 | 5.8±2.7 | 5.2±0.3 | NR | NR | NR | NR | 243 | Lonapegsomatropin, 0.24 mg/kg/week | 56 | NA |
| Maniatis, 2022 | 10.66±3.08 | NR | 84.9 White, 4.1 Asian, 2.1 Black or African American, 1.4 Native Hawaiian or other Pacific Islander, 1.4 Multiple, 6.2 Multiple | 75.3 | NA | NR | NR | NA | -1.42±0.84 | NR | NR | NR | NR | NR | NR | NR | NR | 143 | Lonapegsomatropin, 0.24 mg/kg/week | NA | NA |
| Maniatis, 2025 | 10.3±3.4 | 4.69±2.69 | NR | 78.9 | NR | NR | NR | NR | -1.6±0.88 | NR | NR | NR | NR | NR | NR | NR | NR | 298 | Lonapegsomatropin, 0.24 mg/kg/week | NA | NA |
| Miller, 2022 | 6.4±2.3 | NR | 57.0 White, 37 Asian, 1 black or african | 74.5 | 15.7±1.52 | 101.6±13.4 | 16.5±4.72 | 4.2±1.4 | -3.15±1.23 | -2.41±1.52 | -2.13±1.00 | 4.65±2.62 | NR | NR | NR | 88 | 12 | 132 | 0.16 mg/kg/wk | 68 | 0.034 mg/kg/  d |
| Miller, 2023 | 6.4±2.3 | NR | 57.0 White, 37 Asian, 1 black or african | 74.5 | 15.7±1.52 | 101.6±13.4 | 16.5±4.72 | 4.2±1.4 | -3.15±1.23 | -2.41±1.52 | NR | 4.65±2.62 | NR | NR | NR | 88 | 12 | 132 | 0.16 mg/kg/wk | 68 | 0.034 mg/kg/  d |
| Miller, 2025 | 6.4±2.3 | NR | 57.0 White, 37 Asian, 1 black or african | 74.5 | 15.7±1.52 | 101.6±13.4 | 16.5±4.72 | 4.2±1.4 | -3.15±1.23 | -2.41±1.52 | NR | 4.65±2.62 | NR | NR | NR | 88 | 12 | 132 | 0.16 mg/kg/wk | 68 | 0.034 mg/kg/  d |
| Sävendahl,2020 | 5.94±1.92 | 3.33±1.68 | NR | 59.32 | 15.14±1.22 | 97.11±13.62 | 14.62±4.44 | NR | NR | -2.66±1.90 | NR | 3.04±2.12 | NR | NR | NR | 89.83 | 1.63 | 45 | Somapacita 0.04, 0.08, 0.16 mg/kg | 14 | Daily GH 0.034 mg/kg/d |
| Sävendahl,2021 | 5.89±1.92 | NR | NR | 59.52 | 15.14±1.22 | NR | NR | NR | -3.71±1.65 | -2.66±1.90 | NR | 3.62±2.17 | NR | NR | NR | NR | NR | 45 | Somapacita 0.04, 0.08, 0.16 mg/kg | 14 | Daily GH 0.034 mg/kg/d |
| Thornton,2021 | 8.5±2.7 | 5.9±2.6 | white, 94.4 | 82 | 16.2±1.9 | 112.7±14.5 | 21.1±6.6 | NR | −2.93±0.87 | −2.18±2.14 | 81.7±48.8 | 5.8±2.8 | 5.04±0.33 | NR | 87.7±9.54 | 65 | 17 | 105 | Lonapegsomatropin 0.24 mg hGH/kg/wk | 56 | Daily somatropin 0.24 mg hGH/kg/wk |

**Supplement 4: Risk of Bias Table for each outcome**

| **Study ID** | **Domain 1: Randomization Process** | **Domain 2. Deviations from intended interventions** | **Domain 3. Missing outcome data** | **Domain 4. Measurement of the outcome** | **Domain 5. Selection of the reported result** | **Domain 6. Overall Bias** |
| --- | --- | --- | --- | --- | --- | --- |
| Luo et al., 2017 | Low Risk | Low Risk | Low Risk | Low Risk | Low Risk | Low Risk |
| Chatelain et al., 2017 | Low Risk | Low Risk | Low Risk | Low Risk | Low Risk | Low Risk |
| Deal, 2022 | Low Risk | Low Risk | Low Risk | Low Risk | Low Risk | Low Risk |
| Du, 2022 | Some Concerns | Some Concerns | Low Risk | Low Risk | Low Risk | Some Concerns |
| Fu, 2025 | Low Risk | Low Risk | Low Risk | Low Risk | Low Risk | Low Risk |
| Garner, 2023 | Low Risk | Low Risk | Low Risk | Low Risk | Low Risk | Low Risk |
| Horikawa, 2022 | Low Risk | Low Risk | Low Risk | Low Risk | Low Risk | Low Risk |
| Liang, 2022 | Low Risk | Low Risk | Low Risk | Low Risk | Low Risk | Low Risk |
| Liang, 2024 | Low Risk | Low Risk | Low Risk | Some Concerns | Low Risk | Low Risk |
| Maniatis, 2022 | Some Concerns | High Risk | Low Risk | Low Risk | Low Risk | High Risk |
| Maniatis, 2022_b | Some Concerns | High Risk | Some Concerns | Low Risk | Low Risk | High Risk |
| Maniatis, 2025 | Some Concerns | High Risk | High Risk | Low Risk | Low Risk | High Risk |
| Miller, 2022 | Low Risk | Some Concerns | Low Risk | Low Risk | Low Risk | Some Concerns |
| Miller, 2023 | Some Concerns | Low Risk | Low Risk | High Risk | Low Risk | High Risk |
| Miller, 2025 | High Risk | Some Concerns | Low Risk | High Risk | Low Risk | High Risk |
| Savenahl, 2020 | Low Risk | Low Risk | Low Risk | High Risk | Low Risk | High Risk |
| Savenahl, 2021 | High Risk | Low Risk | Some Concerns | High Risk | High Risk | High Risk |
| Thornton,2021 | Low Risk | Low Risk | Low Risk | Low Risk | Low Risk | High Risk |

**Supplementary 5: Publication bias -funnel plot- for each outcome**

S5.1 Publication bias -funnel plot- for Height Velocity


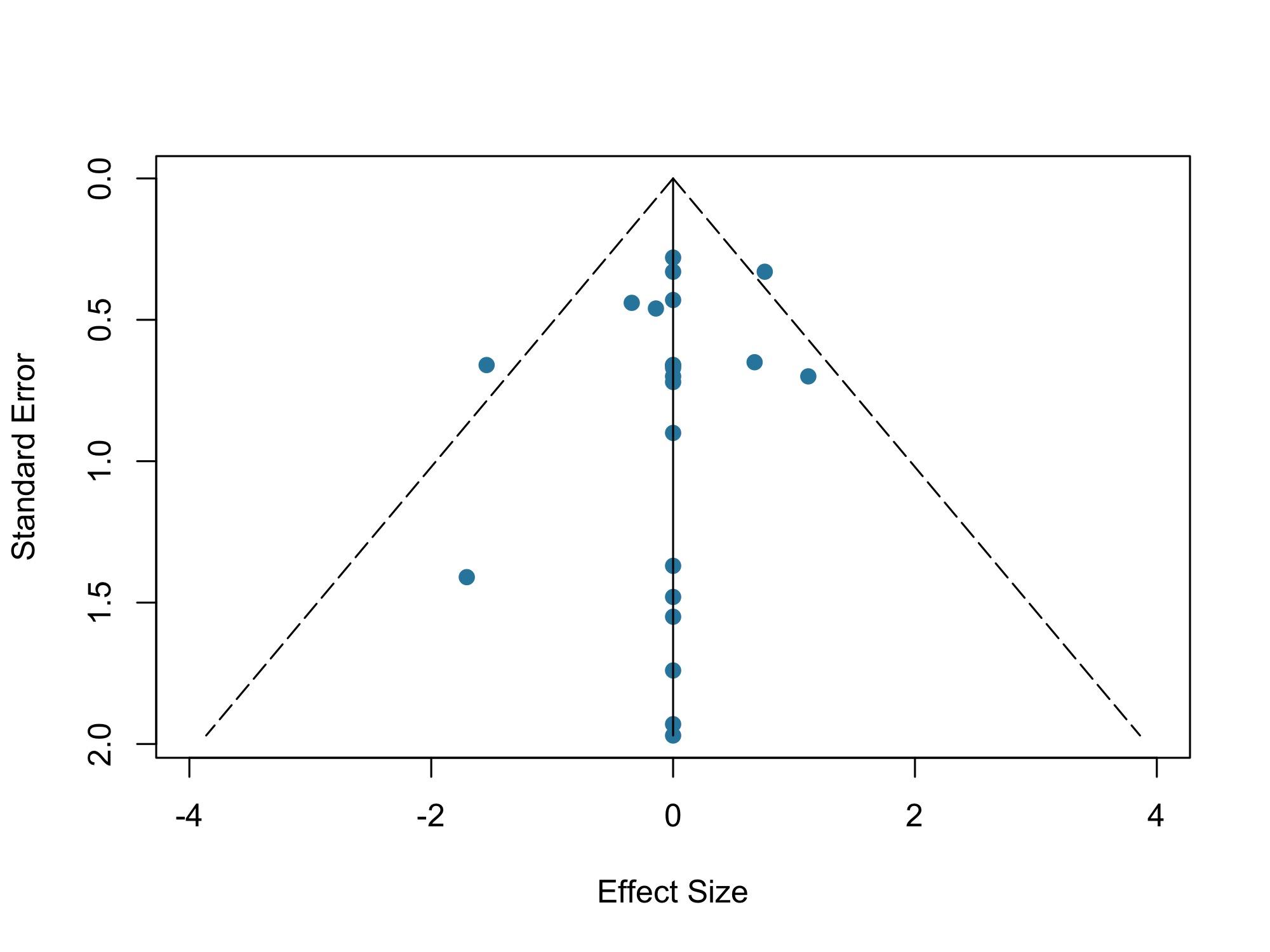


S5.2 Publication bias -funnel plot- for Height SDS


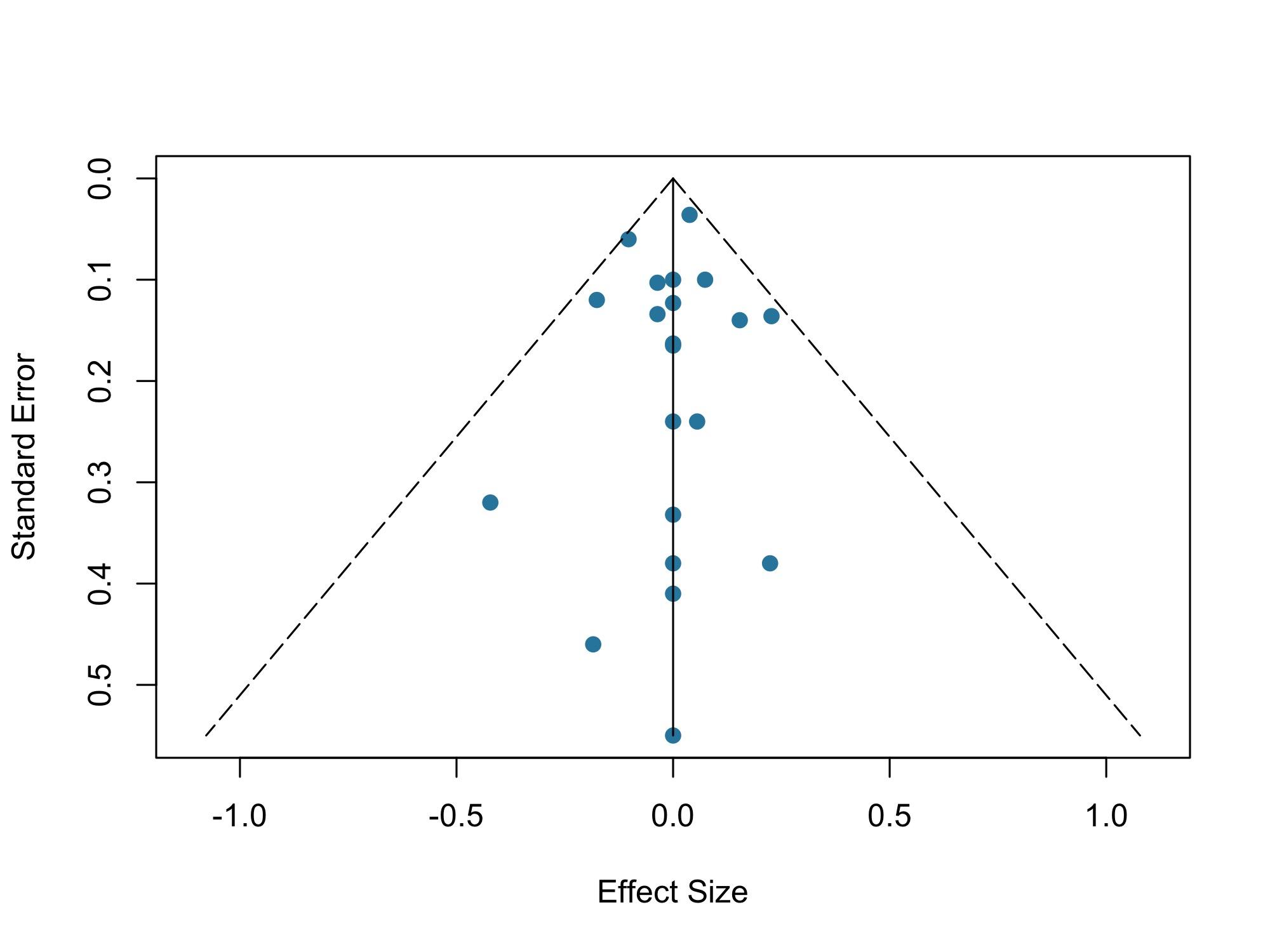


S5.3 Publication bias - funnel plot- for treatment discontinuation


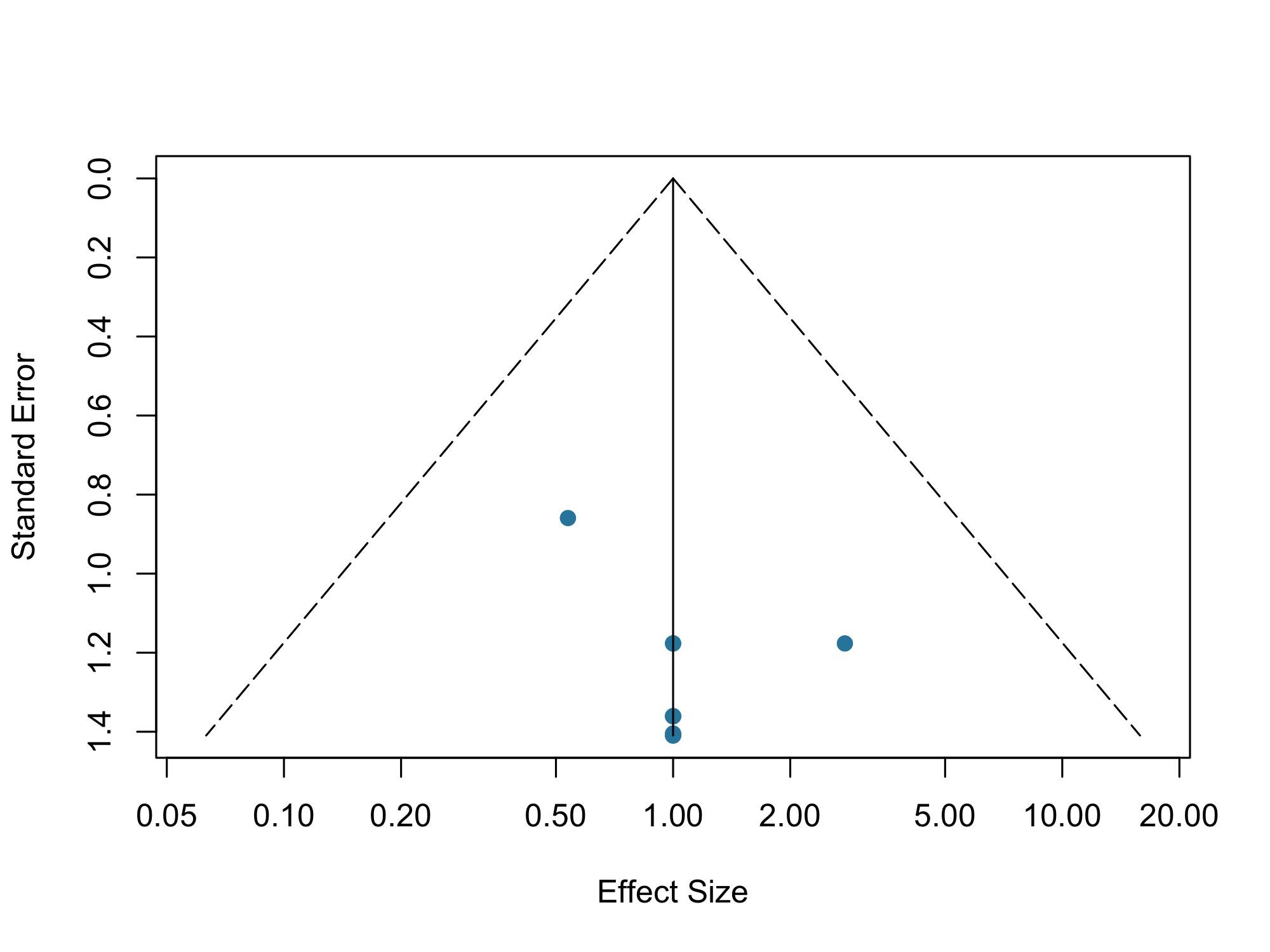


S5.4 Publication bias -funnel plot- for Injection site erythema


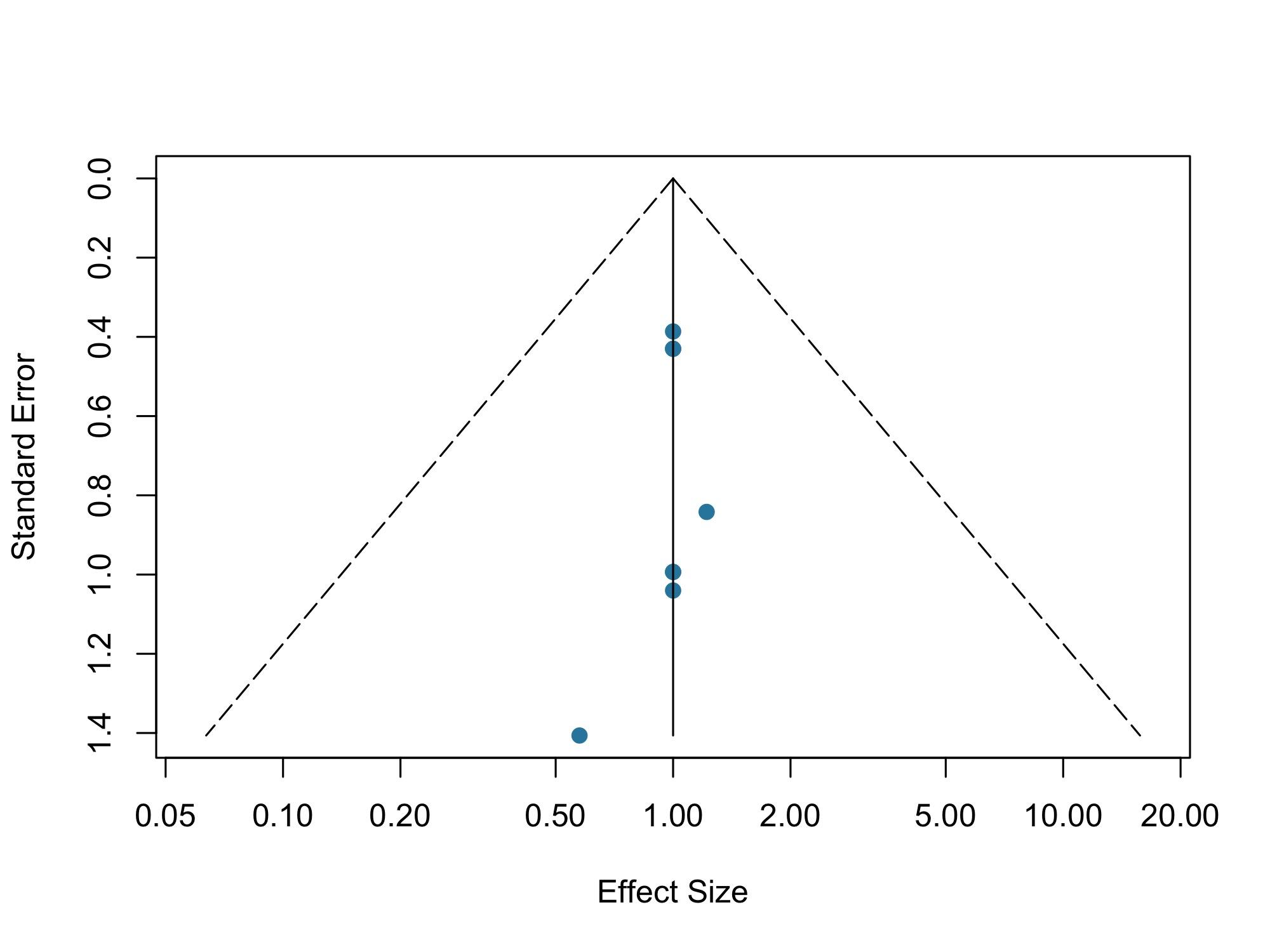


S5.5 Publication bias -funnel plot- for Influenza


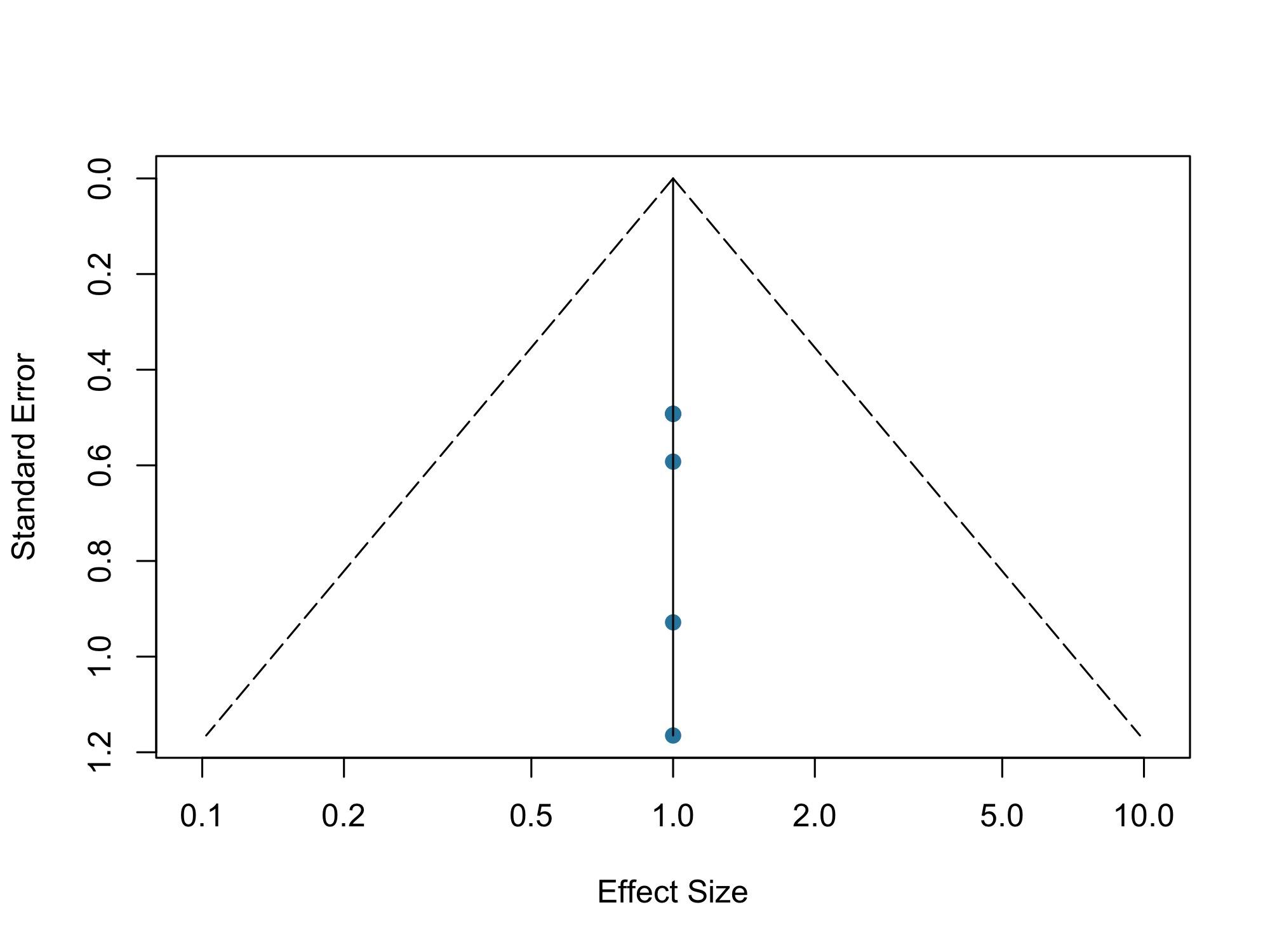


S5.6 Publication bias -funnel plot- for Headache


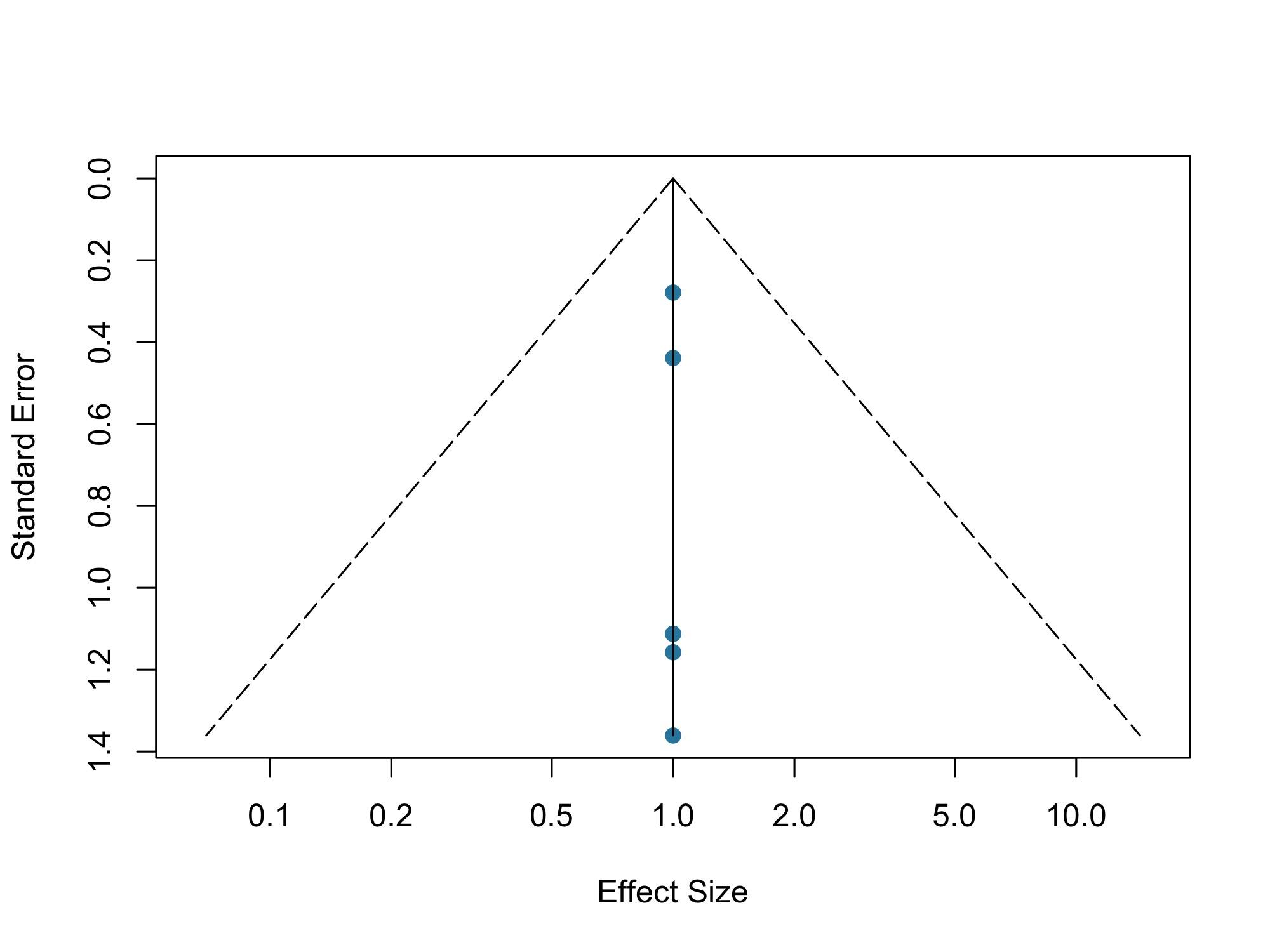


S5.7 Publication bias -funnel plot- for Fever


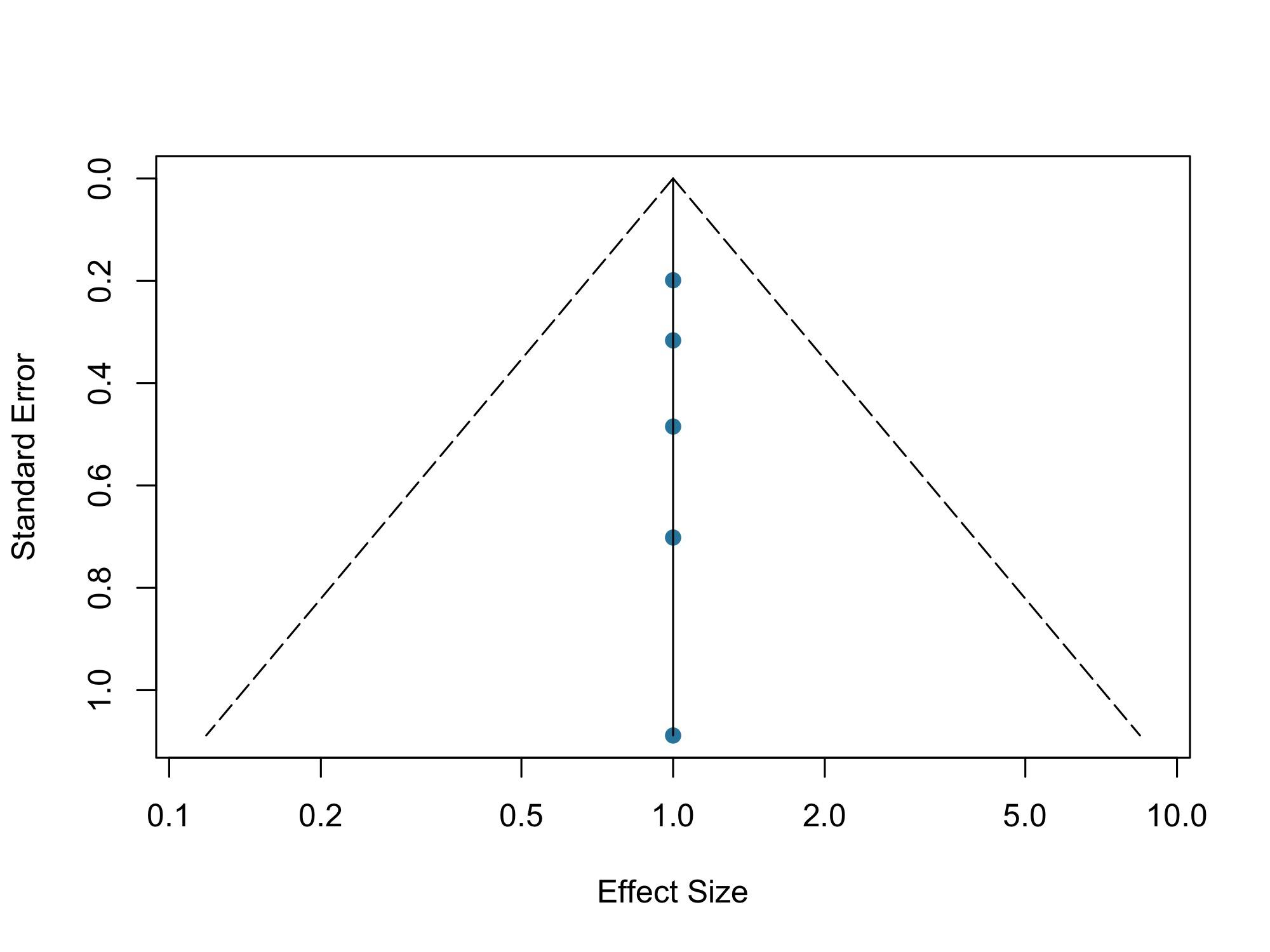


S5.8 Publication bias -funnel plot- for hypothyroidism


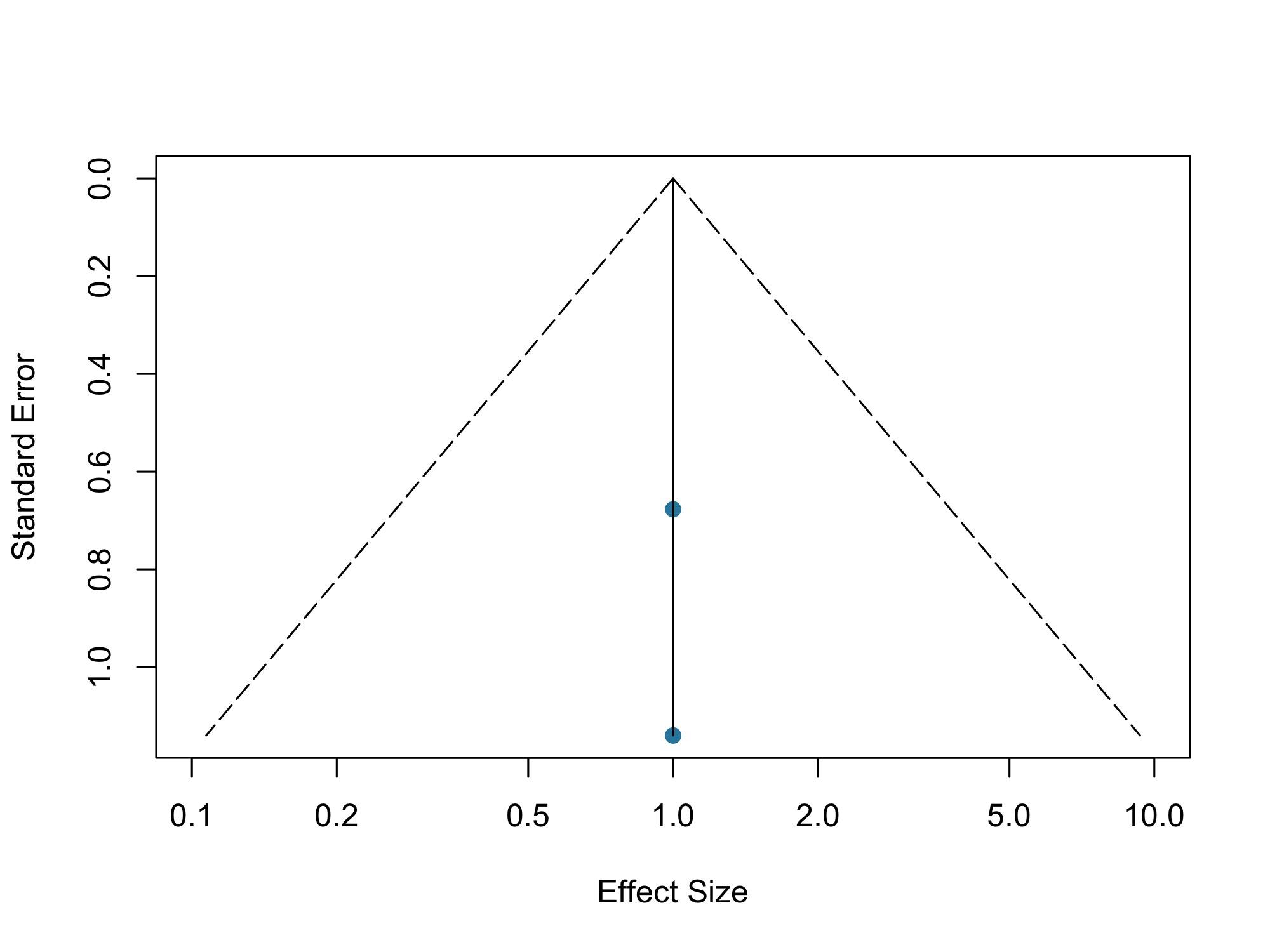


S5.9 Publication bias -funnel plot- for Injection site pain

**
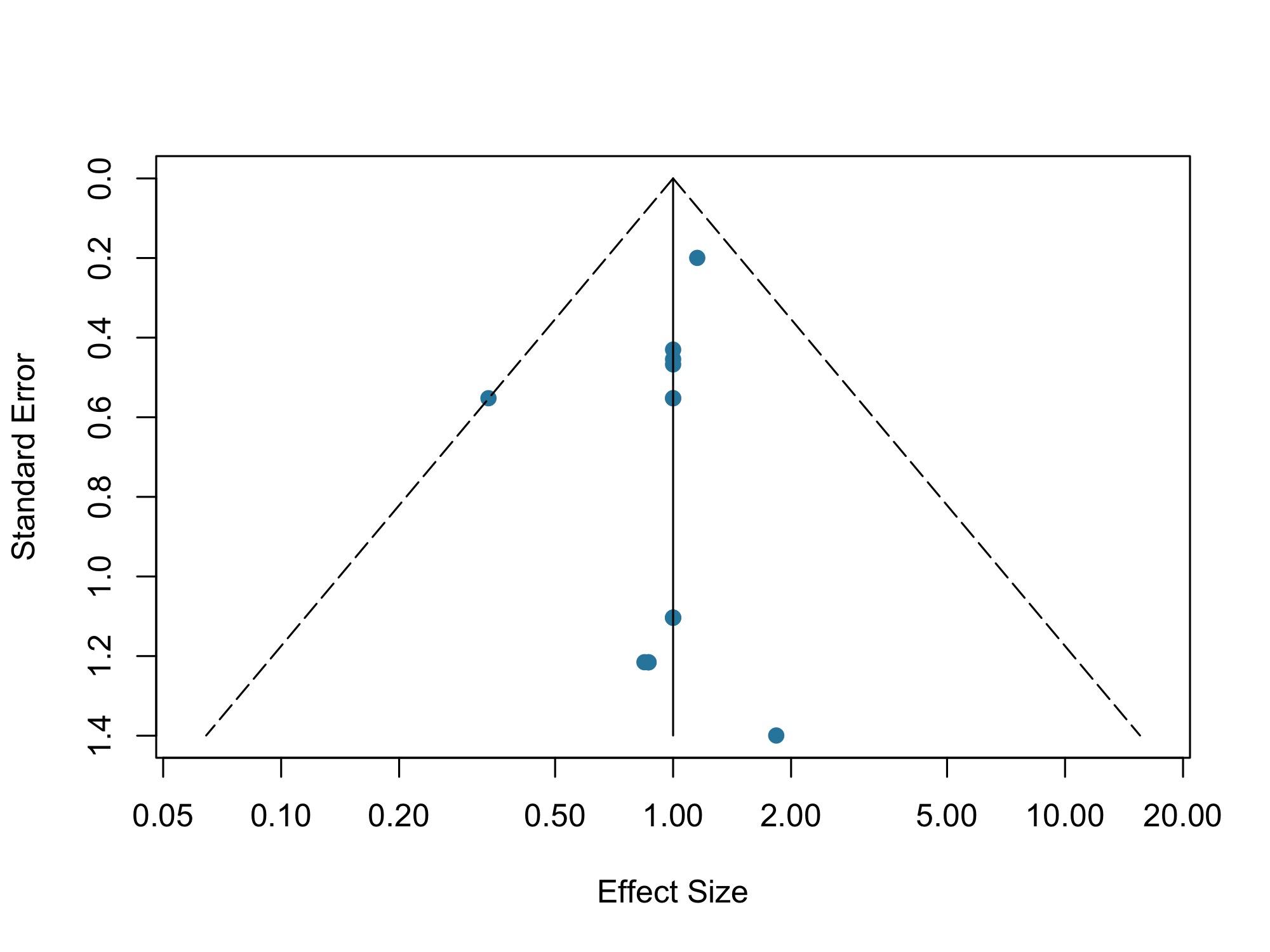
**

S5.10 Publication bias -funnel plot- for IGF-1


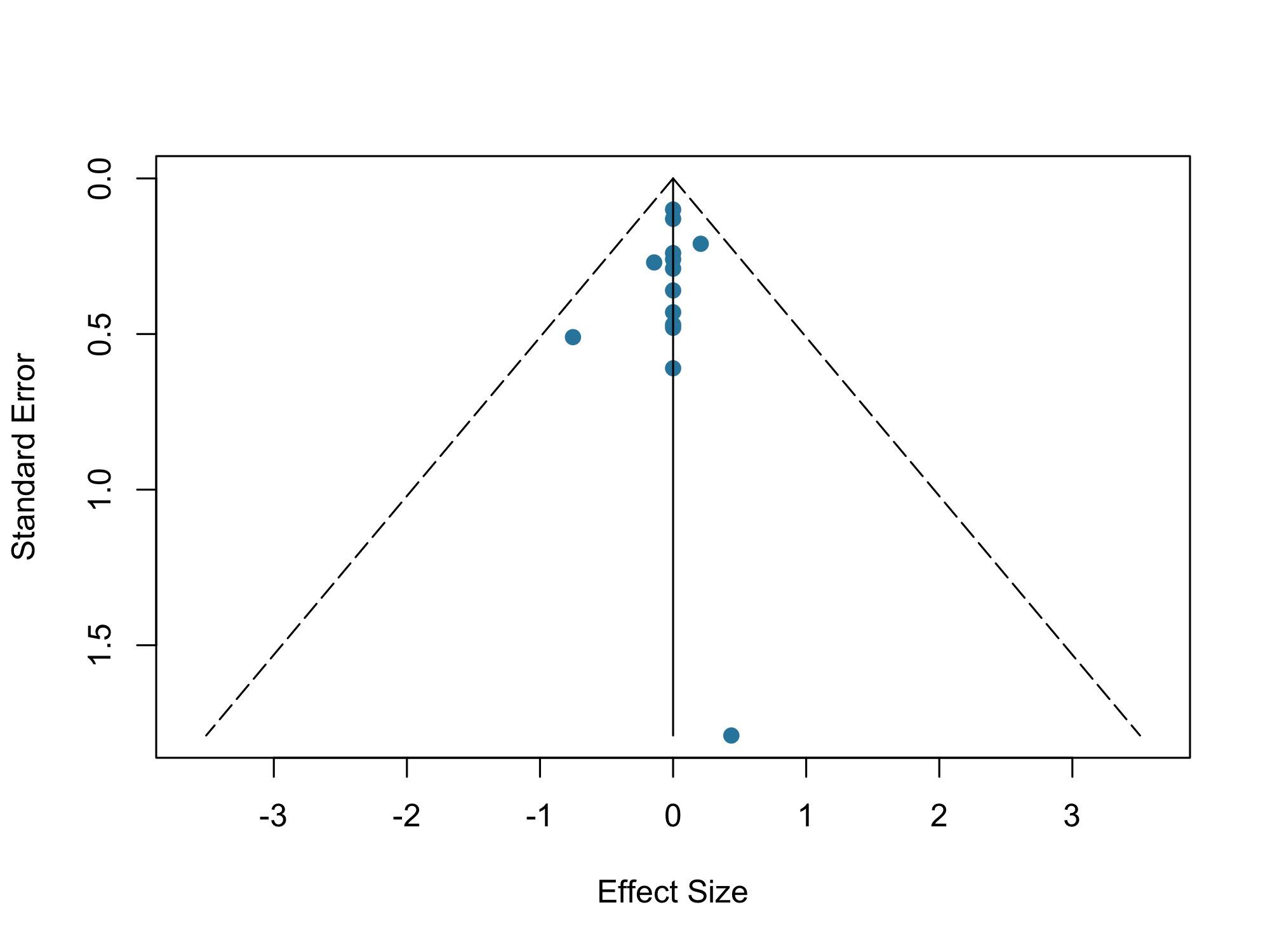


S5.11 Publication bias -funnel plot- for HbA1C%


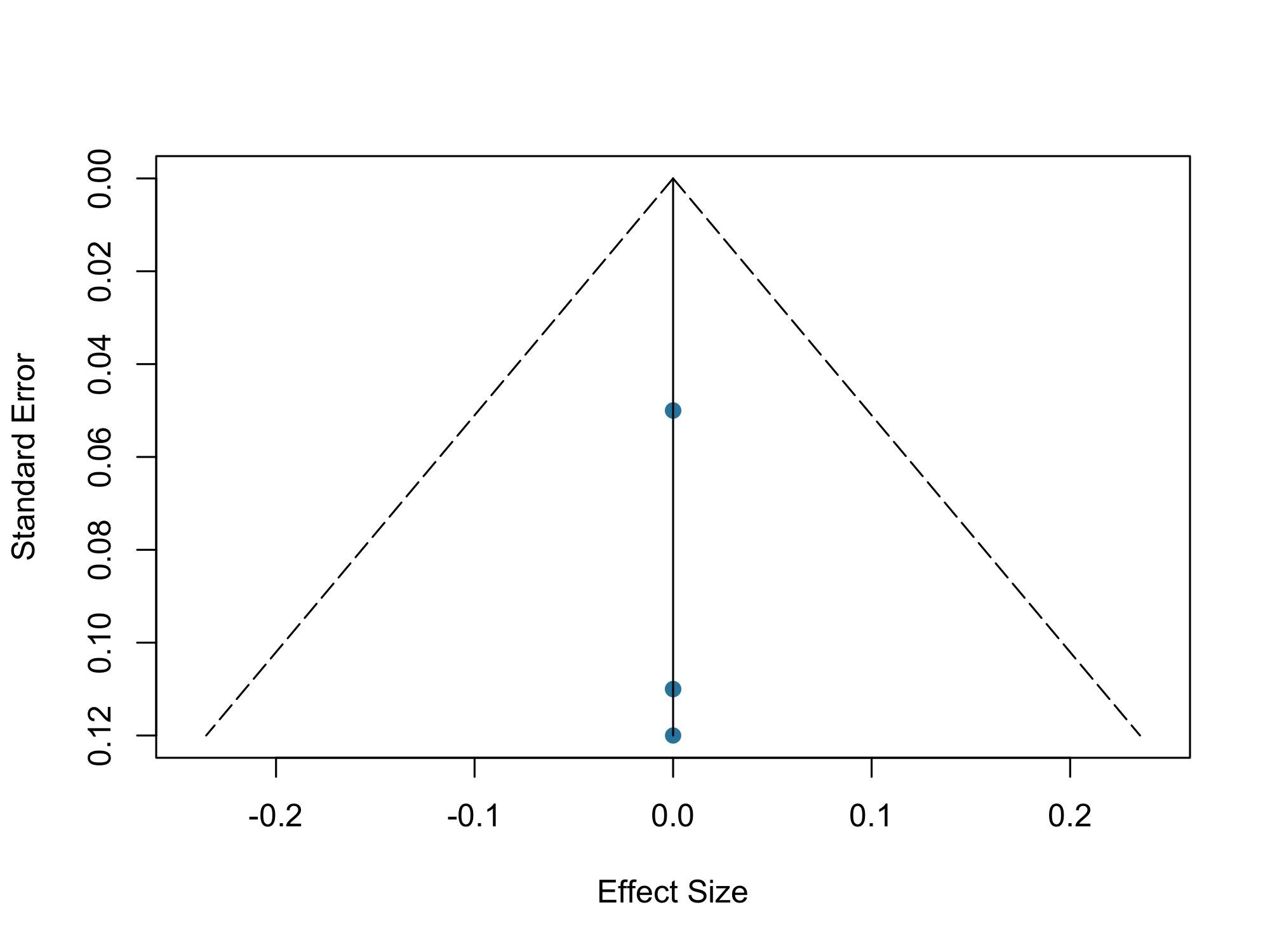


S5.12 Publication bias -funnel plot- for cough


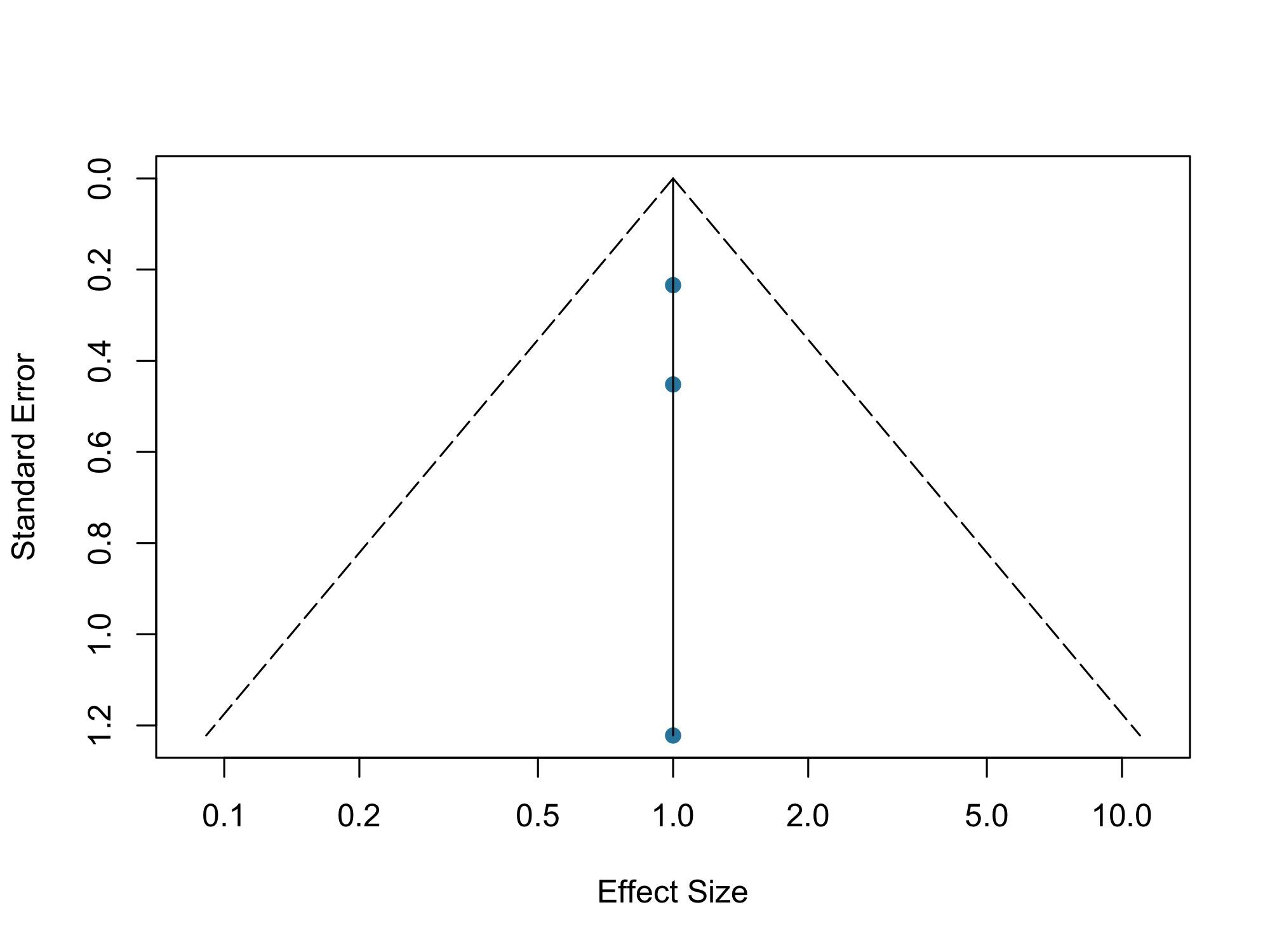


S5.13 Publication bias -funnel plot- for URTI

**
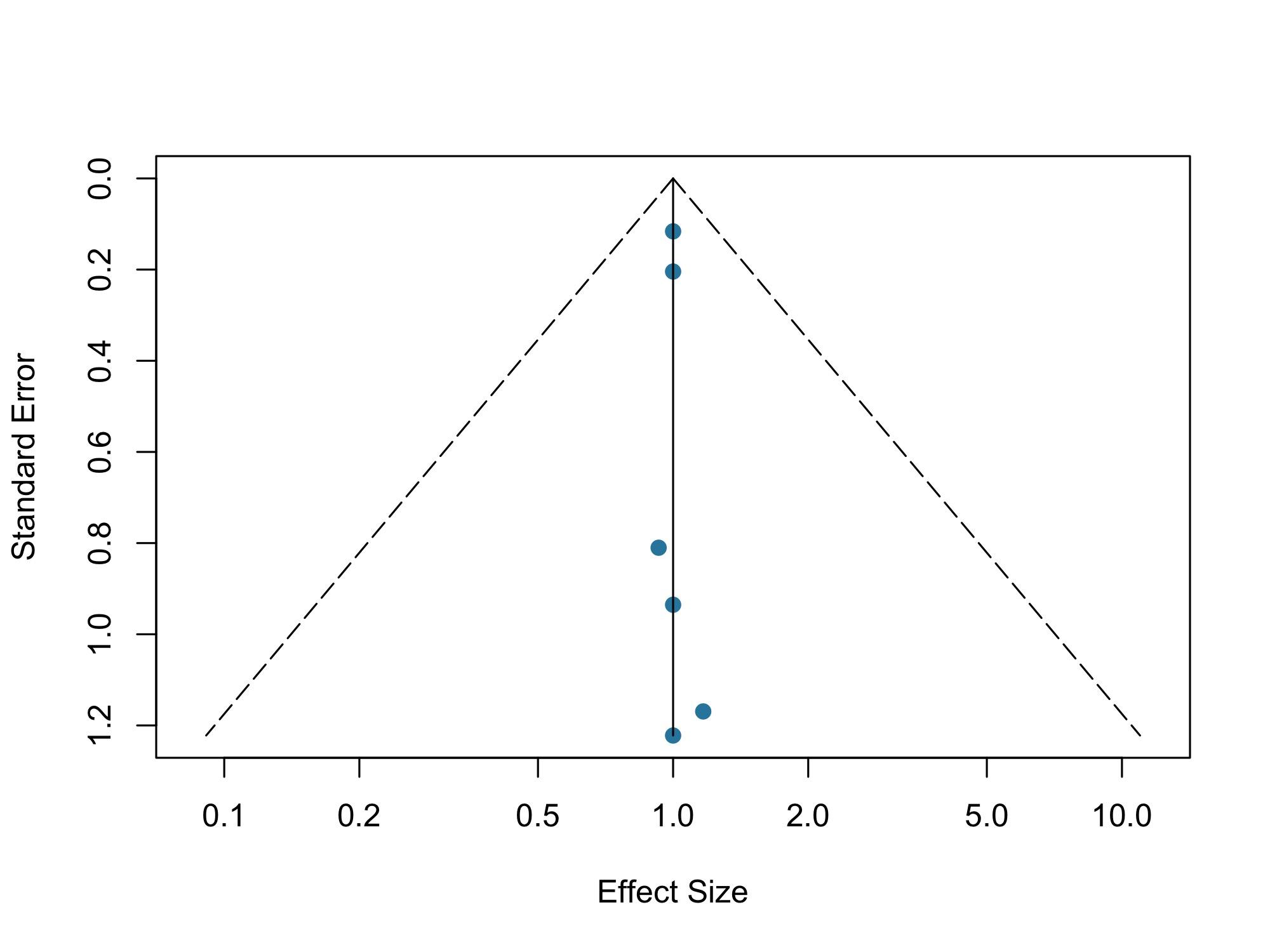
**

S5.14 Publication bias -funnel plot for FPG


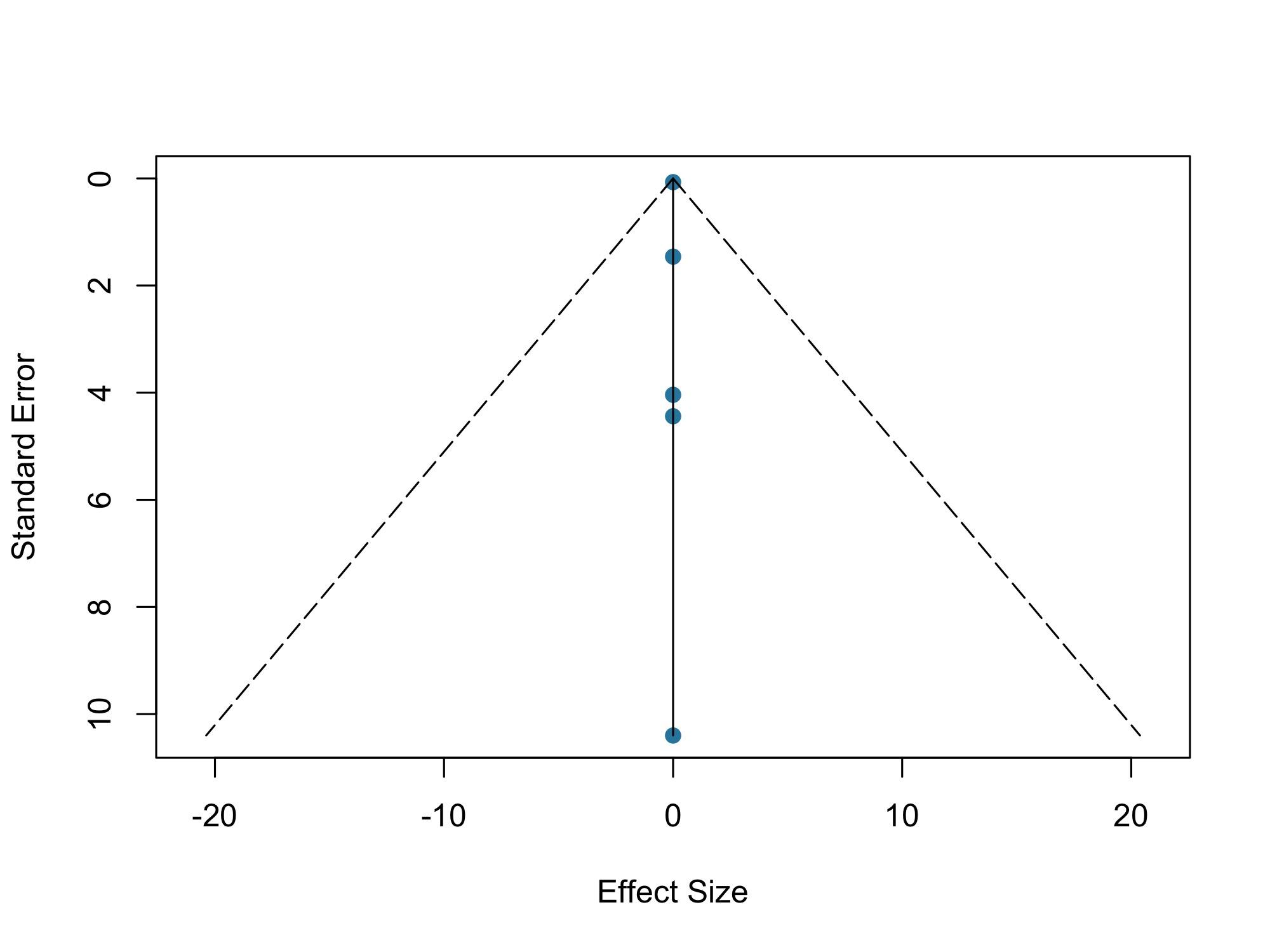


**Supplement 6: League table for each outcome**

S6.1 League table for Height Velocity

|  | Daily rhGH | Lonapegsomatropin 0.24 mg/kg/wk | PEG-rhGH 0.1 mg/kg/wk | PEG-rhGH 0.12 mg/kg/wk | PEG-rhGH 0.2 mg/kg/wk | Somapacitan 0.04/0.16 mg/kg/wk | Somapacitan 0.04mg/kg/wk | Somapacitan 0.08 mg/kg/wk | Somapacitan 0.08/0.16 mg/kg/wk | Somapacitan 0.16 mg/kg/wk | Somatrogon 0.25/0.48/0.66 mg/kg/wk | Somatrogon 0.66 mg/kg/wk | TransCon GH 0.14 mg/kg/wk | TransCon GH 0.21 mg/kg/wk | TransCon GH 0.30 mg/kg/wk | YPEG-rhGH 0.1 mg/kg/wk | YPEG-rhGH 0.12 mg/kg/wk | YPEG-rhGH 0.14 mg/kg/wk |
| --- | --- | --- | --- | --- | --- | --- | --- | --- | --- | --- | --- | --- | --- | --- | --- | --- | --- | --- |
| Daily rhGH | Daily rhGH | -0.90 [-2.55; 0.75] | -2.43 [-4.76; -0.10] | -0.25 [-2.32; 1.82] | 0.30 [-1.38; 1.97] | 1.37 [-0.68; 3.42] | 2.40 [ 0.41; 4.39] | 0.20 [-1.79; 2.19] | 0.27 [-1.74; 2.28] | -0.26 [-1.07; 0.56] | 2.21 [ 0.47; 3.95] | -0.33 [-1.94; 1.28] | 0.28 [-3.12; 3.68] | 1.14 [-1.94; 4.22] | 2.12 [-1.15; 5.39] | -5.02 [-9.10; -0.94] | -1.25 [-4.98; 2.48] | 0.00 [-4.15; 4.15] |
| Lonapegsomatropin 0.24 mg/kg/wk | -0.90 [ -2.55; 0.75] | Lonapegsomatropin 0.24 mg/kg/wk | . | . | . | . | . | . | . | . | . | . | . | . | . | . | . | . |
| PEG-rhGH 0.1 mg/kg/wk | -2.43 [ -4.76; -0.10] | -1.53 [ -4.38; 1.32] | PEG-rhGH 0.1 mg/kg/wk | . | . | . | . | . | . | . | . | . | . | . | . | . | . | . |
| PEG-rhGH 0.12 mg/kg/wk | -0.25 [ -2.32; 1.82] | 0.65 [ -2.00; 3.30] | 2.18 [ -0.94; 5.30] | PEG-rhGH 0.12 mg/kg/wk | . | . | . | . | . | . | . | . | . | . | . | . | . | . |
| PEG-rhGH 0.2 mg/kg/wk | 0.30 [ -1.38; 1.97] | 1.20 [ -1.16; 3.55] | 2.73 [ -0.14; 5.59] | 0.55 [ -2.12; 3.21] | PEG-rhGH 0.2 mg/kg/wk | . | . | . | . | . | . | . | . | . | . | . | . | . |
| Somapacitan 0.04/0.16 mg/kg/wk | 1.37 [ -0.68; 3.42] | 2.27 [ -0.36; 4.90] | 3.80 [ 0.70; 6.90] | 1.62 [ -1.29; 4.53] | 1.07 [ -1.57; 3.72] | Somapacitan 0.04/0.16 mg/kg/wk | . | . | . | . | . | . | . | . | . | . | . | . |
| Somapacitan 0.04mg/kg/wk | 2.40 [ 0.41; 4.39] | 3.30 [ 0.71; 5.89] | 4.83 [ 1.77; 7.89] | 2.65 [ -0.23; 5.53] | 2.10 [ -0.50; 4.71] | 1.03 [ -1.83; 3.89] | Somapacitan 0.04mg/kg/wk | . | . | . | . | . | . | . | . | . | . | . |
| Somapacitan 0.08 mg/kg/wk | 0.20 [ -1.79; 2.19] | 1.10 [ -1.49; 3.69] | 2.63 [ -0.43; 5.69] | 0.45 [ -2.43; 3.33] | -0.10 [ -2.70; 2.51] | -1.17 [ -4.03; 1.69] | -2.20 [ -5.02; 0.62] | Somapacitan 0.08 mg/kg/wk | . | . | . | . | . | . | . | . | . | . |
| Somapacitan 0.08/0.16 mg/kg/wk | 0.27 [ -1.74; 2.28] | 1.17 [ -1.43; 3.77] | 2.70 [ -0.37; 5.77] | 0.52 [ -2.37; 3.41] | -0.03 [ -2.64; 2.59] | -1.10 [ -3.97; 1.77] | -2.13 [ -4.96; 0.70] | 0.07 [ -2.76; 2.90] | Somapacitan 0.08/0.16 mg/kg/wk | . | . | . | . | . | . | . | . | . |
| Somapacitan 0.16 mg/kg/wk | -0.26 [ -1.07; 0.56] | 0.64 [ -1.20; 2.48] | 2.17 [ -0.29; 4.64] | -0.01 [ -2.23; 2.22] | -0.55 [ -2.42; 1.31] | -1.63 [ -3.83; 0.57] | -2.66 [ -4.81; -0.50] | -0.46 [ -2.61; 1.70] | -0.53 [ -2.69; 1.64] | Somapacitan 0.16 mg/kg/wk | . | . | . | . | . | . | . | . |
| Somatrogon 0.25/0.48/0.66 mg/kg/wk | 2.21 [ 0.47; 3.95] | 3.11 [ 0.71; 5.51] | 4.64 [ 1.74; 7.54] | 2.46 [ -0.24; 5.16] | 1.91 [ -0.50; 4.33] | 0.84 [ -1.84; 3.52] | -0.19 [ -2.83; 2.45] | 2.01 [ -0.63; 4.65] | 1.94 [ -0.71; 4.59] | 2.47 [ 0.55; 4.39] | Somatrogon 0.25/0.48/0.66 mg/kg/wk | . | . | . | . | . | . | . |
| Somatrogon 0.66 mg/kg/wk | -0.33 [ -1.94; 1.28] | 0.57 [ -1.74; 2.88] | 2.10 [ -0.73; 4.93] | -0.08 [ -2.71; 2.55] | -0.63 [ -2.95; 1.70] | -1.70 [ -4.31; 0.91] | -2.73 [ -5.30; -0.16] | -0.53 [ -3.10; 2.04] | -0.60 [ -3.18; 1.98] | -0.07 [ -1.88; 1.74] | -2.54 [ -4.91; -0.17] | Somatrogon 0.66 mg/kg/wk | . | . | . | . | . | . |
| TransCon GH 0.14 mg/kg/wk | 0.28 [ -3.12; 3.68] | 1.18 [ -2.60; 4.96] | 2.71 [ -1.41; 6.83] | 0.53 [ -3.45; 4.51] | -0.02 [ -3.80; 3.77] | -1.09 [ -5.05; 2.87] | -2.12 [ -6.06; 1.82] | 0.08 [ -3.86; 4.02] | 0.01 [ -3.93; 3.95] | 0.54 [ -2.95; 4.03] | -1.93 [ -5.74; 1.88] | 0.61 [ -3.15; 4.37] | TransCon GH 0.14 mg/kg/wk | . | . | . | . | . |
| TransCon GH 0.21 mg/kg/wk | 1.14 [ -1.94; 4.22] | 2.04 [ -1.46; 5.54] | 3.57 [ -0.29; 7.43] | 1.39 [ -2.33; 5.11] | 0.84 [ -2.67; 4.36] | -0.23 [ -3.93; 3.47] | -1.26 [ -4.93; 2.41] | 0.94 [ -2.73; 4.61] | 0.87 [ -2.81; 4.55] | 1.40 [ -1.79; 4.59] | -1.07 [ -4.61; 2.47] | 1.47 [ -2.01; 4.95] | 0.86 [ -3.73; 5.45] | TransCon GH 0.21 mg/kg/wk | . | . | . | . |
| TransCon GH 0.30 mg/kg/wk | 2.12 [ -1.15; 5.39] | 3.02 [ -0.65; 6.69] | 4.55 [ 0.53; 8.57] | 2.37 [ -1.50; 6.24] | 1.82 [ -1.85; 5.50] | 0.75 [ -3.11; 4.61] | -0.28 [ -4.11; 3.55] | 1.92 [ -1.91; 5.75] | 1.85 [ -1.99; 5.69] | 2.38 [ -1.00; 5.75] | -0.09 [ -3.80; 3.62] | 2.45 [ -1.20; 6.10] | 1.84 [ -2.88; 6.56] | 0.98 [ -3.52; 5.48] | TransCon GH 0.30 mg/kg/wk | . | . | . |
| YPEG-rhGH 0.1 mg/kg/wk | -5.02 [ -9.10; -0.94] | -4.12 [ -8.52; 0.28] | -2.59 [ -7.28; 2.10] | -4.77 [ -9.34; -0.20] | -5.32 [ -9.72; -0.91] | -6.39 [-10.95; -1.83] | -7.42 [-11.96; -2.88] | -5.22 [ -9.76; -0.68] | -5.29 [ -9.83; -0.75] | -4.76 [ -8.92; -0.61] | -7.23 [-11.66; -2.80] | -4.69 [ -9.07; -0.31] | -5.30 [-10.61; 0.01] | -6.16 [-11.27; -1.05] | -7.14 [-12.37; -1.91] | YPEG-rhGH 0.1 mg/kg/wk | . | . |
| YPEG-rhGH 0.12 mg/kg/wk | -1.25 [ -4.98; 2.48] | -0.35 [ -4.43; 3.73] | 1.18 [ -3.22; 5.58] | -1.00 [ -5.27; 3.27] | -1.55 [ -5.64; 2.55] | -2.62 [ -6.88; 1.64] | -3.65 [ -7.88; 0.58] | -1.45 [ -5.68; 2.78] | -1.52 [ -5.76; 2.72] | -0.99 [ -4.81; 2.83] | -3.46 [ -7.58; 0.66] | -0.92 [ -4.99; 3.15] | -1.53 [ -6.58; 3.52] | -2.39 [ -7.23; 2.45] | -3.37 [ -8.34; 1.60] | 3.77 [ -1.76; 9.30] | YPEG-rhGH 0.12 mg/kg/wk | . |
| YPEG-rhGH 0.14 mg/kg/wk | 0.00 [ -4.15; 4.15] | 0.90 [ -3.56; 5.36] | 2.43 [ -2.33; 7.19] | 0.25 [ -4.39; 4.89] | -0.30 [ -4.77; 4.18] | -1.37 [ -6.00; 3.26] | -2.40 [ -7.00; 2.20] | -0.20 [ -4.80; 4.40] | -0.27 [ -4.88; 4.34] | 0.26 [ -3.97; 4.49] | -2.21 [ -6.71; 2.29] | 0.33 [ -4.12; 4.78] | -0.28 [ -5.64; 5.08] | -1.14 [ -6.31; 4.03] | -2.12 [ -7.40; 3.16] | 5.02 [ -0.80; 10.84] | 1.25 [ -4.33; 6.83] | YPEG-rhGH 0.14 mg/kg/wk |

S6.2 League table for Height SDS

|  | Daily rhGH | Lonapegsomatropin 0.24 mg/kg/wk | PEG-rhGH 0.1 mg/kg/wk | PEG-rhGH 0.2 mg/kg/wk | Somapacitan 0.04/0.16 mg/kg/wk | Somapacitan 0.08/0.16 mg/kg/wk | Somapacitan 0.16 mg/kg/wk | Somatrogon 0.66 mg/kg/wk | TransCon GH 0.14 mg/kg/wk | TransCon GH 0.21 mg/kg/wk | TransCon GH 0.30 mg/kg/wk | YPEG-rhGH 0.1 mg/kg/wk | YPEG-rhGH 0.12 mg/kg/wk | YPEG-rhGH 0.14 mg/kg/wk |
| --- | --- | --- | --- | --- | --- | --- | --- | --- | --- | --- | --- | --- | --- | --- |
| Daily rhGH | Daily rhGH | -0.04 [-0.22; 0.14] | -0.66 [-1.43; 0.11] | -0.03 [-0.47; 0.42] | -0.40 [-1.49; 0.69] | 0.10 [-0.72; 0.92] | -0.02 [-0.15; 0.11] | -0.01 [-0.28; 0.26] | -0.05 [-0.42; 0.32] | 0.07 [-0.23; 0.37] | 0.26 [-0.11; 0.63] | -0.48 [-1.16; 0.20] | -0.14 [-0.64; 0.36] | -0.07 [-0.26; 0.12] |
| Lonapegsomatropin 0.24 mg/kg/wk | -0.04 [-0.22; 0.14] | Lonapegsomatropin 0.24 mg/kg/wk | . | . | . | . | . | . | . | . | . | . | . | . |
| PEG-rhGH 0.1 mg/kg/wk | -0.66 [-1.43; 0.11] | -0.62 [-1.41; 0.17] | PEG-rhGH 0.1 mg/kg/wk | . | . | . | . | . | . | . | . | . | . | . |
| PEG-rhGH 0.2 mg/kg/wk | -0.03 [-0.47; 0.42] | 0.01 [-0.47; 0.49] | 0.63 [-0.25; 1.52] | PEG-rhGH 0.2 mg/kg/wk | . | . | . | . | . | . | . | . | . | . |
| Somapacitan 0.04/0.16 mg/kg/wk | -0.40 [-1.49; 0.69] | -0.36 [-1.47; 0.75] | 0.26 [-1.08; 1.60] | -0.37 [-1.55; 0.81] | Somapacitan 0.04/0.16 mg/kg/wk | . | . | . | . | . | . | . | . | . |
| Somapacitan 0.08/0.16 mg/kg/wk | 0.10 [-0.72; 0.92] | 0.14 [-0.71; 0.98] | 0.76 [-0.37; 1.89] | 0.13 [-0.81; 1.06] | 0.50 [-0.87; 1.87] | Somapacitan 0.08/0.16 mg/kg/wk | . | . | . | . | . | . | . | . |
| Somapacitan 0.16 mg/kg/wk | -0.02 [-0.15; 0.11] | 0.01 [-0.21; 0.24] | 0.64 [-0.14; 1.41] | 0.00 [-0.46; 0.46] | 0.38 [-0.72; 1.48] | -0.12 [-0.96; 0.71] | Somapacitan 0.16 mg/kg/wk | . | . | . | . | . | . | . |
| Somatrogon 0.66 mg/kg/wk | -0.01 [-0.28; 0.26] | 0.03 [-0.30; 0.35] | 0.65 [-0.16; 1.46] | 0.02 [-0.50; 0.53] | 0.39 [-0.74; 1.52] | -0.11 [-0.98; 0.76] | 0.01 [-0.28; 0.31] | Somatrogon 0.66 mg/kg/wk | . | . | . | . | . | . |
| TransCon GH 0.14 mg/kg/wk | -0.05 [-0.42; 0.32] | -0.01 [-0.42; 0.40] | 0.61 [-0.24; 1.46] | -0.02 [-0.60; 0.55] | 0.35 [-0.80; 1.50] | -0.15 [-1.05; 0.75] | -0.03 [-0.42; 0.36] | -0.04 [-0.50; 0.42] | TransCon GH 0.14 mg/kg/wk | . | . | . | . | . |
| TransCon GH 0.21 mg/kg/wk | 0.07 [-0.23; 0.37] | 0.11 [-0.25; 0.46] | 0.73 [-0.09; 1.55] | 0.10 [-0.44; 0.63] | 0.47 [-0.66; 1.60] | -0.03 [-0.91; 0.85] | 0.09 [-0.24; 0.42] | 0.08 [-0.32; 0.48] | 0.12 [-0.36; 0.60] | TransCon GH 0.21 mg/kg/wk | . | . | . | . |
| TransCon GH 0.30 mg/kg/wk | 0.26 [-0.11; 0.63] | 0.30 [-0.12; 0.71] | 0.92 [ 0.07; 1.77] | 0.29 [-0.29; 0.86] | 0.66 [-0.49; 1.81] | 0.16 [-0.74; 1.06] | 0.28 [-0.11; 0.68] | 0.27 [-0.19; 0.73] | 0.31 [-0.21; 0.83] | 0.19 [-0.29; 0.67] | TransCon GH 0.30 mg/kg/wk | . | . | . |
| YPEG-rhGH 0.1 mg/kg/wk | -0.48 [-1.16; 0.20] | -0.44 [-1.14; 0.26] | 0.18 [-0.84; 1.20] | -0.45 [-1.26; 0.35] | -0.08 [-1.37; 1.21] | -0.58 [-1.65; 0.49] | -0.46 [-1.14; 0.23] | -0.47 [-1.20; 0.26] | -0.43 [-1.20; 0.34] | -0.55 [-1.29; 0.19] | -0.74 [-1.51; 0.03] | YPEG-rhGH 0.1 mg/kg/wk | . | . |
| YPEG-rhGH 0.12 mg/kg/wk | -0.14 [-0.64; 0.36] | -0.10 [-0.64; 0.43] | 0.52 [-0.40; 1.44] | -0.11 [-0.79; 0.56] | 0.26 [-0.94; 1.46] | -0.24 [-1.21; 0.73] | -0.12 [-0.64; 0.41] | -0.13 [-0.70; 0.44] | -0.09 [-0.71; 0.53] | -0.21 [-0.80; 0.38] | -0.40 [-1.03; 0.23] | 0.34 [-0.50; 1.18] | YPEG-rhGH 0.12 mg/kg/wk | . |
|  | Daily rhGH | Lonapegsomatropin 0.24 mg/kg/wk | PEG-rhGH 0.1 mg/kg/wk | PEG-rhGH 0.2 mg/kg/wk | Somapacitan 0.04/0.16 mg/kg/wk | Somapacitan 0.08/0.16 mg/kg/wk | Somapacitan 0.16 mg/kg/wk | Somatrogon 0.66 mg/kg/wk | TransCon GH 0.14 mg/kg/wk | TransCon GH 0.21 mg/kg/wk | TransCon GH 0.30 mg/kg/wk | YPEG-rhGH 0.1 mg/kg/wk | YPEG-rhGH 0.12 mg/kg/wk | YPEG-rhGH 0.14 mg/kg/wk |

S6.3 League table for Treatment discontinuation

|  | Daily rhGH | Lonapegsomatropin 0.24 mg/kg/wk | PEG-rhGH 0.1 mg/kg/wk | PEG-rhGH 0.2 mg/kg/wk | Somapacitan 0.16 mg/kg/wk | Somatrogon 0.66 mg/kg/wk | TransCon GH 0.14 mg/kg/wk | TransCon GH 0.21 mg/kg/wk | TransCon GH 0.30 mg/kg/wk | YPEG-rhGH 0.1 mg/kg/wk | YPEG-rhGH 0.12 mg/kg/wk | YPEG-rhGH 0.14 mg/kg/wk |
| --- | --- | --- | --- | --- | --- | --- | --- | --- | --- | --- | --- | --- |
| Daily rhGH | Daily rhGH | 1.87 [0.10; 35.54] | 1.98 [0.10; 37.93] | 1.98 [0.10; 37.93] | 1.94 [0.10; 36.92] | 0.95 [0.05; 18.08] | 1.08 [0.06; 18.84] | 1.08 [0.06; 18.84] | 1.08 [0.06; 18.84] | 5.17 [0.41; 64.80] | 5.17 [0.41; 64.80] | 1.87 [0.39; 8.88] |
| Lonapegsomatropin 0.24 mg/kg/wk | 1.87 [0.10; 35.54] | Lonapegsomatropin 0.24 mg/kg/wk | . | . | . | . | . | . | . | . | . | . |
| PEG-rhGH 0.1 mg/kg/wk | 1.98 [0.10; 37.93] | 1.06 [0.02; 68.25] | PEG-rhGH 0.1 mg/kg/wk | . | . | . | . | . | . | . | . | . |
| PEG-rhGH 0.2 mg/kg/wk | 1.98 [0.10; 37.93] | 1.06 [0.02; 68.25] | 1.00 [0.02; 64.97] | PEG-rhGH 0.2 mg/kg/wk | . | . | . | . | . | . | . | . |
| Somapacitan 0.16 mg/kg/wk | 1.94 [0.10; 36.92] | 1.04 [0.02; 66.54] | 0.98 [0.02; 63.35] | 0.98 [0.02; 63.35] | Somapacitan 0.16 mg/kg/wk | . | . | . | . | . | . | . |
| Somatrogon 0.66 mg/kg/wk | 0.95 [0.05; 18.08] | 0.51 [0.01; 32.56] | 0.48 [0.01; 30.99] | 0.48 [0.01; 30.99] | 0.49 [0.01; 31.52] | Somatrogon 0.66 mg/kg/wk | . | . | . | . | . | . |
| TransCon GH 0.14 mg/kg/wk | 1.08 [0.06; 18.84] | 0.57 [0.01; 34.82] | 0.54 [0.01; 33.15] | 0.54 [0.01; 33.15] | 0.55 [0.01; 33.71] | 1.14 [0.02; 69.18] | TransCon GH 0.14 mg/kg/wk | . | . | . | . | . |
| TransCon GH 0.21 mg/kg/wk | 1.08 [0.06; 18.84] | 0.57 [0.01; 34.82] | 0.54 [0.01; 33.15] | 0.54 [0.01; 33.15] | 0.55 [0.01; 33.71] | 1.14 [0.02; 69.18] | 1.00 [0.02; 57.26] | TransCon GH 0.21 mg/kg/wk | . | . | . | . |
| TransCon GH 0.30 mg/kg/wk | 1.08 [0.06; 18.84] | 0.57 [0.01; 34.82] | 0.54 [0.01; 33.15] | 0.54 [0.01; 33.15] | 0.55 [0.01; 33.71] | 1.14 [0.02; 69.18] | 1.00 [0.02; 57.26] | 1.00 [0.02; 57.26] | TransCon GH 0.30 mg/kg/wk | . | . | . |
| YPEG-rhGH 0.1 mg/kg/wk | 5.17 [0.41; 64.80] | 2.76 [0.06; 133.40] | 2.61 [0.05; 127.06] | 2.61 [0.05; 127.06] | 2.66 [0.05; 129.18] | 5.45 [0.11; 265.15] | 4.80 [0.11; 218.66] | 4.80 [0.11; 218.66] | 4.80 [0.11; 218.66] | YPEG-rhGH 0.1 mg/kg/wk | . | . |
| YPEG-rhGH 0.12 mg/kg/wk | 5.17 [0.41; 64.80] | 2.76 [0.06; 133.40] | 2.61 [0.05; 127.06] | 2.61 [0.05; 127.06] | 2.66 [0.05; 129.18] | 5.45 [0.11; 265.15] | 4.80 [0.11; 218.66] | 4.80 [0.11; 218.66] | 4.80 [0.11; 218.66] | 1.00 [0.03; 35.76] | YPEG-rhGH 0.12 mg/kg/wk | . |
| YPEG-rhGH 0.14 mg/kg/wk | 1.87 [0.39; 8.88] | 1.00 [0.04; 27.85] | 0.94 [0.03; 26.55] | 0.94 [0.03; 26.55] | 0.96 [0.03; 26.98] | 1.97 [0.07; 55.39] | 1.74 [0.07; 45.18] | 1.74 [0.07; 45.18] | 1.74 [0.07; 45.18] | 0.36 [0.02; 7.06] | 0.36 [0.02; 7.06] | YPEG-rhGH 0.14 mg/kg/wk |

S6.4 League table for Injection site erythema

|  | Daily rhGH | PEG-rhGH 0.1 mg/kg/wk | PEG-rhGH 0.2 mg/kg/wk | Somapacitan 0.16 mg/kg/wk | Somatrogon 0.66 mg/kg/wk | TransCon GH 0.14 mg/kg/wk | TransCon GH 0.21 mg/kg/wk | TransCon GH 0.30 mg/kg/wk |
| --- | --- | --- | --- | --- | --- | --- | --- | --- |
| Daily rhGH | Daily rhGH | 1.98 [0.28; 13.89] | 1.98 [0.28; 13.89] | 3.37 [0.82; 13.89] | 0.09 [0.01; 0.73] | 0.79 [0.37; 1.69] | 1.08 [0.46; 2.50] | 1.08 [0.46; 2.50] |
| PEG-rhGH 0.1 mg/kg/wk | 1.98 [0.28; 13.89] | PEG-rhGH 0.1 mg/kg/wk | . | . | . | . | . | . |
| PEG-rhGH 0.2 mg/kg/wk | 1.98 [0.28; 13.89] | 1.00 [0.06; 15.70] | PEG-rhGH 0.2 mg/kg/wk | . | . | . | . | . |
| Somapacitan 0.16 mg/kg/wk | 3.37 [0.82; 13.89] | 1.70 [0.15; 18.89] | 1.70 [0.15; 18.89] | Somapacitan 0.16 mg/kg/wk | . | . | . | . |
| Somatrogon 0.66 mg/kg/wk | 0.09 [0.01; 0.73] | 0.05 [0.00; 0.80] | 0.05 [0.00; 0.80] | 0.03 [0.00; 0.34] | Somatrogon 0.66 mg/kg/wk | . | . | . |
| TransCon GH 0.14 mg/kg/wk | 0.79 [0.37; 1.69] | 0.40 [0.05; 3.22] | 0.40 [0.05; 3.22] | 0.23 [0.05; 1.17] | 8.35 [0.95; 73.47] | TransCon GH 0.14 mg/kg/wk | . | . |
| TransCon GH 0.21 mg/kg/wk | 1.08 [0.46; 2.50] | 0.54 [0.07; 4.53] | 0.54 [0.07; 4.53] | 0.32 [0.06; 1.66] | 11.36 [1.25; 103.19] | 1.36 [0.44; 4.23] | TransCon GH 0.21 mg/kg/wk | . |
| TransCon GH 0.30 mg/kg/wk | 1.08 [0.46; 2.50] | 0.54 [0.07; 4.53] | 0.54 [0.07; 4.53] | 0.32 [0.06; 1.66] | 11.36 [1.25; 103.19] | 1.36 [0.44; 4.23] | 1.00 [0.30; 3.29] | TransCon GH 0.30 mg/kg/wk |

S6.5 League table for Influenza

|  | Daily rhGH | Lonapegsomatropin 0.24 mg/kg/wk | Somapacitan 0.08 mg/kg/wk | Somapacitan 0.16 mg/kg/wk | Somatrogon 0.25 mg/kg/wk | Somatrogon 0.48 mg/kg/wk | Somatrogon 0.66 mg/kg/wk |
| --- | --- | --- | --- | --- | --- | --- | --- |
| Daily rhGH | Daily rhGH | 1.07 [0.34; 3.42] | 1.07 [0.17; 6.61] | 2.00 [0.20; 19.62] | 0.90 [0.34; 2.38] | 0.90 [0.34; 2.38] | 0.90 [0.34; 2.38] |
| Lonapegsomatropin 0.24 mg/kg/wk | 1.07 [0.34; 3.42] | Lonapegsomatropin 0.24 mg/kg/wk | . | . | . | . | . |
| Somapacitan 0.08 mg/kg/wk | 1.07 [0.17; 6.61] | 1.00 [0.12; 8.66] | Somapacitan 0.08 mg/kg/wk | . | . | . | . |
| Somapacitan 0.16 mg/kg/wk | 2.00 [0.20; 19.62] | 1.87 [0.14; 24.18] | 1.87 [0.10; 34.60] | Somapacitan 0.16 mg/kg/wk | . | . | . |
| Somatrogon 0.25 mg/kg/wk | 0.90 [0.34; 2.38] | 0.84 [0.19; 3.82] | 0.84 [0.11; 6.62] | 0.45 [0.04; 5.40] | Somatrogon 0.25 mg/kg/wk | . | . |
| Somatrogon 0.48 mg/kg/wk | 0.90 [0.34; 2.38] | 0.84 [0.19; 3.82] | 0.84 [0.11; 6.62] | 0.45 [0.04; 5.40] | 1.00 [0.26; 3.91] | Somatrogon 0.48 mg/kg/wk | . |
| Somatrogon 0.66 mg/kg/wk | 0.90 [0.34; 2.38] | 0.84 [0.19; 3.82] | 0.84 [0.11; 6.62] | 0.45 [0.04; 5.40] | 1.00 [0.26; 3.91] | 1.00 [0.26; 3.91] | Somatrogon 0.66 mg/kg/wk |

S6.6 League table for Headache

|  | Daily rhGH | Lonapegsomatropin 0.24 mg/kg/wk | PEG-rhGH 0.1 mg/kg/wk | PEG-rhGH 0.2 mg/kg/wk | Somatrogon 0.66 mg/kg/wk | TransCon GH 0.14 mg/kg/wk | TransCon GH 0.30 mg/kg/wk |
| --- | --- | --- | --- | --- | --- | --- | --- |
| Daily rhGH | Daily rhGH | 1.01 [0.43; 2.39] | 0.50 [0.06; 4.40] | 0.50 [0.06; 4.40] | 1.32 [0.76; 2.27] | 0.46 [0.05; 4.46] | 1.08 [0.07; 15.50] |
| Lonapegsomatropin 0.24 mg/kg/wk | 1.01 [0.43; 2.39] | Lonapegsomatropin 0.24 mg/kg/wk | . | . | . | . | . |
| PEG-rhGH 0.1 mg/kg/wk | 0.50 [0.06; 4.40] | 0.49 [0.05; 5.13] | PEG-rhGH 0.1 mg/kg/wk | . | . | . | . |
| PEG-rhGH 0.2 mg/kg/wk | 0.50 [0.06; 4.40] | 0.49 [0.05; 5.13] | 1.00 [0.05; 21.83] | PEG-rhGH 0.2 mg/kg/wk | . | . | . |
| Somatrogon 0.66 mg/kg/wk | 1.32 [0.76; 2.27] | 1.30 [0.47; 3.61] | 2.65 [0.28; 25.08] | 2.65 [0.28; 25.08] | Somatrogon 0.66 mg/kg/wk | . | . |
| TransCon GH 0.14 mg/kg/wk | 0.46 [0.05; 4.46] | 0.46 [0.04; 5.17] | 0.93 [0.04; 21.60] | 0.93 [0.04; 21.60] | 0.35 [0.03; 3.62] | TransCon GH 0.14 mg/kg/wk | . |
| TransCon GH 0.30 mg/kg/wk | 1.08 [0.07; 15.50] | 1.07 [0.06; 17.58] | 2.17 [0.07; 67.92] | 2.17 [0.07; 67.92] | 0.82 [0.05; 12.45] | 2.33 [0.07; 77.38] | TransCon GH 0.30 mg/kg/wk |

S6.7 League table for Fever

|  | Daily rhGH | Lonapegsomatropin 0.24 mg/kg/wk | Somatrogon 0.25/0.48/0.66 mg/kg/wk | Somatrogon 0.66 mg/kg/wk | TransCon GH 0.30 mg/kg/wk | YPEG-rhGH 0.14 mg/kg/wk |
| --- | --- | --- | --- | --- | --- | --- |
| Daily rhGH | Daily rhGH | 0.59 [0.23; 1.52] | 0.75 [0.19; 2.97] | 0.84 [0.45; 1.57] | 3.23 [0.38; 27.28] | 1.38 [0.93; 2.04] |
| Lonapegsomatropin 0.24 mg/kg/wk | 0.59 [0.23; 1.52] | Lonapegsomatropin 0.24 mg/kg/wk | . | . | . | . |
| Somatrogon 0.25/0.48/0.66 mg/kg/wk | 0.75 [0.19; 2.97] | 1.28 [0.24; 6.81] | Somatrogon 0.25/0.48/0.66 mg/kg/wk | . | . | . |
| Somatrogon 0.66 mg/kg/wk | 0.84 [0.45; 1.57] | 1.44 [0.46; 4.47] | 1.12 [0.25; 5.08] | Somatrogon 0.66 mg/kg/wk | . | . |
| TransCon GH 0.30 mg/kg/wk | 3.23 [0.38; 27.28] | 5.51 [0.53; 56.99] | 4.31 [0.34; 54.54] | 3.83 [0.42; 35.37] | TransCon GH 0.30 mg/kg/wk | . |
| YPEG-rhGH 0.14 mg/kg/wk | 1.38 [0.93; 2.04] | 2.36 [0.84; 6.58] | 1.84 [0.44; 7.69] | 1.64 [0.79; 3.41] | 0.43 [0.05; 3.74] | YPEG-rhGH 0.14 mg/kg/wk |

S6.8 League table for hypothyroidism

|  | Daily rhGH | PEG-rhGH 0.1 mg/kg/wk | PEG-rhGH 0.2 mg/kg/wk | Somatrogon 0.25 mg/kg/wk | Somatrogon 0.48 mg/kg/wk | Somatrogon 0.66 mg/kg/wk | YPEG-rhGH 0.12 mg/kg/wk | YPEG-rhGH 0.14 mg/kg/wk |
| --- | --- | --- | --- | --- | --- | --- | --- | --- |
| Daily rhGH | Daily rhGH | 2.24 [0.24; 20.88] | 2.24 [0.24; 20.88] | 0.33 [0.04; 2.96] | 0.33 [0.04; 2.96] | 0.39 [0.12; 1.20] | 5.17 [0.51; 51.85] | 5.17 [0.51; 51.85] |
| PEG-rhGH 0.1 mg/kg/wk | 2.24 [0.24; 20.88] | PEG-rhGH 0.1 mg/kg/wk | . | . | . | . | . | . |
| PEG-rhGH 0.2 mg/kg/wk | 2.24 [0.24; 20.88] | 1.00 [0.04; 23.57] | PEG-rhGH 0.2 mg/kg/wk | . | . | . | . | . |
| Somatrogon 0.25 mg/kg/wk | 0.33 [0.04; 2.96] | 0.15 [0.01; 3.39] | 0.15 [0.01; 3.39] | Somatrogon 0.25 mg/kg/wk | . | . | . | . |
| Somatrogon 0.48 mg/kg/wk | 0.33 [0.04; 2.96] | 0.15 [0.01; 3.39] | 0.15 [0.01; 3.39] | 1.00 [0.05; 21.97] | Somatrogon 0.48 mg/kg/wk | . | . | . |
| Somatrogon 0.66 mg/kg/wk | 0.39 [0.12; 1.20] | 0.17 [0.01; 2.11] | 0.17 [0.01; 2.11] | 1.16 [0.10; 13.54] | 1.16 [0.10; 13.54] | Somatrogon 0.66 mg/kg/wk | . | . |
| YPEG-rhGH 0.12 mg/kg/wk | 5.17 [0.51; 51.85] | 2.31 [0.09; 57.33] | 2.31 [0.09; 57.33] | 15.50 [0.65; 371.46] | 15.50 [0.65; 371.46] | 13.42 [1.03; 175.26] | YPEG-rhGH 0.12 mg/kg/wk | . |
| YPEG-rhGH 0.14 mg/kg/wk | 5.17 [0.51; 51.85] | 2.31 [0.09; 57.33] | 2.31 [0.09; 57.33] | 15.50 [0.65; 371.46] | 15.50 [0.65; 371.46] | 13.42 [1.03; 175.26] | 1.00 [0.04; 26.08] | YPEG-rhGH 0.14 mg/kg/wk |

S6.9 League table for Injection site pain

|  | Daily rhGH | Somapacitan 0.16 mg/kg/wk | Somatrogon 0.25 mg/kg/wk | Somatrogon 0.48 mg/kg/wk | Somatrogon 0.66 mg/kg/wk | TransCon GH 0.14 mg/kg/wk | TransCon GH 0.21 mg/kg/wk | TransCon GH 0.30 mg/kg/wk | YPEG-rhGH 0.1 mg/kg/wk | YPEG-rhGH 0.12 mg/kg/wk | YPEG-rhGH 0.14 mg/kg/wk |
| --- | --- | --- | --- | --- | --- | --- | --- | --- | --- | --- | --- |
| Daily rhGH | Daily rhGH | 1.12 [0.32; 3.96] | 0.19 [0.06; 0.63] | 0.19 [0.06; 0.63] | 0.48 [0.27; 0.86] | 1.11 [0.39; 3.15] | 1.29 [0.44; 3.75] | 1.08 [0.39; 2.94] | 0.86 [0.09; 8.01] | 0.86 [0.09; 8.01] | 0.86 [0.09; 8.01] |
| Somapacitan 0.16 mg/kg/wk | 1.12 [0.32; 3.96] | Somapacitan 0.16 mg/kg/wk | . | . | . | . | . | . | . | . | . |
| Somatrogon 0.25 mg/kg/wk | 0.19 [0.06; 0.63] | 0.17 [0.03; 0.96] | Somatrogon 0.25 mg/kg/wk | . | . | . | . | . | . | . | . |
| Somatrogon 0.48 mg/kg/wk | 0.19 [0.06; 0.63] | 0.17 [0.03; 0.96] | 1.00 [0.18; 5.55] | Somatrogon 0.48 mg/kg/wk | . | . | . | . | . | . | . |
| Somatrogon 0.66 mg/kg/wk | 0.48 [0.27; 0.86] | 0.43 [0.11; 1.71] | 2.56 [0.66; 9.83] | 2.56 [0.66; 9.83] | Somatrogon 0.66 mg/kg/wk | . | . | . | . | . | . |
| TransCon GH 0.14 mg/kg/wk | 1.11 [0.39; 3.15] | 0.99 [0.19; 5.07] | 5.91 [1.19; 29.27] | 5.91 [1.19; 29.27] | 2.31 [0.70; 7.66] | TransCon GH 0.14 mg/kg/wk | . | . | . | . | . |
| TransCon GH 0.21 mg/kg/wk | 1.29 [0.44; 3.75] | 1.15 [0.22; 5.99] | 6.89 [1.37; 34.64] | 6.89 [1.37; 34.64] | 2.70 [0.80; 9.11] | 1.17 [0.26; 5.19] | TransCon GH 0.21 mg/kg/wk | . | . | . | . |
| TransCon GH 0.30 mg/kg/wk | 1.08 [0.39; 2.94] | 0.96 [0.19; 4.80] | 5.74 [1.19; 27.73] | 5.74 [1.19; 27.73] | 2.25 [0.70; 7.20] | 0.97 [0.23; 4.14] | 0.83 [0.19; 3.61] | TransCon GH 0.30 mg/kg/wk | . | . | . |
| YPEG-rhGH 0.1 mg/kg/wk | 0.86 [0.09; 8.01] | 0.77 [0.06; 9.94] | 4.59 [0.36; 58.17] | 4.59 [0.36; 58.17] | 1.80 [0.18; 18.05] | 0.78 [0.07; 9.13] | 0.67 [0.06; 7.90] | 0.80 [0.07; 9.23] | YPEG-rhGH 0.1 mg/kg/wk | . | . |
| YPEG-rhGH 0.12 mg/kg/wk | 0.86 [0.09; 8.01] | 0.77 [0.06; 9.94] | 4.59 [0.36; 58.17] | 4.59 [0.36; 58.17] | 1.80 [0.18; 18.05] | 0.78 [0.07; 9.13] | 0.67 [0.06; 7.90] | 0.80 [0.07; 9.23] | 1.00 [0.04; 23.45] | YPEG-rhGH 0.12 mg/kg/wk | . |
| YPEG-rhGH 0.14 mg/kg/wk | 0.86 [0.09; 8.01] | 0.77 [0.06; 9.94] | 4.59 [0.36; 58.17] | 4.59 [0.36; 58.17] | 1.80 [0.18; 18.05] | 0.78 [0.07; 9.13] | 0.67 [0.06; 7.90] | 0.80 [0.07; 9.23] | 1.00 [0.04; 23.45] | 1.00 [0.04; 23.45] | YPEG-rhGH 0.14 mg/kg/wk |

S6.10 League table for IGF-1

|  | Daily rhGH | Lonapegsomatropin 0.24 mg/kg/wk | PEG-rhGH 0.1 mg/kg/wk | PEG-rhGH 0.2 mg/kg/wk | Somapacitan 0.04/0.16 mg/kg/wk | Somapacitan 0.04mg/kg/wk | Somapacitan 0.08 mg/kg/wk | Somapacitan 0.08/0.16 mg/kg/wk | Somapacitan 0.16 mg/kg/wk | Somapacitan 0.16/0.16 mg/kg/wk | Somatrogon 0.25/0.48/0.66 mg/kg/wk | YPEG-rhGH 0.14 mg/kg/wk |
| --- | --- | --- | --- | --- | --- | --- | --- | --- | --- | --- | --- | --- |
| Daily rhGH | Daily rhGH | -0.74 [-1.12; -0.36] | 0.41 [-0.18; 1.00] | -0.11 [-0.75; 0.53] | -0.33 [-1.22; 0.56] | 1.65 [ 0.42; 2.88] | -0.08 [-1.06; 0.90] | -0.27 [-1.23; 0.69] | -0.29 [-0.65; 0.07] | 0.33 [-0.43; 1.09] | 2.24 [ 1.69; 2.79] | -0.29 [-0.64; 0.06] |
| Lonapegsomatropin 0.24 mg/kg/wk | -0.74 [-1.12; -0.36] | Lonapegsomatropin 0.24 mg/kg/wk | . | . | . | . | . | . | . | . | . | . |
| PEG-rhGH 0.1 mg/kg/wk | 0.41 [-0.18; 1.00] | 1.15 [ 0.45; 1.85] | PEG-rhGH 0.1 mg/kg/wk | . | . | . | . | . | . | . | . | . |
| PEG-rhGH 0.2 mg/kg/wk | -0.11 [-0.75; 0.53] | 0.63 [-0.11; 1.37] | -0.52 [-1.38; 0.34] | PEG-rhGH 0.2 mg/kg/wk | . | . | . | . | . | . | . | . |
| Somapacitan 0.04/0.16 mg/kg/wk | -0.33 [-1.22; 0.56] | 0.41 [-0.56; 1.38] | -0.74 [-1.81; 0.33] | -0.22 [-1.31; 0.87] | Somapacitan 0.04/0.16 mg/kg/wk | . | . | . | . | . | . | . |
| Somapacitan 0.04mg/kg/wk | 1.65 [ 0.42; 2.88] | 2.39 [ 1.10; 3.68] | 1.24 [-0.12; 2.60] | 1.76 [ 0.38; 3.14] | 1.98 [ 0.46; 3.50] | Somapacitan 0.04mg/kg/wk | . | . | . | . | . | . |
| Somapacitan 0.08 mg/kg/wk | -0.08 [-1.06; 0.90] | 0.66 [-0.40; 1.72] | -0.49 [-1.63; 0.65] | 0.03 [-1.14; 1.20] | 0.25 [-1.08; 1.58] | -1.73 [-3.30; -0.16] | Somapacitan 0.08 mg/kg/wk | . | . | . | . | . |
| Somapacitan 0.08/0.16 mg/kg/wk | -0.27 [-1.23; 0.69] | 0.47 [-0.57; 1.51] | -0.68 [-1.81; 0.45] | -0.16 [-1.32; 1.00] | 0.06 [-1.25; 1.37] | -1.92 [-3.48; -0.36] | -0.19 [-1.57; 1.19] | Somapacitan 0.08/0.16 mg/kg/wk | . | . | . | . |
| Somapacitan 0.16 mg/kg/wk | -0.29 [-0.65; 0.07] | 0.45 [-0.07; 0.98] | -0.70 [-1.38; -0.01] | -0.18 [-0.91; 0.55] | 0.04 [-0.92; 1.00] | -1.94 [-3.22; -0.66] | -0.21 [-1.25; 0.84] | -0.02 [-1.05; 1.01] | Somapacitan 0.16 mg/kg/wk | . | . | . |
| Somapacitan 0.16/0.16 mg/kg/wk | 0.33 [-0.43; 1.09] | 1.07 [ 0.22; 1.92] | -0.08 [-1.04; 0.88] | 0.44 [-0.55; 1.43] | 0.66 [-0.51; 1.83] | -1.32 [-2.77; 0.13] | 0.41 [-0.83; 1.65] | 0.60 [-0.63; 1.83] | 0.62 [-0.23; 1.46] | Somapacitan 0.16/0.16 mg/kg/wk | . | . |
| Somatrogon 0.25/0.48/0.66 mg/kg/wk | 2.24 [ 1.69; 2.79] | 2.98 [ 2.31; 3.65] | 1.83 [ 1.03; 2.63] | 2.35 [ 1.51; 3.19] | 2.57 [ 1.52; 3.62] | 0.59 [-0.76; 1.94] | 2.32 [ 1.19; 3.45] | 2.51 [ 1.40; 3.62] | 2.53 [ 1.87; 3.19] | 1.91 [ 0.97; 2.85] | Somatrogon 0.25/0.48/0.66 mg/kg/wk | . |
| YPEG-rhGH 0.14 mg/kg/wk | -0.29 [-0.64; 0.06] | 0.45 [-0.07; 0.97] | -0.70 [-1.38; -0.02] | -0.18 [-0.91; 0.55] | 0.04 [-0.92; 1.00] | -1.94 [-3.22; -0.66] | -0.21 [-1.25; 0.83] | -0.02 [-1.05; 1.01] | -0.00 [-0.50; 0.50] | -0.62 [-1.46; 0.22] | -2.53 [-3.18; -1.88] | YPEG-rhGH 0.14 mg/kg/wk |

S6.11 League table for HbA1C%

|  | Daily rhGH | Lonapegsomatropin 0.24 mg/kg/wk | PEG-rhGH 0.2 mg/kg/wk | TransCon GH 0.14 mg/kg/wk | TransCon GH 0.21 mg/kg/wk | TransCon GH 0.30 mg/kg/wk |
| --- | --- | --- | --- | --- | --- | --- |
| Daily rhGH | Daily rhGH | -0.08 [-0.18; 0.02] | -0.04 [-0.14; 0.06] | 0.00 [-0.24; 0.24] | 0.00 [-0.22; 0.22] | 0.10 [-0.12; 0.32] |
| Lonapegsomatropin 0.24 mg/kg/wk | -0.08 [-0.18; 0.02] | Lonapegsomatropin 0.24 mg/kg/wk | . | . | . | . |
| PEG-rhGH 0.2 mg/kg/wk | -0.04 [-0.14; 0.06] | 0.04 [-0.10; 0.18] | PEG-rhGH 0.2 mg/kg/wk | . | . | . |
| TransCon GH 0.14 mg/kg/wk | -0.00 [-0.24; 0.24] | 0.08 [-0.17; 0.33] | 0.04 [-0.21; 0.29] | TransCon GH 0.14 mg/kg/wk | . | . |
| TransCon GH 0.21 mg/kg/wk | -0.00 [-0.22; 0.22] | 0.08 [-0.16; 0.32] | 0.04 [-0.20; 0.28] | 0.00 [-0.32; 0.32] | TransCon GH 0.21 mg/kg/wk | . |
| TransCon GH 0.30 mg/kg/wk | 0.10 [-0.12; 0.32] | 0.18 [-0.06; 0.42] | 0.14 [-0.10; 0.38] | 0.10 [-0.22; 0.42] | 0.10 [-0.20; 0.40] | TransCon GH 0.30 mg/kg/wk |

S6.12 League table for cough

|  | Daily rhGH | Somapacitan 0.04mg/kg/wk | Somatrogon 0.66 mg/kg/wk | YPEG-rhGH 0.14 mg/kg/wk |
| --- | --- | --- | --- | --- |
| Daily rhGH | Daily rhGH | 2.27 [0.21; 24.93] | 0.95 [0.39; 2.30] | 1.32 [0.83; 2.09] |
| Somapacitan 0.04mg/kg/wk | 2.27 [0.21; 24.93] | Somapacitan 0.04mg/kg/wk | . | . |
| Somatrogon 0.66 mg/kg/wk | 0.95 [0.39; 2.30] | 0.42 [0.03; 5.36] | Somatrogon 0.66 mg/kg/wk | . |
| YPEG-rhGH 0.14 mg/kg/wk | 1.32 [0.83; 2.09] | 0.58 [0.05; 6.65] | 1.39 [0.51; 3.77] | YPEG-rhGH 0.14 mg/kg/wk |

S6.13 League table for URTI

|  | Daily rhGH | Lonapegsomatropin 0.24 mg/kg/wk | Somapacitan 0.04 mg/kg/wk | Somapacitan 0.08 mg/kg/wk | Somapacitan 0.16 mg/kg/wk | YPEG-rhGH 0.14 mg/kg/wk |
| --- | --- | --- | --- | --- | --- | --- |
| Daily rhGH | Daily rhGH | 1.11 [0.74; 1.65] | 2.27 [0.21; 24.93] | 1.10 [0.18; 6.87] | 1.73 [0.47; 6.38] | 0.90 [0.72; 1.13] |
| Lonapegsomatropin 0.24 mg/kg/wk | 1.11 [0.74; 1.65] | Lonapegsomatropin 0.24 mg/kg/wk | . | . | . | . |
| Somapacitan 0.04 mg/kg/wk | 2.27 [0.21; 24.93] | 2.06 [0.18; 23.31] | Somapacitan 0.04 mg/kg/wk | . | . | . |
| Somapacitan 0.08 mg/kg/wk | 1.10 [0.18; 6.87] | 0.99 [0.15; 6.49] | 0.48 [0.02; 9.87] | Somapacitan 0.08 mg/kg/wk | . | . |
| Somapacitan 0.16 mg/kg/wk | 1.73 [0.47; 6.38] | 1.57 [0.40; 6.13] | 0.76 [0.05; 11.65] | 1.58 [0.17; 14.95] | Somapacitan 0.16 mg/kg/wk | . |
| YPEG-rhGH 0.14 mg/kg/wk | 0.90 [0.72; 1.13] | 0.82 [0.52; 1.29] | 0.40 [0.04; 4.41] | 0.82 [0.13; 5.21] | 0.52 [0.14; 1.96] | YPEG-rhGH 0.14 mg/kg/wk |

S6.14 league table for FPG

|  | Daily rhGH | Lonapegsomatropin 0.24 mg/kg/wk | PEG-rhGH 0.2 mg/kg/wk | TransCon GH 0.14 mg/kg/wk | TransCon GH 0.21 mg/kg/wk | TransCon GH 0.30 mg/kg/wk |
| --- | --- | --- | --- | --- | --- | --- |
| Daily rhGH | Daily rhGH | 3.10 [ 0.24; 5.96] | -0.06 [ -0.20; 0.08] | -7.00 [-14.92; 0.92] | -8.30 [-17.00; 0.40] | -10.40 [-30.78; 9.98] |
| Lonapegsomatropin 0.24 mg/kg/wk | 3.10 [ 0.24; 5.96] | Lonapegsomatropin 0.24 mg/kg/wk | . | . | . | . |
| PEG-rhGH 0.2 mg/kg/wk | -0.06 [ -0.20; 0.08] | -3.16 [ -6.02; -0.30] | PEG-rhGH 0.2 mg/kg/wk | . | . | . |
| TransCon GH 0.14 mg/kg/wk | -7.00 [-14.92; 0.92] | -10.10 [-18.52; -1.68] | -6.94 [-14.86; 0.98] | TransCon GH 0.14 mg/kg/wk | . | . |
| TransCon GH 0.21 mg/kg/wk | -8.30 [-17.00; 0.40] | -11.40 [-20.56; -2.24] | -8.24 [-16.94; 0.46] | -1.30 [-13.07; 10.47] | TransCon GH 0.21 mg/kg/wk | . |
| TransCon GH 0.30 mg/kg/wk | -10.40 [-30.78; 9.98] | -13.50 [-34.08; 7.08] | -10.34 [-30.72; 10.04] | -3.40 [-25.27; 18.47] | -2.10 [-24.26; 20.06] | TransCon GH 0.30 mg/kg/wk |

**Supplement 7: Treatment ranking for each outcome**

S7.1 Treatment ranking for Height Velocity

| Medication | P-score |
| --- | --- |
| Somapacitan 0.04mg/kg/wk | 0.8829 |
| Somatrogon 0.25/0.48/0.66 mg/kg/wk | 0.8682 |
| TransCon GH 0.30 mg/kg/wk | 0.8083 |
| Somapacitan 0.04/0.16 mg/kg/wk | 0.7416 |
| TransCon GH 0.21 mg/kg/wk | 0.6749 |
| PEG-rhGH 0.2 mg/kg/wk | 0.5405 |
| Somapacitan 0.08/0.16 mg/kg/wk | 0.5317 |
| TransCon GH 0.14 mg/kg/wk | 0.5280 |
| Somapacitan 0.08 mg/kg/wk | 0.5170 |
| YPEG-rhGH 0.14 mg/kg/wk | 0.4850 |
| Daily rhGH | 0.4777 |
| PEG-rhGH 0.12 mg/kg/wk | 0.4228 |
| Somapacitan 0.16 mg/kg/wk | 0.4014 |
| Somatrogon 0.66 mg/kg/wk | 0.3985 |
| YPEG-rhGH 0.12 mg/kg/wk | 0.2972 |
| Lonapegsomatropin 0.24 mg/kg/wk | 0.2829 |
| PEG-rhGH 0.1 mg/kg/wk | 0.1154 |
| YPEG-rhGH 0.1 mg/kg/wk | 0.0260 |

S7.2 Treatment ranking for Height SDS

| Medication | P-score |
| --- | --- |
| TransCon GH 0.30 mg/kg/wk | 0.8781 |
| TransCon GH 0.21 mg/kg/wk | 0.6916 |
| Somapacitan 0.08/0.16 mg/kg/wk | 0.6476 |
| Daily rhGH | 0.6074 |
| Somatrogon 0.66 mg/kg/wk | 0.5721 |
| PEG-rhGH 0.2 mg/kg/wk | 0.5481 |
| Somapacitan 0.16 mg/kg/wk | 0.5454 |
| Lonapegsomatropin 0.24 mg/kg/wk | 0.5213 |
| TransCon GH 0.14 mg/kg/wk | 0.5135 |
| YPEG-rhGH 0.14 mg/kg/wk | 0.4633 |
| YPEG-rhGH 0.12 mg/kg/wk | 0.4182 |
| Somapacitan 0.04/0.16 mg/kg/wk | 0.3022 |
| YPEG-rhGH 0.1 mg/kg/wk | 0.1795 |
| PEG-rhGH 0.1 mg/kg/wk | 0.1117 |

S7.3 Treatment ranking for Treatment discontinuation

| Medication | P-score |
| --- | --- |
| YPEG-rhGH 0.1 mg/kg/wk | 0.7341 |
| YPEG-rhGH 0.12 mg/kg/wk | 0.7341 |
| PEG-rhGH 0.2 mg/kg/wk | 0.5283 |
| PEG-rhGH 0.1 mg/kg/wk | 0.5283 |
| YPEG-rhGH 0.14 mg/kg/wk | 0.5268 |
| Somapacitan 0.16 mg/kg/wk | 0.5239 |
| Lonapegsomatropin 0.24 mg/kg/wk | 0.5167 |
| TransCon GH 0.14 mg/kg/wk | 0.3992 |
| TransCon GH 0.21 mg/kg/wk | 0.3992 |
| TransCon GH 0.30 mg/kg/wk | 0.3992 |
| Somatrogon 0.66 mg/kg/wk | 0.3746 |
| Daily rhGH | 0.3354 |

S7.4 Treatment ranking for Injection site erythema

| Medication | P-score |
| --- | --- |
| Somapacitan 0.16 mg/kg/wk | 0.8676 |
| PEG-rhGH 0.1 mg/kg/wk | 0.6861 |
| PEG-rhGH 0.2 mg/kg/wk | 0.6861 |
| TransCon GH 0.21 mg/kg/wk | 0.4880 |
| TransCon GH 0.30 mg/kg/wk | 0.4880 |

S7.5 Treatment ranking for Influenza

| Medication | P-score |
| --- | --- |
| Somapacitan 0.16 mg/kg/wk | 0.7124 |
| Lonapegsomatropin 0.24 mg/kg/wk | 0.5206 |
| Somapacitan 0.08 mg/kg/wk | 0.5098 |
| Daily rhGH | 0.4902 |
| Somatrogon 0.25 mg/kg/wk | 0.4224 |
| Somatrogon 0.48 mg/kg/wk | 0.4224 |
| Somatrogon 0.66 mg/kg/wk | 0.4224 |

S7.6 Treatment ranking for Headache

| Medication | P-score |
| --- | --- |
| Somatrogon 0.66 mg/kg/wk | 0.7510 |
| TransCon GH 0.30 mg/kg/wk | 0.5841 |
| Lonapegsomatropin 0.24 mg/kg/wk | 0.5798 |
| Daily rhGH | 0.5583 |
| PEG-rhGH 0.2 mg/kg/wk | 0.3479 |
| PEG-rhGH 0.1 mg/kg/wk | 0.3479 |
| TransCon GH 0.14 mg/kg/wk | 0.3310 |

S7.7 Treatment ranking for Fever

| Medication | P-score |
| --- | --- |
| TransCon GH 0.30 mg/kg/wk | 0.8629 |
| YPEG-rhGH 0.14 mg/kg/wk | 0.7645 |
| Daily rhGH | 0.4846 |
| Somatrogon 0.66 mg/kg/wk | 0.3600 |
| Somatrogon 0.25/0.48/0.66 mg/kg/wk | 0.3452 |
| Lonapegsomatropin 0.24 mg/kg/wk | 0.1827 |

S7.8 Treatment ranking for hypothyroidism

| Medication | P-score |
| --- | --- |
| YPEG-rhGH 0.12 mg/kg/wk | 0.8136 |
| YPEG-rhGH 0.14 mg/kg/wk | 0.8136 |
| PEG-rhGH 0.1 mg/kg/wk | 0.6502 |
| PEG-rhGH 0.2 mg/kg/wk | 0.6502 |
| Daily rhGH | 0.4670 |
| Somatrogon 0.25 mg/kg/wk | 0.2057 |
| Somatrogon 0.48 mg/kg/wk | 0.2057 |
| Somatrogon 0.66 mg/kg/wk | 0.1940 |

S7.9 Treatment ranking for Injection site pain

| Medication | P-score |
| --- | --- |
| TransCon GH 0.21 mg/kg/wk | 0.7228 |
| TransCon GH 0.14 mg/kg/wk | 0.6628 |
| Somapacitan 0.16 mg/kg/wk | 0.6614 |
| TransCon GH 0.30 mg/kg/wk | 0.6523 |
| Daily rhGH | 0.6256 |
| YPEG-rhGH 0.14 mg/kg/wk | 0.5542 |
| YPEG-rhGH 0.12 mg/kg/wk | 0.5542 |
| YPEG-rhGH 0.1 mg/kg/wk | 0.5542 |
| Somatrogon 0.66 mg/kg/wk | 0.3104 |
| Somatrogon 0.48 mg/kg/wk | 0.1010 |
| Somatrogon 0.25 mg/kg/wk | 0.1010 |

S7.10 Treatment ranking for IGF-1

| Medication | P-score |
| --- | --- |
| Somatrogon 0.25/0.48/0.66 mg/kg/wk | 0.9822 |
| Somapacitan 0.04mg/kg/wk | 0.9162 |
| PEG-rhGH 0.1 mg/kg/wk | 0.7223 |
| Somapacitan 0.16/0.16 mg/kg/wk | 0.6693 |
| Daily rhGH | 0.5316 |
| Somapacitan 0.08 mg/kg/wk | 0.4433 |
| PEG-rhGH 0.2 mg/kg/wk | 0.4315 |
| Somapacitan 0.08/0.16 mg/kg/wk | 0.3410 |
| Somapacitan 0.04/0.16 mg/kg/wk | 0.3052 |
| Somapacitan 0.16 mg/kg/wk | 0.3005 |
| YPEG-rhGH 0.14 mg/kg/wk | 0.2979 |
| Lonapegsomatropin 0.24 mg/kg/wk | 0.0589 |

S7.11 Treatment ranking for HbA1C%

| Medication | P-score |
| --- | --- |
| TransCon GH 0.30 mg/kg/wk | 0.8194 |
| Daily rhGH | 0.5830 |
| TransCon GH 0.21 mg/kg/wk | 0.5272 |
| TransCon GH 0.14 mg/kg/wk | 0.5242 |
| PEG-rhGH 0.2 mg/kg/wk | 0.3598 |
| Lonapegsomatropin 0.24 mg/kg/wk | 0.1864 |

S7.12 Treatment ranking for cough

| Medication | P-score |
| --- | --- |
| Somapacitan 0.04mg/kg/wk | 0.7224 |
| YPEG-rhGH 0.14 mg/kg/wk | 0.6513 |
| Somatrogon 0.66 mg/kg/wk | 0.3207 |
| Daily rhGH | 0.3056 |

S7.13 Treatment ranking for URTI

| Medication | P-score |
| --- | --- |
| Somapacitan 0.04 mg/kg/wk | 0.7004 |
| Somapacitan 0.16 mg/kg/wk | 0.6887 |
| Lonapegsomatropin 0.24 mg/kg/wk | 0.5075 |
| Somapacitan 0.08 mg/kg/wk | 0.4569 |
| Daily rhGH | 0.4074 |
| YPEG-rhGH 0.14 mg/kg/wk | 0.2392 |

S7.14 Treatment ranking for FPG

| Medication | P-score |
| --- | --- |
| Lonapegsomatropin 0.24 mg/kg/wk | 0.9704 |
| Daily rhGH | 0.7180 |
| PEG-rhGH 0.2 mg/kg/wk | 0.5952 |
| TransCon GH 0.14 mg/kg/wk | 0.2599 |
| TransCon GH 0.30 mg/kg/wk | 0.2449 |
| TransCon GH 0.21 mg/kg/wk | 0.2116 |

**Supplement 8: Certainty of evidence for each outcome**

S8.1 Certainty of evidence for Height Velocity

| Comparison | Within_Study_Bias | Reporting_Bias | Indirectness | Imprecision | Heterogeneity | Incoherence | Overall_Confidence | Reason_for_downgrade |
| --- | --- | --- | --- | --- | --- | --- | --- | --- |
| Daily rhGH:PEG-rhGH 0.1 mg/kg/wk | No concerns | Some concerns | No concerns | Major concerns | Major concerns | No concerns | Very low | Reporting bias, Imprecision, Heterogeneity |
| Daily rhGH:PEG-rhGH 0.2 mg/kg/wk | No concerns | Some concerns | No concerns | Major concerns | Major concerns | No concerns | Very low | Reporting bias, Imprecision, Heterogeneity |
| Daily rhGH:TransCon GH 0.14 mg/kg/wk | No concerns | Some concerns | Some concerns | Major concerns | Major concerns | No concerns | Very low | Reporting bias, Indirectness, Imprecision, Heterogeneity |
| Daily rhGH:TransCon GH 0.21 mg/kg/wk | No concerns | Some concerns | Some concerns | Major concerns | Major concerns | No concerns | Very low | Reporting bias, Indirectness, Imprecision, Heterogeneity |
| Daily rhGH:TransCon GH 0.30 mg/kg/wk | No concerns | Some concerns | Some concerns | Major concerns | Major concerns | No concerns | Very low | Reporting bias, Indirectness, Imprecision, Heterogeneity |
| Daily rhGH:Somatrogon 0.66 mg/kg/wk | No concerns | Some concerns | No concerns | Major concerns | Major concerns | No concerns | Very low | Reporting bias, Imprecision, Heterogeneity |
| Daily rhGH:PEG-rhGH 0.12 mg/kg/wk | Some concerns | Some concerns | No concerns | Major concerns | Major concerns | No concerns | Very low | Within-study bias, Reporting bias, Imprecision, Heterogeneity |
| Daily rhGH:Somapacitan 0.16 mg/kg/wk | Some concerns | Some concerns | No concerns | Major concerns | Major concerns | No concerns | Very low | Within-study bias, Reporting bias, Imprecision, Heterogeneity |
| Daily rhGH:Somatrogon 0.25/0.48/0.66 mg/kg/wk | No concerns | Some concerns | No concerns | Some concerns | Major concerns | No concerns | Low | Reporting bias, Imprecision, Heterogeneity |
| Daily rhGH:YPEG-rhGH 0.1 mg/kg/wk | No concerns | Some concerns | Some concerns | Major concerns | Major concerns | No concerns | Very low | Reporting bias, Indirectness, Imprecision, Heterogeneity |
| Daily rhGH:YPEG-rhGH 0.12 mg/kg/wk | No concerns | Some concerns | Some concerns | Major concerns | Major concerns | No concerns | Very low | Reporting bias, Indirectness, Imprecision, Heterogeneity |
| Daily rhGH:YPEG-rhGH 0.14 mg/kg/wk | No concerns | Some concerns | Some concerns | Major concerns | Major concerns | No concerns | Very low | Reporting bias, Indirectness, Imprecision, Heterogeneity |
| Daily rhGH:Somapacitan 0.08 mg/kg/wk | Major concerns | Some concerns | Some concerns | Major concerns | Major concerns | No concerns | Very low | Within-study bias, Reporting bias, Indirectness, Imprecision, Heterogeneity |
| Daily rhGH:Somapacitan 0.04mg/kg/wk | Major concerns | Some concerns | Some concerns | Some concerns | Major concerns | No concerns | Very low | Within-study bias, Reporting bias, Indirectness, Imprecision, Heterogeneity |
| Daily rhGH:Somapacitan 0.04/0.16 mg/kg/wk | Major concerns | Some concerns | No concerns | Some concerns | Major concerns | No concerns | Very low | Within-study bias, Reporting bias, Imprecision, Heterogeneity |
| Daily rhGH:Somapacitan 0.08/0.16 mg/kg/wk | Major concerns | Some concerns | No concerns | Major concerns | Major concerns | No concerns | Very low | Within-study bias, Reporting bias, Imprecision, Heterogeneity |
| Daily rhGH:Lonapegsomatropin 0.24 mg/kg/wk | No concerns | Some concerns | No concerns | Major concerns | Major concerns | No concerns | Very low | Reporting bias, Imprecision, Heterogeneity |
| PEG-rhGH 0.1 mg/kg/wk:PEG-rhGH 0.2 mg/kg/wk | No concerns | Some concerns | No concerns | Some concerns | Major concerns | No concerns | Low | Reporting bias, Imprecision, Heterogeneity |
| PEG-rhGH 0.1 mg/kg/wk:TransCon GH 0.14 mg/kg/wk | No concerns | Some concerns | No concerns | Major concerns | Major concerns | No concerns | Very low | Reporting bias, Imprecision, Heterogeneity |
| PEG-rhGH 0.1 mg/kg/wk:TransCon GH 0.21 mg/kg/wk | No concerns | Some concerns | No concerns | Some concerns | Major concerns | No concerns | Low | Reporting bias, Imprecision, Heterogeneity |
| PEG-rhGH 0.1 mg/kg/wk:TransCon GH 0.30 mg/kg/wk | No concerns | Some concerns | No concerns | Some concerns | Major concerns | No concerns | Low | Reporting bias, Imprecision, Heterogeneity |
| PEG-rhGH 0.1 mg/kg/wk:Somatrogon 0.66 mg/kg/wk | No concerns | Some concerns | No concerns | Some concerns | Major concerns | No concerns | Low | Reporting bias, Imprecision, Heterogeneity |
| PEG-rhGH 0.1 mg/kg/wk:PEG-rhGH 0.12 mg/kg/wk | No concerns | Some concerns | No concerns | Some concerns | Major concerns | No concerns | Low | Reporting bias, Imprecision, Heterogeneity |
| PEG-rhGH 0.1 mg/kg/wk:Somapacitan 0.16 mg/kg/wk | Some concerns | Some concerns | No concerns | Some concerns | Major concerns | No concerns | Low | Within-study bias, Reporting bias, Imprecision, Heterogeneity |
| PEG-rhGH 0.1 mg/kg/wk:Somatrogon 0.25/0.48/0.66 mg/kg/wk | No concerns | Some concerns | No concerns | Some concerns | Major concerns | No concerns | Low | Reporting bias, Imprecision, Heterogeneity |
| PEG-rhGH 0.1 mg/kg/wk:YPEG-rhGH 0.1 mg/kg/wk | No concerns | Some concerns | No concerns | Major concerns | Major concerns | No concerns | Very low | Reporting bias, Imprecision, Heterogeneity |
| PEG-rhGH 0.1 mg/kg/wk:YPEG-rhGH 0.12 mg/kg/wk | No concerns | Some concerns | No concerns | Major concerns | Major concerns | No concerns | Very low | Reporting bias, Imprecision, Heterogeneity |
| PEG-rhGH 0.1 mg/kg/wk:YPEG-rhGH 0.14 mg/kg/wk | No concerns | Some concerns | No concerns | Major concerns | Major concerns | No concerns | Very low | Reporting bias, Imprecision, Heterogeneity |
| PEG-rhGH 0.1 mg/kg/wk:Somapacitan 0.08 mg/kg/wk | Some concerns | Some concerns | No concerns | Some concerns | Major concerns | No concerns | Low | Within-study bias, Reporting bias, Imprecision, Heterogeneity |
| PEG-rhGH 0.1 mg/kg/wk:Somapacitan 0.04mg/kg/wk | Some concerns | Some concerns | No concerns | Some concerns | Major concerns | No concerns | Low | Within-study bias, Reporting bias, Imprecision, Heterogeneity |
| PEG-rhGH 0.1 mg/kg/wk:Somapacitan 0.04/0.16 mg/kg/wk | Some concerns | Some concerns | No concerns | Some concerns | Major concerns | No concerns | Low | Within-study bias, Reporting bias, Imprecision, Heterogeneity |
| PEG-rhGH 0.1 mg/kg/wk:Somapacitan 0.08/0.16 mg/kg/wk | Some concerns | Some concerns | No concerns | Some concerns | Major concerns | No concerns | Low | Within-study bias, Reporting bias, Imprecision, Heterogeneity |
| Lonapegsomatropin 0.24 mg/kg/wk:PEG-rhGH 0.1 mg/kg/wk | No concerns | Some concerns | No concerns | Major concerns | Major concerns | No concerns | Very low | Reporting bias, Imprecision, Heterogeneity |
| PEG-rhGH 0.2 mg/kg/wk:TransCon GH 0.14 mg/kg/wk | No concerns | Some concerns | No concerns | Major concerns | Major concerns | No concerns | Very low | Reporting bias, Imprecision, Heterogeneity |
| PEG-rhGH 0.2 mg/kg/wk:TransCon GH 0.21 mg/kg/wk | No concerns | Some concerns | No concerns | Major concerns | Major concerns | No concerns | Very low | Reporting bias, Imprecision, Heterogeneity |
| PEG-rhGH 0.2 mg/kg/wk:TransCon GH 0.30 mg/kg/wk | No concerns | Some concerns | No concerns | Major concerns | Major concerns | No concerns | Very low | Reporting bias, Imprecision, Heterogeneity |
| PEG-rhGH 0.2 mg/kg/wk:Somatrogon 0.66 mg/kg/wk | No concerns | Some concerns | No concerns | Major concerns | Major concerns | No concerns | Very low | Reporting bias, Imprecision, Heterogeneity |
| PEG-rhGH 0.12 mg/kg/wk:PEG-rhGH 0.2 mg/kg/wk | Some concerns | Some concerns | No concerns | Major concerns | Major concerns | No concerns | Very low | Within-study bias, Reporting bias, Imprecision, Heterogeneity |
| PEG-rhGH 0.2 mg/kg/wk:Somapacitan 0.16 mg/kg/wk | Some concerns | Some concerns | No concerns | Major concerns | Major concerns | No concerns | Very low | Within-study bias, Reporting bias, Imprecision, Heterogeneity |
| PEG-rhGH 0.2 mg/kg/wk:Somatrogon 0.25/0.48/0.66 mg/kg/wk | No concerns | Some concerns | No concerns | Some concerns | Major concerns | No concerns | Low | Reporting bias, Imprecision, Heterogeneity |
| PEG-rhGH 0.2 mg/kg/wk:YPEG-rhGH 0.1 mg/kg/wk | No concerns | Some concerns | No concerns | Major concerns | Major concerns | No concerns | Very low | Reporting bias, Imprecision, Heterogeneity |
| PEG-rhGH 0.2 mg/kg/wk:YPEG-rhGH 0.12 mg/kg/wk | No concerns | Some concerns | No concerns | Major concerns | Major concerns | No concerns | Very low | Reporting bias, Imprecision, Heterogeneity |
| PEG-rhGH 0.2 mg/kg/wk:YPEG-rhGH 0.14 mg/kg/wk | No concerns | Some concerns | No concerns | Major concerns | Major concerns | No concerns | Very low | Reporting bias, Imprecision, Heterogeneity |
| PEG-rhGH 0.2 mg/kg/wk:Somapacitan 0.08 mg/kg/wk | Some concerns | Some concerns | No concerns | Major concerns | Major concerns | No concerns | Very low | Within-study bias, Reporting bias, Imprecision, Heterogeneity |
| PEG-rhGH 0.2 mg/kg/wk:Somapacitan 0.04mg/kg/wk | Some concerns | Some concerns | No concerns | Some concerns | Major concerns | No concerns | Low | Within-study bias, Reporting bias, Imprecision, Heterogeneity |
| PEG-rhGH 0.2 mg/kg/wk:Somapacitan 0.04/0.16 mg/kg/wk | Some concerns | Some concerns | No concerns | Major concerns | Major concerns | No concerns | Very low | Within-study bias, Reporting bias, Imprecision, Heterogeneity |
| PEG-rhGH 0.2 mg/kg/wk:Somapacitan 0.08/0.16 mg/kg/wk | Some concerns | Some concerns | No concerns | Major concerns | Major concerns | No concerns | Very low | Within-study bias, Reporting bias, Imprecision, Heterogeneity |
| Lonapegsomatropin 0.24 mg/kg/wk:PEG-rhGH 0.2 mg/kg/wk | No concerns | Some concerns | No concerns | Major concerns | Major concerns | No concerns | Very low | Reporting bias, Imprecision, Heterogeneity |
| TransCon GH 0.14 mg/kg/wk:TransCon GH 0.21 mg/kg/wk | No concerns | Some concerns | Some concerns | Major concerns | Major concerns | No concerns | Very low | Reporting bias, Indirectness, Imprecision, Heterogeneity |
| TransCon GH 0.14 mg/kg/wk:TransCon GH 0.30 mg/kg/wk | No concerns | Some concerns | Some concerns | Major concerns | Major concerns | No concerns | Very low | Reporting bias, Indirectness, Imprecision, Heterogeneity |
| Somatrogon 0.66 mg/kg/wk:TransCon GH 0.14 mg/kg/wk | No concerns | Some concerns | No concerns | Major concerns | Major concerns | No concerns | Very low | Reporting bias, Imprecision, Heterogeneity |
| PEG-rhGH 0.12 mg/kg/wk:TransCon GH 0.14 mg/kg/wk | No concerns | Some concerns | No concerns | Major concerns | Major concerns | No concerns | Very low | Reporting bias, Imprecision, Heterogeneity |
| Somapacitan 0.16 mg/kg/wk:TransCon GH 0.14 mg/kg/wk | Some concerns | Some concerns | Some concerns | Major concerns | Major concerns | No concerns | Very low | Within-study bias, Reporting bias, Indirectness, Imprecision, Heterogeneity |
| Somatrogon 0.25/0.48/0.66 mg/kg/wk:TransCon GH 0.14 mg/kg/wk | No concerns | Some concerns | No concerns | Major concerns | Major concerns | No concerns | Very low | Reporting bias, Imprecision, Heterogeneity |
| TransCon GH 0.14 mg/kg/wk:YPEG-rhGH 0.1 mg/kg/wk | No concerns | Some concerns | Some concerns | Major concerns | Major concerns | No concerns | Very low | Reporting bias, Indirectness, Imprecision, Heterogeneity |
| TransCon GH 0.14 mg/kg/wk:YPEG-rhGH 0.12 mg/kg/wk | No concerns | Some concerns | Some concerns | Major concerns | Major concerns | No concerns | Very low | Reporting bias, Indirectness, Imprecision, Heterogeneity |
| TransCon GH 0.14 mg/kg/wk:YPEG-rhGH 0.14 mg/kg/wk | No concerns | Some concerns | Some concerns | Major concerns | Major concerns | No concerns | Very low | Reporting bias, Indirectness, Imprecision, Heterogeneity |
| Somapacitan 0.08 mg/kg/wk:TransCon GH 0.14 mg/kg/wk | Some concerns | Some concerns | Some concerns | Major concerns | Major concerns | No concerns | Very low | Within-study bias, Reporting bias, Indirectness, Imprecision, Heterogeneity |
| Somapacitan 0.04mg/kg/wk:TransCon GH 0.14 mg/kg/wk | Some concerns | Some concerns | Some concerns | Major concerns | Major concerns | No concerns | Very low | Within-study bias, Reporting bias, Indirectness, Imprecision, Heterogeneity |
| Somapacitan 0.04/0.16 mg/kg/wk:TransCon GH 0.14 mg/kg/wk | Some concerns | Some concerns | No concerns | Major concerns | Major concerns | No concerns | Very low | Within-study bias, Reporting bias, Imprecision, Heterogeneity |
| Somapacitan 0.08/0.16 mg/kg/wk:TransCon GH 0.14 mg/kg/wk | Some concerns | Some concerns | No concerns | Major concerns | Major concerns | No concerns | Very low | Within-study bias, Reporting bias, Imprecision, Heterogeneity |
| Lonapegsomatropin 0.24 mg/kg/wk:TransCon GH 0.14 mg/kg/wk | No concerns | Some concerns | No concerns | Major concerns | Major concerns | No concerns | Very low | Reporting bias, Imprecision, Heterogeneity |
| TransCon GH 0.21 mg/kg/wk:TransCon GH 0.30 mg/kg/wk | No concerns | Some concerns | Some concerns | Major concerns | Major concerns | No concerns | Very low | Reporting bias, Indirectness, Imprecision, Heterogeneity |
| Somatrogon 0.66 mg/kg/wk:TransCon GH 0.21 mg/kg/wk | No concerns | Some concerns | No concerns | Major concerns | Major concerns | No concerns | Very low | Reporting bias, Imprecision, Heterogeneity |
| PEG-rhGH 0.12 mg/kg/wk:TransCon GH 0.21 mg/kg/wk | No concerns | Some concerns | No concerns | Major concerns | Major concerns | No concerns | Very low | Reporting bias, Imprecision, Heterogeneity |
| Somapacitan 0.16 mg/kg/wk:TransCon GH 0.21 mg/kg/wk | Some concerns | Some concerns | Some concerns | Major concerns | Major concerns | No concerns | Very low | Within-study bias, Reporting bias, Indirectness, Imprecision, Heterogeneity |
| Somatrogon 0.25/0.48/0.66 mg/kg/wk:TransCon GH 0.21 mg/kg/wk | No concerns | Some concerns | No concerns | Major concerns | Major concerns | No concerns | Very low | Reporting bias, Imprecision, Heterogeneity |
| TransCon GH 0.21 mg/kg/wk:YPEG-rhGH 0.1 mg/kg/wk | No concerns | Some concerns | Some concerns | Some concerns | Major concerns | No concerns | Low | Reporting bias, Indirectness, Imprecision, Heterogeneity |
| TransCon GH 0.21 mg/kg/wk:YPEG-rhGH 0.12 mg/kg/wk | No concerns | Some concerns | Some concerns | Major concerns | Major concerns | No concerns | Very low | Reporting bias, Indirectness, Imprecision, Heterogeneity |
| TransCon GH 0.21 mg/kg/wk:YPEG-rhGH 0.14 mg/kg/wk | No concerns | Some concerns | Some concerns | Major concerns | Major concerns | No concerns | Very low | Reporting bias, Indirectness, Imprecision, Heterogeneity |
| Somapacitan 0.08 mg/kg/wk:TransCon GH 0.21 mg/kg/wk | Some concerns | Some concerns | Some concerns | Major concerns | Major concerns | No concerns | Very low | Within-study bias, Reporting bias, Indirectness, Imprecision, Heterogeneity |
| Somapacitan 0.04mg/kg/wk:TransCon GH 0.21 mg/kg/wk | Some concerns | Some concerns | Some concerns | Major concerns | Major concerns | No concerns | Very low | Within-study bias, Reporting bias, Indirectness, Imprecision, Heterogeneity |
| Somapacitan 0.04/0.16 mg/kg/wk:TransCon GH 0.21 mg/kg/wk | Some concerns | Some concerns | No concerns | Major concerns | Major concerns | No concerns | Very low | Within-study bias, Reporting bias, Imprecision, Heterogeneity |
| Somapacitan 0.08/0.16 mg/kg/wk:TransCon GH 0.21 mg/kg/wk | Some concerns | Some concerns | No concerns | Major concerns | Major concerns | No concerns | Very low | Within-study bias, Reporting bias, Imprecision, Heterogeneity |
| Lonapegsomatropin 0.24 mg/kg/wk:TransCon GH 0.21 mg/kg/wk | No concerns | Some concerns | No concerns | Major concerns | Major concerns | No concerns | Very low | Reporting bias, Imprecision, Heterogeneity |
| Somatrogon 0.66 mg/kg/wk:TransCon GH 0.30 mg/kg/wk | No concerns | Some concerns | No concerns | Major concerns | Major concerns | No concerns | Very low | Reporting bias, Imprecision, Heterogeneity |
| PEG-rhGH 0.12 mg/kg/wk:TransCon GH 0.30 mg/kg/wk | No concerns | Some concerns | No concerns | Major concerns | Major concerns | No concerns | Very low | Reporting bias, Imprecision, Heterogeneity |
| Somapacitan 0.16 mg/kg/wk:TransCon GH 0.30 mg/kg/wk | Some concerns | Some concerns | Some concerns | Some concerns | Major concerns | No concerns | Low | Within-study bias, Reporting bias, Indirectness, Imprecision, Heterogeneity |
| Somatrogon 0.25/0.48/0.66 mg/kg/wk:TransCon GH 0.30 mg/kg/wk | No concerns | Some concerns | No concerns | Major concerns | Major concerns | No concerns | Very low | Reporting bias, Imprecision, Heterogeneity |
| TransCon GH 0.30 mg/kg/wk:YPEG-rhGH 0.1 mg/kg/wk | No concerns | Some concerns | Some concerns | Some concerns | Major concerns | No concerns | Low | Reporting bias, Indirectness, Imprecision, Heterogeneity |
| TransCon GH 0.30 mg/kg/wk:YPEG-rhGH 0.12 mg/kg/wk | No concerns | Some concerns | Some concerns | Major concerns | Major concerns | No concerns | Very low | Reporting bias, Indirectness, Imprecision, Heterogeneity |
| TransCon GH 0.30 mg/kg/wk:YPEG-rhGH 0.14 mg/kg/wk | No concerns | Some concerns | Some concerns | Major concerns | Major concerns | No concerns | Very low | Reporting bias, Indirectness, Imprecision, Heterogeneity |
| Somapacitan 0.08 mg/kg/wk:TransCon GH 0.30 mg/kg/wk | Some concerns | Some concerns | Some concerns | Major concerns | Major concerns | No concerns | Very low | Within-study bias, Reporting bias, Indirectness, Imprecision, Heterogeneity |
| Somapacitan 0.04mg/kg/wk:TransCon GH 0.30 mg/kg/wk | Some concerns | Some concerns | Some concerns | Major concerns | Major concerns | No concerns | Very low | Within-study bias, Reporting bias, Indirectness, Imprecision, Heterogeneity |
| Somapacitan 0.04/0.16 mg/kg/wk:TransCon GH 0.30 mg/kg/wk | Some concerns | Some concerns | No concerns | Major concerns | Major concerns | No concerns | Very low | Within-study bias, Reporting bias, Imprecision, Heterogeneity |
| Somapacitan 0.08/0.16 mg/kg/wk:TransCon GH 0.30 mg/kg/wk | Some concerns | Some concerns | No concerns | Major concerns | Major concerns | No concerns | Very low | Within-study bias, Reporting bias, Imprecision, Heterogeneity |
| Lonapegsomatropin 0.24 mg/kg/wk:TransCon GH 0.30 mg/kg/wk | No concerns | Some concerns | No concerns | Some concerns | Major concerns | No concerns | Low | Reporting bias, Imprecision, Heterogeneity |
| PEG-rhGH 0.12 mg/kg/wk:Somatrogon 0.66 mg/kg/wk | No concerns | Some concerns | No concerns | Major concerns | Major concerns | No concerns | Very low | Reporting bias, Imprecision, Heterogeneity |
| Somapacitan 0.16 mg/kg/wk:Somatrogon 0.66 mg/kg/wk | Some concerns | Some concerns | No concerns | Major concerns | Major concerns | No concerns | Very low | Within-study bias, Reporting bias, Imprecision, Heterogeneity |
| Somatrogon 0.25/0.48/0.66 mg/kg/wk:Somatrogon 0.66 mg/kg/wk | No concerns | Some concerns | No concerns | Major concerns | Major concerns | No concerns | Very low | Reporting bias, Imprecision, Heterogeneity |
| Somatrogon 0.66 mg/kg/wk:YPEG-rhGH 0.1 mg/kg/wk | No concerns | Some concerns | No concerns | Major concerns | Major concerns | No concerns | Very low | Reporting bias, Imprecision, Heterogeneity |
| Somatrogon 0.66 mg/kg/wk:YPEG-rhGH 0.12 mg/kg/wk | No concerns | Some concerns | No concerns | Major concerns | Major concerns | No concerns | Very low | Reporting bias, Imprecision, Heterogeneity |
| Somatrogon 0.66 mg/kg/wk:YPEG-rhGH 0.14 mg/kg/wk | No concerns | Some concerns | No concerns | Major concerns | Major concerns | No concerns | Very low | Reporting bias, Imprecision, Heterogeneity |
| Somapacitan 0.08 mg/kg/wk:Somatrogon 0.66 mg/kg/wk | Some concerns | Some concerns | No concerns | Major concerns | Major concerns | No concerns | Very low | Within-study bias, Reporting bias, Imprecision, Heterogeneity |
| Somapacitan 0.04mg/kg/wk:Somatrogon 0.66 mg/kg/wk | Some concerns | Some concerns | No concerns | Major concerns | Major concerns | No concerns | Very low | Within-study bias, Reporting bias, Imprecision, Heterogeneity |
| Somapacitan 0.04/0.16 mg/kg/wk:Somatrogon 0.66 mg/kg/wk | Some concerns | Some concerns | No concerns | Major concerns | Major concerns | No concerns | Very low | Within-study bias, Reporting bias, Imprecision, Heterogeneity |
| Somapacitan 0.08/0.16 mg/kg/wk:Somatrogon 0.66 mg/kg/wk | Some concerns | Some concerns | No concerns | Major concerns | Major concerns | No concerns | Very low | Within-study bias, Reporting bias, Imprecision, Heterogeneity |
| Lonapegsomatropin 0.24 mg/kg/wk:Somatrogon 0.66 mg/kg/wk | No concerns | Some concerns | No concerns | Major concerns | Major concerns | No concerns | Very low | Reporting bias, Imprecision, Heterogeneity |
| PEG-rhGH 0.12 mg/kg/wk:Somapacitan 0.16 mg/kg/wk | Some concerns | Some concerns | No concerns | Major concerns | Major concerns | No concerns | Very low | Within-study bias, Reporting bias, Imprecision, Heterogeneity |
| PEG-rhGH 0.12 mg/kg/wk:Somatrogon 0.25/0.48/0.66 mg/kg/wk | No concerns | Some concerns | No concerns | Some concerns | Major concerns | No concerns | Low | Reporting bias, Imprecision, Heterogeneity |
| PEG-rhGH 0.12 mg/kg/wk:YPEG-rhGH 0.1 mg/kg/wk | No concerns | Some concerns | No concerns | Major concerns | Major concerns | No concerns | Very low | Reporting bias, Imprecision, Heterogeneity |
| PEG-rhGH 0.12 mg/kg/wk:YPEG-rhGH 0.12 mg/kg/wk | No concerns | Some concerns | No concerns | Major concerns | Major concerns | No concerns | Very low | Reporting bias, Imprecision, Heterogeneity |
| PEG-rhGH 0.12 mg/kg/wk:YPEG-rhGH 0.14 mg/kg/wk | No concerns | Some concerns | No concerns | Major concerns | Major concerns | No concerns | Very low | Reporting bias, Imprecision, Heterogeneity |
| PEG-rhGH 0.12 mg/kg/wk:Somapacitan 0.08 mg/kg/wk | Some concerns | Some concerns | No concerns | Major concerns | Major concerns | No concerns | Very low | Within-study bias, Reporting bias, Imprecision, Heterogeneity |
| PEG-rhGH 0.12 mg/kg/wk:Somapacitan 0.04mg/kg/wk | Some concerns | Some concerns | No concerns | Some concerns | Major concerns | No concerns | Low | Within-study bias, Reporting bias, Imprecision, Heterogeneity |
| PEG-rhGH 0.12 mg/kg/wk:Somapacitan 0.04/0.16 mg/kg/wk | Some concerns | Some concerns | No concerns | Major concerns | Major concerns | No concerns | Very low | Within-study bias, Reporting bias, Imprecision, Heterogeneity |
| PEG-rhGH 0.12 mg/kg/wk:Somapacitan 0.08/0.16 mg/kg/wk | Some concerns | Some concerns | No concerns | Major concerns | Major concerns | No concerns | Very low | Within-study bias, Reporting bias, Imprecision, Heterogeneity |
| Lonapegsomatropin 0.24 mg/kg/wk:PEG-rhGH 0.12 mg/kg/wk | No concerns | Some concerns | No concerns | Major concerns | Major concerns | No concerns | Very low | Reporting bias, Imprecision, Heterogeneity |
| Somapacitan 0.16 mg/kg/wk:Somatrogon 0.25/0.48/0.66 mg/kg/wk | Some concerns | Some concerns | No concerns | Some concerns | Major concerns | No concerns | Low | Within-study bias, Reporting bias, Imprecision, Heterogeneity |
| Somapacitan 0.16 mg/kg/wk:YPEG-rhGH 0.1 mg/kg/wk | Some concerns | Some concerns | Some concerns | Major concerns | Major concerns | No concerns | Very low | Within-study bias, Reporting bias, Indirectness, Imprecision, Heterogeneity |
| Somapacitan 0.16 mg/kg/wk:YPEG-rhGH 0.12 mg/kg/wk | Some concerns | Some concerns | Some concerns | Major concerns | Major concerns | No concerns | Very low | Within-study bias, Reporting bias, Indirectness, Imprecision, Heterogeneity |
| Somapacitan 0.16 mg/kg/wk:YPEG-rhGH 0.14 mg/kg/wk | Some concerns | Some concerns | Some concerns | Major concerns | Major concerns | No concerns | Very low | Within-study bias, Reporting bias, Indirectness, Imprecision, Heterogeneity |
| Somapacitan 0.08 mg/kg/wk:Somapacitan 0.16 mg/kg/wk | Some concerns | Some concerns | Some concerns | Major concerns | Major concerns | No concerns | Very low | Within-study bias, Reporting bias, Indirectness, Imprecision, Heterogeneity |
| Somapacitan 0.04mg/kg/wk:Somapacitan 0.16 mg/kg/wk | Some concerns | Some concerns | Some concerns | Major concerns | Major concerns | No concerns | Very low | Within-study bias, Reporting bias, Indirectness, Imprecision, Heterogeneity |
| Somapacitan 0.04/0.16 mg/kg/wk:Somapacitan 0.16 mg/kg/wk | Some concerns | Some concerns | No concerns | Major concerns | Major concerns | No concerns | Very low | Within-study bias, Reporting bias, Imprecision, Heterogeneity |
| Somapacitan 0.08/0.16 mg/kg/wk:Somapacitan 0.16 mg/kg/wk | Some concerns | Some concerns | No concerns | Major concerns | Major concerns | No concerns | Very low | Within-study bias, Reporting bias, Imprecision, Heterogeneity |
| Lonapegsomatropin 0.24 mg/kg/wk:Somapacitan 0.16 mg/kg/wk | Some concerns | Some concerns | No concerns | Major concerns | Major concerns | No concerns | Very low | Within-study bias, Reporting bias, Imprecision, Heterogeneity |
| Somatrogon 0.25/0.48/0.66 mg/kg/wk:YPEG-rhGH 0.1 mg/kg/wk | No concerns | Some concerns | No concerns | Some concerns | Major concerns | No concerns | Low | Reporting bias, Imprecision, Heterogeneity |
| Somatrogon 0.25/0.48/0.66 mg/kg/wk:YPEG-rhGH 0.12 mg/kg/wk | No concerns | Some concerns | No concerns | Major concerns | Major concerns | No concerns | Very low | Reporting bias, Imprecision, Heterogeneity |
| Somatrogon 0.25/0.48/0.66 mg/kg/wk:YPEG-rhGH 0.14 mg/kg/wk | No concerns | Some concerns | No concerns | Major concerns | Major concerns | No concerns | Very low | Reporting bias, Imprecision, Heterogeneity |
| Somapacitan 0.08 mg/kg/wk:Somatrogon 0.25/0.48/0.66 mg/kg/wk | Some concerns | Some concerns | No concerns | Some concerns | Major concerns | No concerns | Low | Within-study bias, Reporting bias, Imprecision, Heterogeneity |
| Somapacitan 0.04mg/kg/wk:Somatrogon 0.25/0.48/0.66 mg/kg/wk | Some concerns | Some concerns | No concerns | Major concerns | Major concerns | No concerns | Very low | Within-study bias, Reporting bias, Imprecision, Heterogeneity |
| Somapacitan 0.04/0.16 mg/kg/wk:Somatrogon 0.25/0.48/0.66 mg/kg/wk | Some concerns | Some concerns | No concerns | Major concerns | Major concerns | No concerns | Very low | Within-study bias, Reporting bias, Imprecision, Heterogeneity |
| Somapacitan 0.08/0.16 mg/kg/wk:Somatrogon 0.25/0.48/0.66 mg/kg/wk | Some concerns | Some concerns | No concerns | Some concerns | Major concerns | No concerns | Low | Within-study bias, Reporting bias, Imprecision, Heterogeneity |
| Lonapegsomatropin 0.24 mg/kg/wk:Somatrogon 0.25/0.48/0.66 mg/kg/wk | No concerns | Some concerns | No concerns | Some concerns | Major concerns | No concerns | Low | Reporting bias, Imprecision, Heterogeneity |
| YPEG-rhGH 0.1 mg/kg/wk:YPEG-rhGH 0.12 mg/kg/wk | No concerns | Some concerns | Some concerns | Major concerns | Major concerns | No concerns | Very low | Reporting bias, Indirectness, Imprecision, Heterogeneity |
| YPEG-rhGH 0.1 mg/kg/wk:YPEG-rhGH 0.14 mg/kg/wk | No concerns | Some concerns | Some concerns | Some concerns | Major concerns | No concerns | Low | Reporting bias, Indirectness, Imprecision, Heterogeneity |
| Somapacitan 0.08 mg/kg/wk:YPEG-rhGH 0.1 mg/kg/wk | Some concerns | Some concerns | Some concerns | Major concerns | Major concerns | No concerns | Very low | Within-study bias, Reporting bias, Indirectness, Imprecision, Heterogeneity |
| Somapacitan 0.04mg/kg/wk:YPEG-rhGH 0.1 mg/kg/wk | Some concerns | Some concerns | Some concerns | Some concerns | Major concerns | No concerns | Low | Within-study bias, Reporting bias, Indirectness, Imprecision, Heterogeneity |
| Somapacitan 0.04/0.16 mg/kg/wk:YPEG-rhGH 0.1 mg/kg/wk | Some concerns | Some concerns | No concerns | Some concerns | Major concerns | No concerns | Low | Within-study bias, Reporting bias, Imprecision, Heterogeneity |
| Somapacitan 0.08/0.16 mg/kg/wk:YPEG-rhGH 0.1 mg/kg/wk | Some concerns | Some concerns | No concerns | Major concerns | Major concerns | No concerns | Very low | Within-study bias, Reporting bias, Imprecision, Heterogeneity |
| Lonapegsomatropin 0.24 mg/kg/wk:YPEG-rhGH 0.1 mg/kg/wk | No concerns | Some concerns | No concerns | Major concerns | Major concerns | No concerns | Very low | Reporting bias, Imprecision, Heterogeneity |
| YPEG-rhGH 0.12 mg/kg/wk:YPEG-rhGH 0.14 mg/kg/wk | No concerns | Some concerns | Some concerns | Major concerns | Major concerns | No concerns | Very low | Reporting bias, Indirectness, Imprecision, Heterogeneity |
| Somapacitan 0.08 mg/kg/wk:YPEG-rhGH 0.12 mg/kg/wk | Some concerns | Some concerns | Some concerns | Major concerns | Major concerns | No concerns | Very low | Within-study bias, Reporting bias, Indirectness, Imprecision, Heterogeneity |
| Somapacitan 0.04mg/kg/wk:YPEG-rhGH 0.12 mg/kg/wk | Some concerns | Some concerns | Some concerns | Major concerns | Major concerns | No concerns | Very low | Within-study bias, Reporting bias, Indirectness, Imprecision, Heterogeneity |
| Somapacitan 0.04/0.16 mg/kg/wk:YPEG-rhGH 0.12 mg/kg/wk | Some concerns | Some concerns | No concerns | Major concerns | Major concerns | No concerns | Very low | Within-study bias, Reporting bias, Imprecision, Heterogeneity |
| Somapacitan 0.08/0.16 mg/kg/wk:YPEG-rhGH 0.12 mg/kg/wk | Some concerns | Some concerns | No concerns | Major concerns | Major concerns | No concerns | Very low | Within-study bias, Reporting bias, Imprecision, Heterogeneity |
| Lonapegsomatropin 0.24 mg/kg/wk:YPEG-rhGH 0.12 mg/kg/wk | No concerns | Some concerns | No concerns | Major concerns | Major concerns | No concerns | Very low | Reporting bias, Imprecision, Heterogeneity |
| Somapacitan 0.08 mg/kg/wk:YPEG-rhGH 0.14 mg/kg/wk | Some concerns | Some concerns | Some concerns | Major concerns | Major concerns | No concerns | Very low | Within-study bias, Reporting bias, Indirectness, Imprecision, Heterogeneity |
| Somapacitan 0.04mg/kg/wk:YPEG-rhGH 0.14 mg/kg/wk | Some concerns | Some concerns | Some concerns | Major concerns | Major concerns | No concerns | Very low | Within-study bias, Reporting bias, Indirectness, Imprecision, Heterogeneity |
| Somapacitan 0.04/0.16 mg/kg/wk:YPEG-rhGH 0.14 mg/kg/wk | Some concerns | Some concerns | No concerns | Major concerns | Major concerns | No concerns | Very low | Within-study bias, Reporting bias, Imprecision, Heterogeneity |
| Somapacitan 0.08/0.16 mg/kg/wk:YPEG-rhGH 0.14 mg/kg/wk | Some concerns | Some concerns | No concerns | Major concerns | Major concerns | No concerns | Very low | Within-study bias, Reporting bias, Imprecision, Heterogeneity |
| Lonapegsomatropin 0.24 mg/kg/wk:YPEG-rhGH 0.14 mg/kg/wk | No concerns | Some concerns | No concerns | Major concerns | Major concerns | No concerns | Very low | Reporting bias, Imprecision, Heterogeneity |
| Somapacitan 0.04mg/kg/wk:Somapacitan 0.08 mg/kg/wk | Major concerns | Some concerns | Some concerns | Major concerns | Major concerns | No concerns | Very low | Within-study bias, Reporting bias, Indirectness, Imprecision, Heterogeneity |
| Somapacitan 0.04/0.16 mg/kg/wk:Somapacitan 0.08 mg/kg/wk | Major concerns | Some concerns | No concerns | Major concerns | Major concerns | No concerns | Very low | Within-study bias, Reporting bias, Imprecision, Heterogeneity |
| Somapacitan 0.08 mg/kg/wk:Somapacitan 0.08/0.16 mg/kg/wk | Major concerns | Some concerns | No concerns | Major concerns | Major concerns | No concerns | Very low | Within-study bias, Reporting bias, Imprecision, Heterogeneity |
| Lonapegsomatropin 0.24 mg/kg/wk:Somapacitan 0.08 mg/kg/wk | Some concerns | Some concerns | No concerns | Major concerns | Major concerns | No concerns | Very low | Within-study bias, Reporting bias, Imprecision, Heterogeneity |
| Somapacitan 0.04/0.16 mg/kg/wk:Somapacitan 0.04mg/kg/wk | Major concerns | Some concerns | No concerns | Major concerns | Major concerns | No concerns | Very low | Within-study bias, Reporting bias, Imprecision, Heterogeneity |
| Somapacitan 0.04mg/kg/wk:Somapacitan 0.08/0.16 mg/kg/wk | Major concerns | Some concerns | No concerns | Major concerns | Major concerns | No concerns | Very low | Within-study bias, Reporting bias, Imprecision, Heterogeneity |
| Lonapegsomatropin 0.24 mg/kg/wk:Somapacitan 0.04mg/kg/wk | Some concerns | Some concerns | No concerns | Some concerns | Major concerns | No concerns | Low | Within-study bias, Reporting bias, Imprecision, Heterogeneity |
| Somapacitan 0.04/0.16 mg/kg/wk:Somapacitan 0.08/0.16 mg/kg/wk | Major concerns | Some concerns | No concerns | Major concerns | Major concerns | No concerns | Very low | Within-study bias, Reporting bias, Imprecision, Heterogeneity |
| Lonapegsomatropin 0.24 mg/kg/wk:Somapacitan 0.04/0.16 mg/kg/wk | Some concerns | Some concerns | No concerns | Some concerns | Major concerns | No concerns | Low | Within-study bias, Reporting bias, Imprecision, Heterogeneity |
| Lonapegsomatropin 0.24 mg/kg/wk:Somapacitan 0.08/0.16 mg/kg/wk | Some concerns | Some concerns | No concerns | Major concerns | Major concerns | No concerns | Very low | Within-study bias, Reporting bias, Imprecision, Heterogeneity |

S8.2 Certainty of evidence for Height SDS

| Comparison | Within_Study_Bias | Reporting_Bias | Indirectness | Imprecision | Heterogeneity | Incoherence | Overall_Confidence | Reason_for_downgrade |
| --- | --- | --- | --- | --- | --- | --- | --- | --- |
| Daily rhGH:PEG-rhGH 0.1 mg/kg/wk | No concerns | Some concerns | No concerns | Major concerns | No concerns | No concerns | Low | Reporting bias, Imprecision |
| Daily rhGH:PEG-rhGH 0.2 mg/kg/wk | No concerns | Some concerns | No concerns | No concerns | No concerns | No concerns | Moderate | Reporting bias |
| Daily rhGH:TransCon GH 0.14 mg/kg/wk | No concerns | Some concerns | Some concerns | No concerns | No concerns | No concerns | Moderate | Reporting bias, Indirectness |
| Daily rhGH:TransCon GH 0.21 mg/kg/wk | No concerns | Some concerns | Some concerns | No concerns | No concerns | No concerns | Moderate | Reporting bias, Indirectness |
| Daily rhGH:TransCon GH 0.30 mg/kg/wk | No concerns | Some concerns | Some concerns | No concerns | No concerns | No concerns | Moderate | Reporting bias, Indirectness |
| Daily rhGH:Somatrogon 0.66 mg/kg/wk | No concerns | Some concerns | No concerns | No concerns | No concerns | No concerns | Moderate | Reporting bias |
| Daily rhGH:Somapacitan 0.16 mg/kg/wk | Some concerns | Some concerns | Some concerns | No concerns | No concerns | No concerns | Low | Within-study bias, Reporting bias, Indirectness |
| Daily rhGH:YPEG-rhGH 0.1 mg/kg/wk | No concerns | Some concerns | Some concerns | Major concerns | No concerns | No concerns | Low | Reporting bias, Indirectness, Imprecision |
| Daily rhGH:YPEG-rhGH 0.12 mg/kg/wk | No concerns | Some concerns | Some concerns | No concerns | No concerns | No concerns | Moderate | Reporting bias, Indirectness |
| Daily rhGH:YPEG-rhGH 0.14 mg/kg/wk | No concerns | Some concerns | No concerns | No concerns | No concerns | No concerns | Moderate | Reporting bias |
| Daily rhGH:Somapacitan 0.04/0.16 mg/kg/wk | Major concerns | Some concerns | No concerns | Major concerns | No concerns | No concerns | Very low | Within-study bias, Reporting bias, Imprecision |
| Daily rhGH:Somapacitan 0.08/0.16 mg/kg/wk | Major concerns | Some concerns | No concerns | No concerns | No concerns | No concerns | Low | Within-study bias, Reporting bias |
| Daily rhGH:Lonapegsomatropin 0.24 mg/kg/wk | Some concerns | Some concerns | No concerns | No concerns | No concerns | No concerns | Moderate | Within-study bias, Reporting bias |
| PEG-rhGH 0.1 mg/kg/wk:PEG-rhGH 0.2 mg/kg/wk | No concerns | Some concerns | No concerns | No concerns | No concerns | No concerns | Moderate | Reporting bias |
| PEG-rhGH 0.1 mg/kg/wk:TransCon GH 0.14 mg/kg/wk | No concerns | Some concerns | No concerns | No concerns | No concerns | No concerns | Moderate | Reporting bias |
| PEG-rhGH 0.1 mg/kg/wk:TransCon GH 0.21 mg/kg/wk | No concerns | Some concerns | No concerns | No concerns | No concerns | No concerns | Moderate | Reporting bias |
| PEG-rhGH 0.1 mg/kg/wk:TransCon GH 0.30 mg/kg/wk | No concerns | Some concerns | No concerns | No concerns | No concerns | No concerns | Moderate | Reporting bias |
| PEG-rhGH 0.1 mg/kg/wk:Somatrogon 0.66 mg/kg/wk | No concerns | Some concerns | No concerns | No concerns | No concerns | No concerns | Moderate | Reporting bias |
| PEG-rhGH 0.1 mg/kg/wk:Somapacitan 0.16 mg/kg/wk | Some concerns | Some concerns | No concerns | No concerns | No concerns | No concerns | Moderate | Within-study bias, Reporting bias |
| PEG-rhGH 0.1 mg/kg/wk:YPEG-rhGH 0.1 mg/kg/wk | No concerns | Some concerns | No concerns | Some concerns | No concerns | No concerns | Moderate | Reporting bias, Imprecision |
| PEG-rhGH 0.1 mg/kg/wk:YPEG-rhGH 0.12 mg/kg/wk | No concerns | Some concerns | No concerns | No concerns | No concerns | No concerns | Moderate | Reporting bias |
| PEG-rhGH 0.1 mg/kg/wk:YPEG-rhGH 0.14 mg/kg/wk | No concerns | Some concerns | No concerns | No concerns | No concerns | No concerns | Moderate | Reporting bias |
| PEG-rhGH 0.1 mg/kg/wk:Somapacitan 0.04/0.16 mg/kg/wk | Some concerns | Some concerns | No concerns | Major concerns | No concerns | No concerns | Low | Within-study bias, Reporting bias, Imprecision |
| PEG-rhGH 0.1 mg/kg/wk:Somapacitan 0.08/0.16 mg/kg/wk | Some concerns | Some concerns | No concerns | Some concerns | No concerns | No concerns | Low | Within-study bias, Reporting bias, Imprecision |
| Lonapegsomatropin 0.24 mg/kg/wk:PEG-rhGH 0.1 mg/kg/wk | Some concerns | Some concerns | No concerns | Major concerns | No concerns | No concerns | Low | Within-study bias, Reporting bias, Imprecision |
| PEG-rhGH 0.2 mg/kg/wk:TransCon GH 0.14 mg/kg/wk | No concerns | Some concerns | No concerns | No concerns | No concerns | No concerns | Moderate | Reporting bias |
| PEG-rhGH 0.2 mg/kg/wk:TransCon GH 0.21 mg/kg/wk | No concerns | Some concerns | No concerns | No concerns | No concerns | No concerns | Moderate | Reporting bias |
| PEG-rhGH 0.2 mg/kg/wk:TransCon GH 0.30 mg/kg/wk | No concerns | Some concerns | No concerns | No concerns | No concerns | No concerns | Moderate | Reporting bias |
| PEG-rhGH 0.2 mg/kg/wk:Somatrogon 0.66 mg/kg/wk | No concerns | Some concerns | No concerns | No concerns | No concerns | No concerns | Moderate | Reporting bias |
| PEG-rhGH 0.2 mg/kg/wk:Somapacitan 0.16 mg/kg/wk | Some concerns | Some concerns | No concerns | No concerns | No concerns | No concerns | Moderate | Within-study bias, Reporting bias |
| PEG-rhGH 0.2 mg/kg/wk:YPEG-rhGH 0.1 mg/kg/wk | No concerns | Some concerns | No concerns | Major concerns | No concerns | No concerns | Low | Reporting bias, Imprecision |
| PEG-rhGH 0.2 mg/kg/wk:YPEG-rhGH 0.12 mg/kg/wk | No concerns | Some concerns | No concerns | No concerns | No concerns | No concerns | Moderate | Reporting bias |
| PEG-rhGH 0.2 mg/kg/wk:YPEG-rhGH 0.14 mg/kg/wk | No concerns | Some concerns | No concerns | No concerns | No concerns | No concerns | Moderate | Reporting bias |
| PEG-rhGH 0.2 mg/kg/wk:Somapacitan 0.04/0.16 mg/kg/wk | Some concerns | Some concerns | No concerns | Major concerns | No concerns | No concerns | Low | Within-study bias, Reporting bias, Imprecision |
| PEG-rhGH 0.2 mg/kg/wk:Somapacitan 0.08/0.16 mg/kg/wk | Some concerns | Some concerns | No concerns | No concerns | No concerns | No concerns | Moderate | Within-study bias, Reporting bias |
| Lonapegsomatropin 0.24 mg/kg/wk:PEG-rhGH 0.2 mg/kg/wk | No concerns | Some concerns | No concerns | No concerns | No concerns | No concerns | Moderate | Reporting bias |
| TransCon GH 0.14 mg/kg/wk:TransCon GH 0.21 mg/kg/wk | No concerns | Some concerns | Some concerns | No concerns | No concerns | No concerns | Moderate | Reporting bias, Indirectness |
| TransCon GH 0.14 mg/kg/wk:TransCon GH 0.30 mg/kg/wk | No concerns | Some concerns | Some concerns | No concerns | No concerns | No concerns | Moderate | Reporting bias, Indirectness |
| Somatrogon 0.66 mg/kg/wk:TransCon GH 0.14 mg/kg/wk | No concerns | Some concerns | No concerns | No concerns | No concerns | No concerns | Moderate | Reporting bias |
| Somapacitan 0.16 mg/kg/wk:TransCon GH 0.14 mg/kg/wk | Some concerns | Some concerns | Some concerns | No concerns | No concerns | No concerns | Low | Within-study bias, Reporting bias, Indirectness |
| TransCon GH 0.14 mg/kg/wk:YPEG-rhGH 0.1 mg/kg/wk | No concerns | Some concerns | Some concerns | Major concerns | No concerns | No concerns | Low | Reporting bias, Indirectness, Imprecision |
| TransCon GH 0.14 mg/kg/wk:YPEG-rhGH 0.12 mg/kg/wk | No concerns | Some concerns | Some concerns | No concerns | No concerns | No concerns | Moderate | Reporting bias, Indirectness |
| TransCon GH 0.14 mg/kg/wk:YPEG-rhGH 0.14 mg/kg/wk | No concerns | Some concerns | Some concerns | No concerns | No concerns | No concerns | Moderate | Reporting bias, Indirectness |
| Somapacitan 0.04/0.16 mg/kg/wk:TransCon GH 0.14 mg/kg/wk | Some concerns | Some concerns | No concerns | Some concerns | No concerns | No concerns | Low | Within-study bias, Reporting bias, Imprecision |
| Somapacitan 0.08/0.16 mg/kg/wk:TransCon GH 0.14 mg/kg/wk | Some concerns | Some concerns | No concerns | Major concerns | No concerns | No concerns | Low | Within-study bias, Reporting bias, Imprecision |
| Lonapegsomatropin 0.24 mg/kg/wk:TransCon GH 0.14 mg/kg/wk | Some concerns | Some concerns | Some concerns | No concerns | No concerns | No concerns | Low | Within-study bias, Reporting bias, Indirectness |
| TransCon GH 0.21 mg/kg/wk:TransCon GH 0.30 mg/kg/wk | No concerns | Some concerns | Some concerns | No concerns | No concerns | No concerns | Moderate | Reporting bias, Indirectness |
| Somatrogon 0.66 mg/kg/wk:TransCon GH 0.21 mg/kg/wk | No concerns | Some concerns | No concerns | No concerns | No concerns | No concerns | Moderate | Reporting bias |
| Somapacitan 0.16 mg/kg/wk:TransCon GH 0.21 mg/kg/wk | Some concerns | Some concerns | Some concerns | No concerns | No concerns | No concerns | Low | Within-study bias, Reporting bias, Indirectness |
| TransCon GH 0.21 mg/kg/wk:YPEG-rhGH 0.1 mg/kg/wk | No concerns | Some concerns | Some concerns | Major concerns | No concerns | No concerns | Low | Reporting bias, Indirectness, Imprecision |
| TransCon GH 0.21 mg/kg/wk:YPEG-rhGH 0.12 mg/kg/wk | No concerns | Some concerns | Some concerns | No concerns | No concerns | No concerns | Moderate | Reporting bias, Indirectness |
| TransCon GH 0.21 mg/kg/wk:YPEG-rhGH 0.14 mg/kg/wk | No concerns | Some concerns | Some concerns | No concerns | No concerns | No concerns | Moderate | Reporting bias, Indirectness |
| Somapacitan 0.04/0.16 mg/kg/wk:TransCon GH 0.21 mg/kg/wk | Some concerns | Some concerns | No concerns | Some concerns | No concerns | No concerns | Low | Within-study bias, Reporting bias, Imprecision |
| Somapacitan 0.08/0.16 mg/kg/wk:TransCon GH 0.21 mg/kg/wk | Some concerns | Some concerns | No concerns | No concerns | No concerns | No concerns | Moderate | Within-study bias, Reporting bias |
| Lonapegsomatropin 0.24 mg/kg/wk:TransCon GH 0.21 mg/kg/wk | Some concerns | Some concerns | Some concerns | No concerns | No concerns | No concerns | Low | Within-study bias, Reporting bias, Indirectness |
| Somatrogon 0.66 mg/kg/wk:TransCon GH 0.30 mg/kg/wk | No concerns | Some concerns | No concerns | No concerns | No concerns | No concerns | Moderate | Reporting bias |
| Somapacitan 0.16 mg/kg/wk:TransCon GH 0.30 mg/kg/wk | Some concerns | Some concerns | Some concerns | No concerns | No concerns | No concerns | Low | Within-study bias, Reporting bias, Indirectness |
| TransCon GH 0.30 mg/kg/wk:YPEG-rhGH 0.1 mg/kg/wk | No concerns | Some concerns | Some concerns | Major concerns | No concerns | No concerns | Low | Reporting bias, Indirectness, Imprecision |
| TransCon GH 0.30 mg/kg/wk:YPEG-rhGH 0.12 mg/kg/wk | No concerns | Some concerns | Some concerns | Major concerns | No concerns | No concerns | Low | Reporting bias, Indirectness, Imprecision |
| TransCon GH 0.30 mg/kg/wk:YPEG-rhGH 0.14 mg/kg/wk | No concerns | Some concerns | Some concerns | No concerns | No concerns | No concerns | Moderate | Reporting bias, Indirectness |
| Somapacitan 0.04/0.16 mg/kg/wk:TransCon GH 0.30 mg/kg/wk | Some concerns | Some concerns | No concerns | Some concerns | No concerns | No concerns | Low | Within-study bias, Reporting bias, Imprecision |
| Somapacitan 0.08/0.16 mg/kg/wk:TransCon GH 0.30 mg/kg/wk | Some concerns | Some concerns | No concerns | No concerns | No concerns | No concerns | Moderate | Within-study bias, Reporting bias |
| Lonapegsomatropin 0.24 mg/kg/wk:TransCon GH 0.30 mg/kg/wk | Some concerns | Some concerns | Some concerns | No concerns | No concerns | No concerns | Low | Within-study bias, Reporting bias, Indirectness |
| Somapacitan 0.16 mg/kg/wk:Somatrogon 0.66 mg/kg/wk | Some concerns | Some concerns | No concerns | No concerns | No concerns | No concerns | Moderate | Within-study bias, Reporting bias |
| Somatrogon 0.66 mg/kg/wk:YPEG-rhGH 0.1 mg/kg/wk | No concerns | Some concerns | No concerns | Major concerns | No concerns | No concerns | Low | Reporting bias, Imprecision |
| Somatrogon 0.66 mg/kg/wk:YPEG-rhGH 0.12 mg/kg/wk | No concerns | Some concerns | No concerns | No concerns | No concerns | No concerns | Moderate | Reporting bias |
| Somatrogon 0.66 mg/kg/wk:YPEG-rhGH 0.14 mg/kg/wk | No concerns | Some concerns | No concerns | No concerns | No concerns | No concerns | Moderate | Reporting bias |
| Somapacitan 0.04/0.16 mg/kg/wk:Somatrogon 0.66 mg/kg/wk | Some concerns | Some concerns | No concerns | Some concerns | No concerns | No concerns | Low | Within-study bias, Reporting bias, Imprecision |
| Somapacitan 0.08/0.16 mg/kg/wk:Somatrogon 0.66 mg/kg/wk | Some concerns | Some concerns | No concerns | No concerns | No concerns | No concerns | Moderate | Within-study bias, Reporting bias |
| Lonapegsomatropin 0.24 mg/kg/wk:Somatrogon 0.66 mg/kg/wk | Some concerns | Some concerns | No concerns | No concerns | No concerns | No concerns | Moderate | Within-study bias, Reporting bias |
| Somapacitan 0.16 mg/kg/wk:YPEG-rhGH 0.1 mg/kg/wk | Some concerns | Some concerns | Some concerns | Major concerns | No concerns | No concerns | Low | Within-study bias, Reporting bias, Indirectness, Imprecision |
| Somapacitan 0.16 mg/kg/wk:YPEG-rhGH 0.12 mg/kg/wk | Some concerns | Some concerns | Some concerns | No concerns | No concerns | No concerns | Low | Within-study bias, Reporting bias, Indirectness |
| Somapacitan 0.16 mg/kg/wk:YPEG-rhGH 0.14 mg/kg/wk | Some concerns | Some concerns | No concerns | No concerns | No concerns | No concerns | Moderate | Within-study bias, Reporting bias |
| Somapacitan 0.04/0.16 mg/kg/wk:Somapacitan 0.16 mg/kg/wk | Some concerns | Some concerns | No concerns | Some concerns | No concerns | No concerns | Low | Within-study bias, Reporting bias, Imprecision |
| Somapacitan 0.08/0.16 mg/kg/wk:Somapacitan 0.16 mg/kg/wk | Some concerns | Some concerns | No concerns | No concerns | No concerns | No concerns | Moderate | Within-study bias, Reporting bias |
| Lonapegsomatropin 0.24 mg/kg/wk:Somapacitan 0.16 mg/kg/wk | Some concerns | Some concerns | No concerns | No concerns | No concerns | No concerns | Moderate | Within-study bias, Reporting bias |
| YPEG-rhGH 0.1 mg/kg/wk:YPEG-rhGH 0.12 mg/kg/wk | No concerns | Some concerns | Some concerns | No concerns | No concerns | No concerns | Moderate | Reporting bias, Indirectness |
| YPEG-rhGH 0.1 mg/kg/wk:YPEG-rhGH 0.14 mg/kg/wk | No concerns | Some concerns | Some concerns | No concerns | No concerns | No concerns | Moderate | Reporting bias, Indirectness |
| Somapacitan 0.04/0.16 mg/kg/wk:YPEG-rhGH 0.1 mg/kg/wk | Some concerns | Some concerns | No concerns | Major concerns | No concerns | No concerns | Low | Within-study bias, Reporting bias, Imprecision |
| Somapacitan 0.08/0.16 mg/kg/wk:YPEG-rhGH 0.1 mg/kg/wk | Some concerns | Some concerns | No concerns | Major concerns | No concerns | No concerns | Low | Within-study bias, Reporting bias, Imprecision |
| Lonapegsomatropin 0.24 mg/kg/wk:YPEG-rhGH 0.1 mg/kg/wk | Some concerns | Some concerns | Some concerns | Major concerns | No concerns | No concerns | Low | Within-study bias, Reporting bias, Indirectness, Imprecision |
| YPEG-rhGH 0.12 mg/kg/wk:YPEG-rhGH 0.14 mg/kg/wk | No concerns | Some concerns | Some concerns | No concerns | No concerns | No concerns | Moderate | Reporting bias, Indirectness |
| Somapacitan 0.04/0.16 mg/kg/wk:YPEG-rhGH 0.12 mg/kg/wk | Some concerns | Some concerns | No concerns | Some concerns | No concerns | No concerns | Low | Within-study bias, Reporting bias, Imprecision |
| Somapacitan 0.08/0.16 mg/kg/wk:YPEG-rhGH 0.12 mg/kg/wk | Some concerns | Some concerns | No concerns | Major concerns | No concerns | No concerns | Low | Within-study bias, Reporting bias, Imprecision |
| Lonapegsomatropin 0.24 mg/kg/wk:YPEG-rhGH 0.12 mg/kg/wk | Some concerns | Some concerns | Some concerns | No concerns | No concerns | No concerns | Low | Within-study bias, Reporting bias, Indirectness |
| Somapacitan 0.04/0.16 mg/kg/wk:YPEG-rhGH 0.14 mg/kg/wk | Some concerns | Some concerns | No concerns | Some concerns | No concerns | No concerns | Low | Within-study bias, Reporting bias, Imprecision |
| Somapacitan 0.08/0.16 mg/kg/wk:YPEG-rhGH 0.14 mg/kg/wk | Some concerns | Some concerns | No concerns | Major concerns | No concerns | No concerns | Low | Within-study bias, Reporting bias, Imprecision |
| Lonapegsomatropin 0.24 mg/kg/wk:YPEG-rhGH 0.14 mg/kg/wk | No concerns | Some concerns | No concerns | No concerns | No concerns | No concerns | Moderate | Reporting bias |
| Somapacitan 0.04/0.16 mg/kg/wk:Somapacitan 0.08/0.16 mg/kg/wk | Major concerns | Some concerns | No concerns | Some concerns | No concerns | No concerns | Low | Within-study bias, Reporting bias, Imprecision |
| Lonapegsomatropin 0.24 mg/kg/wk:Somapacitan 0.04/0.16 mg/kg/wk | Some concerns | Some concerns | No concerns | Major concerns | No concerns | No concerns | Low | Within-study bias, Reporting bias, Imprecision |
| Lonapegsomatropin 0.24 mg/kg/wk:Somapacitan 0.08/0.16 mg/kg/wk | Some concerns | Some concerns | No concerns | No concerns | No concerns | No concerns | Moderate | Within-study bias, Reporting bias |

S8.3 Certainty of evidence for Treatment discontinuation

| Comparison | Within_Study_Bias | Reporting_Bias | Indirectness | Imprecision | Heterogeneity | Incoherence | Overall_Confidence | Reason_for_downgrade |
| --- | --- | --- | --- | --- | --- | --- | --- | --- |
| Daily rhGH:PEG-rhGH 0.1 mg/kg/wk | No concerns | Some concerns | No concerns | Major concerns | Major concerns | No concerns | Very low | Reporting bias, Imprecision, Heterogeneity |
| Daily rhGH:PEG-rhGH 0.2 mg/kg/wk | No concerns | Some concerns | No concerns | Major concerns | Major concerns | No concerns | Very low | Reporting bias, Imprecision, Heterogeneity |
| Daily rhGH:TransCon GH 0.14 mg/kg/wk | No concerns | Some concerns | Some concerns | Major concerns | Major concerns | No concerns | Very low | Reporting bias, Indirectness, Imprecision, Heterogeneity |
| Daily rhGH:TransCon GH 0.21 mg/kg/wk | No concerns | Some concerns | Some concerns | Major concerns | Major concerns | No concerns | Very low | Reporting bias, Indirectness, Imprecision, Heterogeneity |
| Daily rhGH:TransCon GH 0.30 mg/kg/wk | No concerns | Some concerns | Some concerns | Major concerns | Major concerns | No concerns | Very low | Reporting bias, Indirectness, Imprecision, Heterogeneity |
| Daily rhGH:Somatrogon 0.66 mg/kg/wk | No concerns | Some concerns | No concerns | Major concerns | Major concerns | No concerns | Very low | Reporting bias, Imprecision, Heterogeneity |
| Daily rhGH:YPEG-rhGH 0.1 mg/kg/wk | No concerns | Some concerns | Some concerns | Some concerns | Major concerns | No concerns | Low | Reporting bias, Indirectness, Imprecision, Heterogeneity |
| Daily rhGH:YPEG-rhGH 0.12 mg/kg/wk | No concerns | Some concerns | Some concerns | Some concerns | Major concerns | No concerns | Low | Reporting bias, Indirectness, Imprecision, Heterogeneity |
| Daily rhGH:YPEG-rhGH 0.14 mg/kg/wk | No concerns | Some concerns | No concerns | Some concerns | Major concerns | No concerns | Low | Reporting bias, Imprecision, Heterogeneity |
| Daily rhGH:Somapacitan 0.16 mg/kg/wk | Some concerns | Some concerns | No concerns | Major concerns | Major concerns | No concerns | Very low | Within-study bias, Reporting bias, Imprecision, Heterogeneity |
| Daily rhGH:Lonapegsomatropin 0.24 mg/kg/wk | No concerns | Some concerns | No concerns | Major concerns | Major concerns | No concerns | Very low | Reporting bias, Imprecision, Heterogeneity |
| PEG-rhGH 0.1 mg/kg/wk:PEG-rhGH 0.2 mg/kg/wk | No concerns | Some concerns | No concerns | Major concerns | Major concerns | No concerns | Very low | Reporting bias, Imprecision, Heterogeneity |
| PEG-rhGH 0.1 mg/kg/wk:TransCon GH 0.14 mg/kg/wk | No concerns | Some concerns | No concerns | Major concerns | Major concerns | No concerns | Very low | Reporting bias, Imprecision, Heterogeneity |
| PEG-rhGH 0.1 mg/kg/wk:TransCon GH 0.21 mg/kg/wk | No concerns | Some concerns | No concerns | Major concerns | Major concerns | No concerns | Very low | Reporting bias, Imprecision, Heterogeneity |
| PEG-rhGH 0.1 mg/kg/wk:TransCon GH 0.30 mg/kg/wk | No concerns | Some concerns | No concerns | Major concerns | Major concerns | No concerns | Very low | Reporting bias, Imprecision, Heterogeneity |
| PEG-rhGH 0.1 mg/kg/wk:Somatrogon 0.66 mg/kg/wk | No concerns | Some concerns | No concerns | Major concerns | Major concerns | No concerns | Very low | Reporting bias, Imprecision, Heterogeneity |
| PEG-rhGH 0.1 mg/kg/wk:YPEG-rhGH 0.1 mg/kg/wk | No concerns | Some concerns | No concerns | Major concerns | Major concerns | No concerns | Very low | Reporting bias, Imprecision, Heterogeneity |
| PEG-rhGH 0.1 mg/kg/wk:YPEG-rhGH 0.12 mg/kg/wk | No concerns | Some concerns | No concerns | Major concerns | Major concerns | No concerns | Very low | Reporting bias, Imprecision, Heterogeneity |
| PEG-rhGH 0.1 mg/kg/wk:YPEG-rhGH 0.14 mg/kg/wk | No concerns | Some concerns | No concerns | Major concerns | Major concerns | No concerns | Very low | Reporting bias, Imprecision, Heterogeneity |
| PEG-rhGH 0.1 mg/kg/wk:Somapacitan 0.16 mg/kg/wk | No concerns | Some concerns | No concerns | Major concerns | Major concerns | No concerns | Very low | Reporting bias, Imprecision, Heterogeneity |
| Lonapegsomatropin 0.24 mg/kg/wk:PEG-rhGH 0.1 mg/kg/wk | No concerns | Some concerns | No concerns | Major concerns | Major concerns | No concerns | Very low | Reporting bias, Imprecision, Heterogeneity |
| PEG-rhGH 0.2 mg/kg/wk:TransCon GH 0.14 mg/kg/wk | No concerns | Some concerns | No concerns | Major concerns | Major concerns | No concerns | Very low | Reporting bias, Imprecision, Heterogeneity |
| PEG-rhGH 0.2 mg/kg/wk:TransCon GH 0.21 mg/kg/wk | No concerns | Some concerns | No concerns | Major concerns | Major concerns | No concerns | Very low | Reporting bias, Imprecision, Heterogeneity |
| PEG-rhGH 0.2 mg/kg/wk:TransCon GH 0.30 mg/kg/wk | No concerns | Some concerns | No concerns | Major concerns | Major concerns | No concerns | Very low | Reporting bias, Imprecision, Heterogeneity |
| PEG-rhGH 0.2 mg/kg/wk:Somatrogon 0.66 mg/kg/wk | No concerns | Some concerns | No concerns | Major concerns | Major concerns | No concerns | Very low | Reporting bias, Imprecision, Heterogeneity |
| PEG-rhGH 0.2 mg/kg/wk:YPEG-rhGH 0.1 mg/kg/wk | No concerns | Some concerns | No concerns | Major concerns | Major concerns | No concerns | Very low | Reporting bias, Imprecision, Heterogeneity |
| PEG-rhGH 0.2 mg/kg/wk:YPEG-rhGH 0.12 mg/kg/wk | No concerns | Some concerns | No concerns | Major concerns | Major concerns | No concerns | Very low | Reporting bias, Imprecision, Heterogeneity |
| PEG-rhGH 0.2 mg/kg/wk:YPEG-rhGH 0.14 mg/kg/wk | No concerns | Some concerns | No concerns | Major concerns | Major concerns | No concerns | Very low | Reporting bias, Imprecision, Heterogeneity |
| PEG-rhGH 0.2 mg/kg/wk:Somapacitan 0.16 mg/kg/wk | No concerns | Some concerns | No concerns | Major concerns | Major concerns | No concerns | Very low | Reporting bias, Imprecision, Heterogeneity |
| Lonapegsomatropin 0.24 mg/kg/wk:PEG-rhGH 0.2 mg/kg/wk | No concerns | Some concerns | No concerns | Major concerns | Major concerns | No concerns | Very low | Reporting bias, Imprecision, Heterogeneity |
| TransCon GH 0.14 mg/kg/wk:TransCon GH 0.21 mg/kg/wk | No concerns | Some concerns | Some concerns | Major concerns | Major concerns | No concerns | Very low | Reporting bias, Indirectness, Imprecision, Heterogeneity |
| TransCon GH 0.14 mg/kg/wk:TransCon GH 0.30 mg/kg/wk | No concerns | Some concerns | Some concerns | Major concerns | Major concerns | No concerns | Very low | Reporting bias, Indirectness, Imprecision, Heterogeneity |
| Somatrogon 0.66 mg/kg/wk:TransCon GH 0.14 mg/kg/wk | No concerns | Some concerns | No concerns | Major concerns | Major concerns | No concerns | Very low | Reporting bias, Imprecision, Heterogeneity |
| TransCon GH 0.14 mg/kg/wk:YPEG-rhGH 0.1 mg/kg/wk | No concerns | Some concerns | Some concerns | Major concerns | Major concerns | No concerns | Very low | Reporting bias, Indirectness, Imprecision, Heterogeneity |
| TransCon GH 0.14 mg/kg/wk:YPEG-rhGH 0.12 mg/kg/wk | No concerns | Some concerns | Some concerns | Major concerns | Major concerns | No concerns | Very low | Reporting bias, Indirectness, Imprecision, Heterogeneity |
| TransCon GH 0.14 mg/kg/wk:YPEG-rhGH 0.14 mg/kg/wk | No concerns | Some concerns | Some concerns | Major concerns | Major concerns | No concerns | Very low | Reporting bias, Indirectness, Imprecision, Heterogeneity |
| Somapacitan 0.16 mg/kg/wk:TransCon GH 0.14 mg/kg/wk | No concerns | Some concerns | No concerns | Major concerns | Major concerns | No concerns | Very low | Reporting bias, Imprecision, Heterogeneity |
| Lonapegsomatropin 0.24 mg/kg/wk:TransCon GH 0.14 mg/kg/wk | No concerns | Some concerns | No concerns | Major concerns | Major concerns | No concerns | Very low | Reporting bias, Imprecision, Heterogeneity |
| TransCon GH 0.21 mg/kg/wk:TransCon GH 0.30 mg/kg/wk | No concerns | Some concerns | Some concerns | Major concerns | Major concerns | No concerns | Very low | Reporting bias, Indirectness, Imprecision, Heterogeneity |
| Somatrogon 0.66 mg/kg/wk:TransCon GH 0.21 mg/kg/wk | No concerns | Some concerns | No concerns | Major concerns | Major concerns | No concerns | Very low | Reporting bias, Imprecision, Heterogeneity |
| TransCon GH 0.21 mg/kg/wk:YPEG-rhGH 0.1 mg/kg/wk | No concerns | Some concerns | Some concerns | Major concerns | Major concerns | No concerns | Very low | Reporting bias, Indirectness, Imprecision, Heterogeneity |
| TransCon GH 0.21 mg/kg/wk:YPEG-rhGH 0.12 mg/kg/wk | No concerns | Some concerns | Some concerns | Major concerns | Major concerns | No concerns | Very low | Reporting bias, Indirectness, Imprecision, Heterogeneity |
| TransCon GH 0.21 mg/kg/wk:YPEG-rhGH 0.14 mg/kg/wk | No concerns | Some concerns | Some concerns | Major concerns | Major concerns | No concerns | Very low | Reporting bias, Indirectness, Imprecision, Heterogeneity |
| Somapacitan 0.16 mg/kg/wk:TransCon GH 0.21 mg/kg/wk | No concerns | Some concerns | No concerns | Major concerns | Major concerns | No concerns | Very low | Reporting bias, Imprecision, Heterogeneity |
| Lonapegsomatropin 0.24 mg/kg/wk:TransCon GH 0.21 mg/kg/wk | No concerns | Some concerns | No concerns | Major concerns | Major concerns | No concerns | Very low | Reporting bias, Imprecision, Heterogeneity |
| Somatrogon 0.66 mg/kg/wk:TransCon GH 0.30 mg/kg/wk | No concerns | Some concerns | No concerns | Major concerns | Major concerns | No concerns | Very low | Reporting bias, Imprecision, Heterogeneity |
| TransCon GH 0.30 mg/kg/wk:YPEG-rhGH 0.1 mg/kg/wk | No concerns | Some concerns | Some concerns | Major concerns | Major concerns | No concerns | Very low | Reporting bias, Indirectness, Imprecision, Heterogeneity |
| TransCon GH 0.30 mg/kg/wk:YPEG-rhGH 0.12 mg/kg/wk | No concerns | Some concerns | Some concerns | Major concerns | Major concerns | No concerns | Very low | Reporting bias, Indirectness, Imprecision, Heterogeneity |
| TransCon GH 0.30 mg/kg/wk:YPEG-rhGH 0.14 mg/kg/wk | No concerns | Some concerns | Some concerns | Major concerns | Major concerns | No concerns | Very low | Reporting bias, Indirectness, Imprecision, Heterogeneity |
| Somapacitan 0.16 mg/kg/wk:TransCon GH 0.30 mg/kg/wk | No concerns | Some concerns | No concerns | Major concerns | Major concerns | No concerns | Very low | Reporting bias, Imprecision, Heterogeneity |
| Lonapegsomatropin 0.24 mg/kg/wk:TransCon GH 0.30 mg/kg/wk | No concerns | Some concerns | No concerns | Major concerns | Major concerns | No concerns | Very low | Reporting bias, Imprecision, Heterogeneity |
| Somatrogon 0.66 mg/kg/wk:YPEG-rhGH 0.1 mg/kg/wk | No concerns | Some concerns | No concerns | Major concerns | Major concerns | No concerns | Very low | Reporting bias, Imprecision, Heterogeneity |
| Somatrogon 0.66 mg/kg/wk:YPEG-rhGH 0.12 mg/kg/wk | No concerns | Some concerns | No concerns | Major concerns | Major concerns | No concerns | Very low | Reporting bias, Imprecision, Heterogeneity |
| Somatrogon 0.66 mg/kg/wk:YPEG-rhGH 0.14 mg/kg/wk | No concerns | Some concerns | No concerns | Major concerns | Major concerns | No concerns | Very low | Reporting bias, Imprecision, Heterogeneity |
| Somapacitan 0.16 mg/kg/wk:Somatrogon 0.66 mg/kg/wk | No concerns | Some concerns | No concerns | Major concerns | Major concerns | No concerns | Very low | Reporting bias, Imprecision, Heterogeneity |
| Lonapegsomatropin 0.24 mg/kg/wk:Somatrogon 0.66 mg/kg/wk | No concerns | Some concerns | No concerns | Major concerns | Major concerns | No concerns | Very low | Reporting bias, Imprecision, Heterogeneity |
| YPEG-rhGH 0.1 mg/kg/wk:YPEG-rhGH 0.12 mg/kg/wk | No concerns | Some concerns | Some concerns | Major concerns | Major concerns | No concerns | Very low | Reporting bias, Indirectness, Imprecision, Heterogeneity |
| YPEG-rhGH 0.1 mg/kg/wk:YPEG-rhGH 0.14 mg/kg/wk | No concerns | Some concerns | Some concerns | Major concerns | Major concerns | No concerns | Very low | Reporting bias, Indirectness, Imprecision, Heterogeneity |
| Somapacitan 0.16 mg/kg/wk:YPEG-rhGH 0.1 mg/kg/wk | No concerns | Some concerns | No concerns | Major concerns | Major concerns | No concerns | Very low | Reporting bias, Imprecision, Heterogeneity |
| Lonapegsomatropin 0.24 mg/kg/wk:YPEG-rhGH 0.1 mg/kg/wk | No concerns | Some concerns | No concerns | Major concerns | Major concerns | No concerns | Very low | Reporting bias, Imprecision, Heterogeneity |
| YPEG-rhGH 0.12 mg/kg/wk:YPEG-rhGH 0.14 mg/kg/wk | No concerns | Some concerns | Some concerns | Major concerns | Major concerns | No concerns | Very low | Reporting bias, Indirectness, Imprecision, Heterogeneity |
| Somapacitan 0.16 mg/kg/wk:YPEG-rhGH 0.12 mg/kg/wk | No concerns | Some concerns | No concerns | Major concerns | Major concerns | No concerns | Very low | Reporting bias, Imprecision, Heterogeneity |
| Lonapegsomatropin 0.24 mg/kg/wk:YPEG-rhGH 0.12 mg/kg/wk | No concerns | Some concerns | No concerns | Major concerns | Major concerns | No concerns | Very low | Reporting bias, Imprecision, Heterogeneity |
| Somapacitan 0.16 mg/kg/wk:YPEG-rhGH 0.14 mg/kg/wk | No concerns | Some concerns | No concerns | Major concerns | Major concerns | No concerns | Very low | Reporting bias, Imprecision, Heterogeneity |
| Lonapegsomatropin 0.24 mg/kg/wk:YPEG-rhGH 0.14 mg/kg/wk | No concerns | Some concerns | No concerns | Major concerns | Major concerns | No concerns | Very low | Reporting bias, Imprecision, Heterogeneity |
| Lonapegsomatropin 0.24 mg/kg/wk:Somapacitan 0.16 mg/kg/wk | No concerns | Some concerns | No concerns | Major concerns | Major concerns | No concerns | Very low | Reporting bias, Imprecision, Heterogeneity |

S8.4 Certainty of evidence for Injection site erythema

| Comparison | Within_Study_Bias | Reporting_Bias | Indirectness | Imprecision | Heterogeneity | Incoherence | Overall_Confidence | Reason_for_downgrade |
| --- | --- | --- | --- | --- | --- | --- | --- | --- |
| Daily rhGH:PEG-rhGH 0.1 mg/kg/wk | No concerns | Some concerns | No concerns | Major concerns | No concerns | No concerns | Low | Reporting bias, Imprecision |
| Daily rhGH:PEG-rhGH 0.2 mg/kg/wk | No concerns | Some concerns | No concerns | Major concerns | No concerns | No concerns | Low | Reporting bias, Imprecision |
| Daily rhGH:TransCon GH 0.14 mg/kg/wk | No concerns | Some concerns | Some concerns | No concerns | No concerns | No concerns | Moderate | Reporting bias, Indirectness |
| Daily rhGH:TransCon GH 0.21 mg/kg/wk | No concerns | Some concerns | Some concerns | No concerns | No concerns | No concerns | Moderate | Reporting bias, Indirectness |
| Daily rhGH:TransCon GH 0.30 mg/kg/wk | No concerns | Some concerns | Some concerns | No concerns | No concerns | No concerns | Moderate | Reporting bias, Indirectness |
| Daily rhGH:Somatrogon 0.66 mg/kg/wk | No concerns | Some concerns | No concerns | Major concerns | No concerns | No concerns | Low | Reporting bias, Imprecision |
| Daily rhGH:Somapacitan 0.16 mg/kg/wk | Some concerns | Some concerns | No concerns | Some concerns | No concerns | No concerns | Low | Within-study bias, Reporting bias, Imprecision |
| PEG-rhGH 0.1 mg/kg/wk:PEG-rhGH 0.2 mg/kg/wk | No concerns | Some concerns | No concerns | Major concerns | No concerns | No concerns | Low | Reporting bias, Imprecision |
| PEG-rhGH 0.1 mg/kg/wk:TransCon GH 0.14 mg/kg/wk | No concerns | Some concerns | No concerns | Major concerns | No concerns | No concerns | Low | Reporting bias, Imprecision |
| PEG-rhGH 0.1 mg/kg/wk:TransCon GH 0.21 mg/kg/wk | No concerns | Some concerns | No concerns | Major concerns | No concerns | No concerns | Low | Reporting bias, Imprecision |
| PEG-rhGH 0.1 mg/kg/wk:TransCon GH 0.30 mg/kg/wk | No concerns | Some concerns | No concerns | Major concerns | No concerns | No concerns | Low | Reporting bias, Imprecision |
| PEG-rhGH 0.1 mg/kg/wk:Somatrogon 0.66 mg/kg/wk | No concerns | Some concerns | No concerns | Major concerns | No concerns | No concerns | Low | Reporting bias, Imprecision |
| PEG-rhGH 0.1 mg/kg/wk:Somapacitan 0.16 mg/kg/wk | Some concerns | Some concerns | No concerns | Major concerns | No concerns | No concerns | Low | Within-study bias, Reporting bias, Imprecision |
| PEG-rhGH 0.2 mg/kg/wk:TransCon GH 0.14 mg/kg/wk | No concerns | Some concerns | No concerns | Major concerns | No concerns | No concerns | Low | Reporting bias, Imprecision |
| PEG-rhGH 0.2 mg/kg/wk:TransCon GH 0.21 mg/kg/wk | No concerns | Some concerns | No concerns | Major concerns | No concerns | No concerns | Low | Reporting bias, Imprecision |
| PEG-rhGH 0.2 mg/kg/wk:TransCon GH 0.30 mg/kg/wk | No concerns | Some concerns | No concerns | Major concerns | No concerns | No concerns | Low | Reporting bias, Imprecision |
| PEG-rhGH 0.2 mg/kg/wk:Somatrogon 0.66 mg/kg/wk | No concerns | Some concerns | No concerns | Major concerns | No concerns | No concerns | Low | Reporting bias, Imprecision |
| PEG-rhGH 0.2 mg/kg/wk:Somapacitan 0.16 mg/kg/wk | Some concerns | Some concerns | No concerns | Major concerns | No concerns | No concerns | Low | Within-study bias, Reporting bias, Imprecision |
| TransCon GH 0.14 mg/kg/wk:TransCon GH 0.21 mg/kg/wk | No concerns | Some concerns | Some concerns | Some concerns | No concerns | No concerns | Low | Reporting bias, Indirectness, Imprecision |
| TransCon GH 0.14 mg/kg/wk:TransCon GH 0.30 mg/kg/wk | No concerns | Some concerns | Some concerns | Some concerns | No concerns | No concerns | Low | Reporting bias, Indirectness, Imprecision |
| Somatrogon 0.66 mg/kg/wk:TransCon GH 0.14 mg/kg/wk | No concerns | Some concerns | No concerns | Some concerns | No concerns | No concerns | Moderate | Reporting bias, Imprecision |
| Somapacitan 0.16 mg/kg/wk:TransCon GH 0.14 mg/kg/wk | Some concerns | Some concerns | Some concerns | Major concerns | No concerns | No concerns | Low | Within-study bias, Reporting bias, Indirectness, Imprecision |
| TransCon GH 0.21 mg/kg/wk:TransCon GH 0.30 mg/kg/wk | No concerns | Some concerns | Some concerns | Major concerns | No concerns | No concerns | Low | Reporting bias, Indirectness, Imprecision |
| Somatrogon 0.66 mg/kg/wk:TransCon GH 0.21 mg/kg/wk | No concerns | Some concerns | No concerns | Some concerns | No concerns | No concerns | Moderate | Reporting bias, Imprecision |
| Somapacitan 0.16 mg/kg/wk:TransCon GH 0.21 mg/kg/wk | Some concerns | Some concerns | Some concerns | Major concerns | No concerns | No concerns | Low | Within-study bias, Reporting bias, Indirectness, Imprecision |
| Somatrogon 0.66 mg/kg/wk:TransCon GH 0.30 mg/kg/wk | No concerns | Some concerns | No concerns | Some concerns | No concerns | No concerns | Moderate | Reporting bias, Imprecision |
| Somapacitan 0.16 mg/kg/wk:TransCon GH 0.30 mg/kg/wk | Some concerns | Some concerns | Some concerns | Major concerns | No concerns | No concerns | Low | Within-study bias, Reporting bias, Indirectness, Imprecision |
| Somapacitan 0.16 mg/kg/wk:Somatrogon 0.66 mg/kg/wk | Some concerns | Some concerns | No concerns | Some concerns | No concerns | No concerns | Low | Within-study bias, Reporting bias, Imprecision |

S8.5 Certainty of evidence for Influenza

| Comparison | Within_Study_Bias | Reporting_Bias | Indirectness | Imprecision | Heterogeneity | Incoherence | Overall_Confidence | Reason_for_downgrade |
| --- | --- | --- | --- | --- | --- | --- | --- | --- |
| Daily rhGH:Somatrogon 0.25 mg/kg/wk | No concerns | Some concerns | No concerns | Major concerns | Some concerns | No concerns | Low | Reporting bias, Imprecision, Heterogeneity |
| Daily rhGH:Somatrogon 0.48 mg/kg/wk | No concerns | Some concerns | No concerns | Major concerns | Some concerns | No concerns | Low | Reporting bias, Imprecision, Heterogeneity |
| Daily rhGH:Somatrogon 0.66 mg/kg/wk | No concerns | Some concerns | No concerns | Major concerns | Some concerns | No concerns | Low | Reporting bias, Imprecision, Heterogeneity |
| Daily rhGH:Somapacitan 0.08 mg/kg/wk | Major concerns | Some concerns | Some concerns | Major concerns | Some concerns | No concerns | Very low | Within-study bias, Reporting bias, Indirectness, Imprecision, Heterogeneity |
| Daily rhGH:Somapacitan 0.16 mg/kg/wk | Major concerns | Some concerns | Some concerns | Major concerns | Some concerns | No concerns | Very low | Within-study bias, Reporting bias, Indirectness, Imprecision, Heterogeneity |
| Daily rhGH:Lonapegsomatropin 0.24 mg/kg/wk | No concerns | Some concerns | No concerns | Major concerns | Some concerns | No concerns | Low | Reporting bias, Imprecision, Heterogeneity |
| Somatrogon 0.25 mg/kg/wk:Somatrogon 0.48 mg/kg/wk | No concerns | Some concerns | No concerns | Major concerns | Some concerns | No concerns | Low | Reporting bias, Imprecision, Heterogeneity |
| Somatrogon 0.25 mg/kg/wk:Somatrogon 0.66 mg/kg/wk | No concerns | Some concerns | No concerns | Major concerns | Some concerns | No concerns | Low | Reporting bias, Imprecision, Heterogeneity |
| Somapacitan 0.08 mg/kg/wk:Somatrogon 0.25 mg/kg/wk | Some concerns | Some concerns | No concerns | Major concerns | Some concerns | No concerns | Low | Within-study bias, Reporting bias, Imprecision, Heterogeneity |
| Somapacitan 0.16 mg/kg/wk:Somatrogon 0.25 mg/kg/wk | Some concerns | Some concerns | No concerns | Major concerns | Some concerns | No concerns | Low | Within-study bias, Reporting bias, Imprecision, Heterogeneity |
| Lonapegsomatropin 0.24 mg/kg/wk:Somatrogon 0.25 mg/kg/wk | No concerns | Some concerns | No concerns | Major concerns | Some concerns | No concerns | Low | Reporting bias, Imprecision, Heterogeneity |
| Somatrogon 0.48 mg/kg/wk:Somatrogon 0.66 mg/kg/wk | No concerns | Some concerns | No concerns | Major concerns | Some concerns | No concerns | Low | Reporting bias, Imprecision, Heterogeneity |
| Somapacitan 0.08 mg/kg/wk:Somatrogon 0.48 mg/kg/wk | Some concerns | Some concerns | No concerns | Major concerns | Some concerns | No concerns | Low | Within-study bias, Reporting bias, Imprecision, Heterogeneity |
| Somapacitan 0.16 mg/kg/wk:Somatrogon 0.48 mg/kg/wk | Some concerns | Some concerns | No concerns | Major concerns | Some concerns | No concerns | Low | Within-study bias, Reporting bias, Imprecision, Heterogeneity |
| Lonapegsomatropin 0.24 mg/kg/wk:Somatrogon 0.48 mg/kg/wk | No concerns | Some concerns | No concerns | Major concerns | Some concerns | No concerns | Low | Reporting bias, Imprecision, Heterogeneity |
| Somapacitan 0.08 mg/kg/wk:Somatrogon 0.66 mg/kg/wk | Some concerns | Some concerns | No concerns | Major concerns | Some concerns | No concerns | Low | Within-study bias, Reporting bias, Imprecision, Heterogeneity |
| Somapacitan 0.16 mg/kg/wk:Somatrogon 0.66 mg/kg/wk | Some concerns | Some concerns | No concerns | Major concerns | Some concerns | No concerns | Low | Within-study bias, Reporting bias, Imprecision, Heterogeneity |
| Lonapegsomatropin 0.24 mg/kg/wk:Somatrogon 0.66 mg/kg/wk | No concerns | Some concerns | No concerns | Major concerns | Some concerns | No concerns | Low | Reporting bias, Imprecision, Heterogeneity |
| Somapacitan 0.08 mg/kg/wk:Somapacitan 0.16 mg/kg/wk | Major concerns | Some concerns | Some concerns | Major concerns | Some concerns | No concerns | Very low | Within-study bias, Reporting bias, Indirectness, Imprecision, Heterogeneity |
| Lonapegsomatropin 0.24 mg/kg/wk:Somapacitan 0.08 mg/kg/wk | Some concerns | Some concerns | No concerns | Major concerns | Some concerns | No concerns | Low | Within-study bias, Reporting bias, Imprecision, Heterogeneity |
| Lonapegsomatropin 0.24 mg/kg/wk:Somapacitan 0.16 mg/kg/wk | Some concerns | Some concerns | No concerns | Major concerns | Some concerns | No concerns | Low | Within-study bias, Reporting bias, Imprecision, Heterogeneity |

S8.6 Certainty of evidence for Headache

| Comparison | Within_Study_Bias | Reporting_Bias | Indirectness | Imprecision | Heterogeneity | Incoherence | Overall_Confidence | Reason_for_downgrade |
| --- | --- | --- | --- | --- | --- | --- | --- | --- |
| Daily rhGH:PEG-rhGH 0.1 mg/kg/wk | No concerns | Some concerns | No concerns | Major concerns | Some concerns | No concerns | Low | Reporting bias, Imprecision, Heterogeneity |
| Daily rhGH:PEG-rhGH 0.2 mg/kg/wk | No concerns | Some concerns | No concerns | Major concerns | Some concerns | No concerns | Low | Reporting bias, Imprecision, Heterogeneity |
| Daily rhGH:TransCon GH 0.30 mg/kg/wk | No concerns | Some concerns | Some concerns | Major concerns | Some concerns | No concerns | Low | Reporting bias, Indirectness, Imprecision, Heterogeneity |
| Daily rhGH:TransCon GH 0.14 mg/kg/wk | No concerns | Some concerns | Some concerns | Major concerns | Some concerns | No concerns | Low | Reporting bias, Indirectness, Imprecision, Heterogeneity |
| Daily rhGH:Somatrogon 0.66 mg/kg/wk | No concerns | Some concerns | No concerns | No concerns | Some concerns | No concerns | Moderate | Reporting bias, Heterogeneity |
| Daily rhGH:Lonapegsomatropin 0.24 mg/kg/wk | No concerns | Some concerns | No concerns | No concerns | Some concerns | No concerns | Moderate | Reporting bias, Heterogeneity |
| PEG-rhGH 0.1 mg/kg/wk:PEG-rhGH 0.2 mg/kg/wk | No concerns | Some concerns | No concerns | Major concerns | Some concerns | No concerns | Low | Reporting bias, Imprecision, Heterogeneity |
| PEG-rhGH 0.1 mg/kg/wk:TransCon GH 0.30 mg/kg/wk | No concerns | Some concerns | No concerns | Major concerns | Some concerns | No concerns | Low | Reporting bias, Imprecision, Heterogeneity |
| PEG-rhGH 0.1 mg/kg/wk:TransCon GH 0.14 mg/kg/wk | No concerns | Some concerns | No concerns | Major concerns | Some concerns | No concerns | Low | Reporting bias, Imprecision, Heterogeneity |
| PEG-rhGH 0.1 mg/kg/wk:Somatrogon 0.66 mg/kg/wk | No concerns | Some concerns | No concerns | Major concerns | Some concerns | No concerns | Low | Reporting bias, Imprecision, Heterogeneity |
| Lonapegsomatropin 0.24 mg/kg/wk:PEG-rhGH 0.1 mg/kg/wk | No concerns | Some concerns | No concerns | Major concerns | Some concerns | No concerns | Low | Reporting bias, Imprecision, Heterogeneity |
| PEG-rhGH 0.2 mg/kg/wk:TransCon GH 0.30 mg/kg/wk | No concerns | Some concerns | No concerns | Major concerns | Some concerns | No concerns | Low | Reporting bias, Imprecision, Heterogeneity |
| PEG-rhGH 0.2 mg/kg/wk:TransCon GH 0.14 mg/kg/wk | No concerns | Some concerns | No concerns | Major concerns | Some concerns | No concerns | Low | Reporting bias, Imprecision, Heterogeneity |
| PEG-rhGH 0.2 mg/kg/wk:Somatrogon 0.66 mg/kg/wk | No concerns | Some concerns | No concerns | Major concerns | Some concerns | No concerns | Low | Reporting bias, Imprecision, Heterogeneity |
| Lonapegsomatropin 0.24 mg/kg/wk:PEG-rhGH 0.2 mg/kg/wk | No concerns | Some concerns | No concerns | Major concerns | Some concerns | No concerns | Low | Reporting bias, Imprecision, Heterogeneity |
| TransCon GH 0.14 mg/kg/wk:TransCon GH 0.30 mg/kg/wk | No concerns | Some concerns | Some concerns | Major concerns | Some concerns | No concerns | Low | Reporting bias, Indirectness, Imprecision, Heterogeneity |
| Somatrogon 0.66 mg/kg/wk:TransCon GH 0.30 mg/kg/wk | No concerns | Some concerns | No concerns | Major concerns | Some concerns | No concerns | Low | Reporting bias, Imprecision, Heterogeneity |
| Lonapegsomatropin 0.24 mg/kg/wk:TransCon GH 0.30 mg/kg/wk | No concerns | Some concerns | No concerns | Major concerns | Some concerns | No concerns | Low | Reporting bias, Imprecision, Heterogeneity |
| Somatrogon 0.66 mg/kg/wk:TransCon GH 0.14 mg/kg/wk | No concerns | Some concerns | No concerns | Major concerns | Some concerns | No concerns | Low | Reporting bias, Imprecision, Heterogeneity |
| Lonapegsomatropin 0.24 mg/kg/wk:TransCon GH 0.14 mg/kg/wk | No concerns | Some concerns | No concerns | Major concerns | Some concerns | No concerns | Low | Reporting bias, Imprecision, Heterogeneity |
| Lonapegsomatropin 0.24 mg/kg/wk:Somatrogon 0.66 mg/kg/wk | No concerns | Some concerns | No concerns | Some concerns | Some concerns | No concerns | Low | Reporting bias, Imprecision, Heterogeneity |

S8.7 Certainty of evidence for Fever

| Comparison | Within_Study_Bias | Reporting_Bias | Indirectness | Imprecision | Heterogeneity | Incoherence | Overall_Confidence | Reason_for_downgrade |
| --- | --- | --- | --- | --- | --- | --- | --- | --- |
| Daily rhGH:TransCon GH 0.30 mg/kg/wk | No concerns | Some concerns | Some concerns | Some concerns | Some concerns | No concerns | Low | Reporting bias, Indirectness, Imprecision, Heterogeneity |
| Daily rhGH:Somatrogon 0.66 mg/kg/wk | No concerns | Some concerns | No concerns | No concerns | Some concerns | No concerns | Moderate | Reporting bias, Heterogeneity |
| Daily rhGH:Somatrogon 0.25/0.48/0.66 mg/kg/wk | No concerns | Some concerns | No concerns | Major concerns | Some concerns | No concerns | Low | Reporting bias, Imprecision, Heterogeneity |
| Daily rhGH:YPEG-rhGH 0.14 mg/kg/wk | No concerns | Some concerns | No concerns | No concerns | Some concerns | No concerns | Moderate | Reporting bias, Heterogeneity |
| Daily rhGH:Lonapegsomatropin 0.24 mg/kg/wk | No concerns | Some concerns | No concerns | Major concerns | Some concerns | No concerns | Low | Reporting bias, Imprecision, Heterogeneity |
| Somatrogon 0.66 mg/kg/wk:TransCon GH 0.30 mg/kg/wk | No concerns | Some concerns | No concerns | Some concerns | Some concerns | No concerns | Low | Reporting bias, Imprecision, Heterogeneity |
| Somatrogon 0.25/0.48/0.66 mg/kg/wk:TransCon GH 0.30 mg/kg/wk | No concerns | Some concerns | No concerns | Major concerns | Some concerns | No concerns | Low | Reporting bias, Imprecision, Heterogeneity |
| TransCon GH 0.30 mg/kg/wk:YPEG-rhGH 0.14 mg/kg/wk | No concerns | Some concerns | No concerns | Major concerns | Some concerns | No concerns | Low | Reporting bias, Imprecision, Heterogeneity |
| Lonapegsomatropin 0.24 mg/kg/wk:TransCon GH 0.30 mg/kg/wk | No concerns | Some concerns | No concerns | Some concerns | Some concerns | No concerns | Low | Reporting bias, Imprecision, Heterogeneity |
| Somatrogon 0.25/0.48/0.66 mg/kg/wk:Somatrogon 0.66 mg/kg/wk | No concerns | Some concerns | No concerns | Major concerns | Some concerns | No concerns | Low | Reporting bias, Imprecision, Heterogeneity |
| Somatrogon 0.66 mg/kg/wk:YPEG-rhGH 0.14 mg/kg/wk | No concerns | Some concerns | No concerns | No concerns | Some concerns | No concerns | Moderate | Reporting bias, Heterogeneity |
| Lonapegsomatropin 0.24 mg/kg/wk:Somatrogon 0.66 mg/kg/wk | No concerns | Some concerns | No concerns | Some concerns | Some concerns | No concerns | Low | Reporting bias, Imprecision, Heterogeneity |
| Somatrogon 0.25/0.48/0.66 mg/kg/wk:YPEG-rhGH 0.14 mg/kg/wk | No concerns | Some concerns | No concerns | Some concerns | Some concerns | No concerns | Low | Reporting bias, Imprecision, Heterogeneity |
| Lonapegsomatropin 0.24 mg/kg/wk:Somatrogon 0.25/0.48/0.66 mg/kg/wk | No concerns | Some concerns | No concerns | Major concerns | Some concerns | No concerns | Low | Reporting bias, Imprecision, Heterogeneity |
| Lonapegsomatropin 0.24 mg/kg/wk:YPEG-rhGH 0.14 mg/kg/wk | No concerns | Some concerns | No concerns | Some concerns | Some concerns | No concerns | Low | Reporting bias, Imprecision, Heterogeneity |

S8.8 Certainty of evidence for hypothyroidism

| Comparison | Within_Study_Bias | Reporting_Bias | Indirectness | Imprecision | Heterogeneity | Incoherence | Overall_Confidence | Reason_for_downgrade |
| --- | --- | --- | --- | --- | --- | --- | --- | --- |
| Daily rhGH:PEG-rhGH 0.1 mg/kg/wk | No concerns | Some concerns | No concerns | Major concerns | No concerns | No concerns | Low | Reporting bias, Imprecision |
| Daily rhGH:PEG-rhGH 0.2 mg/kg/wk | No concerns | Some concerns | No concerns | Major concerns | No concerns | No concerns | Low | Reporting bias, Imprecision |
| Daily rhGH:Somatrogon 0.66 mg/kg/wk | No concerns | Some concerns | No concerns | Major concerns | No concerns | No concerns | Low | Reporting bias, Imprecision |
| Daily rhGH:Somatrogon 0.25 mg/kg/wk | No concerns | Some concerns | No concerns | Major concerns | No concerns | No concerns | Low | Reporting bias, Imprecision |
| Daily rhGH:Somatrogon 0.48 mg/kg/wk | No concerns | Some concerns | No concerns | Major concerns | No concerns | No concerns | Low | Reporting bias, Imprecision |
| Daily rhGH:YPEG-rhGH 0.12 mg/kg/wk | No concerns | Some concerns | Some concerns | Some concerns | No concerns | No concerns | Low | Reporting bias, Indirectness, Imprecision |
| Daily rhGH:YPEG-rhGH 0.14 mg/kg/wk | No concerns | Some concerns | Some concerns | Some concerns | No concerns | No concerns | Low | Reporting bias, Indirectness, Imprecision |
| PEG-rhGH 0.1 mg/kg/wk:PEG-rhGH 0.2 mg/kg/wk | No concerns | Some concerns | No concerns | Major concerns | No concerns | No concerns | Low | Reporting bias, Imprecision |
| PEG-rhGH 0.1 mg/kg/wk:Somatrogon 0.66 mg/kg/wk | No concerns | Some concerns | No concerns | Major concerns | No concerns | No concerns | Low | Reporting bias, Imprecision |
| PEG-rhGH 0.1 mg/kg/wk:Somatrogon 0.25 mg/kg/wk | No concerns | Some concerns | No concerns | Major concerns | No concerns | No concerns | Low | Reporting bias, Imprecision |
| PEG-rhGH 0.1 mg/kg/wk:Somatrogon 0.48 mg/kg/wk | No concerns | Some concerns | No concerns | Major concerns | No concerns | No concerns | Low | Reporting bias, Imprecision |
| PEG-rhGH 0.1 mg/kg/wk:YPEG-rhGH 0.12 mg/kg/wk | No concerns | Some concerns | No concerns | Major concerns | No concerns | No concerns | Low | Reporting bias, Imprecision |
| PEG-rhGH 0.1 mg/kg/wk:YPEG-rhGH 0.14 mg/kg/wk | No concerns | Some concerns | No concerns | Major concerns | No concerns | No concerns | Low | Reporting bias, Imprecision |
| PEG-rhGH 0.2 mg/kg/wk:Somatrogon 0.66 mg/kg/wk | No concerns | Some concerns | No concerns | Major concerns | No concerns | No concerns | Low | Reporting bias, Imprecision |
| PEG-rhGH 0.2 mg/kg/wk:Somatrogon 0.25 mg/kg/wk | No concerns | Some concerns | No concerns | Major concerns | No concerns | No concerns | Low | Reporting bias, Imprecision |
| PEG-rhGH 0.2 mg/kg/wk:Somatrogon 0.48 mg/kg/wk | No concerns | Some concerns | No concerns | Major concerns | No concerns | No concerns | Low | Reporting bias, Imprecision |
| PEG-rhGH 0.2 mg/kg/wk:YPEG-rhGH 0.12 mg/kg/wk | No concerns | Some concerns | No concerns | Major concerns | No concerns | No concerns | Low | Reporting bias, Imprecision |
| PEG-rhGH 0.2 mg/kg/wk:YPEG-rhGH 0.14 mg/kg/wk | No concerns | Some concerns | No concerns | Major concerns | No concerns | No concerns | Low | Reporting bias, Imprecision |
| Somatrogon 0.25 mg/kg/wk:Somatrogon 0.66 mg/kg/wk | No concerns | Some concerns | No concerns | Major concerns | No concerns | No concerns | Low | Reporting bias, Imprecision |
| Somatrogon 0.48 mg/kg/wk:Somatrogon 0.66 mg/kg/wk | No concerns | Some concerns | No concerns | Major concerns | No concerns | No concerns | Low | Reporting bias, Imprecision |
| Somatrogon 0.66 mg/kg/wk:YPEG-rhGH 0.12 mg/kg/wk | No concerns | Some concerns | No concerns | Some concerns | No concerns | No concerns | Moderate | Reporting bias, Imprecision |
| Somatrogon 0.66 mg/kg/wk:YPEG-rhGH 0.14 mg/kg/wk | No concerns | Some concerns | No concerns | Some concerns | No concerns | No concerns | Moderate | Reporting bias, Imprecision |
| Somatrogon 0.25 mg/kg/wk:Somatrogon 0.48 mg/kg/wk | No concerns | Some concerns | No concerns | Major concerns | No concerns | No concerns | Low | Reporting bias, Imprecision |
| Somatrogon 0.25 mg/kg/wk:YPEG-rhGH 0.12 mg/kg/wk | No concerns | Some concerns | No concerns | Some concerns | No concerns | No concerns | Moderate | Reporting bias, Imprecision |
| Somatrogon 0.25 mg/kg/wk:YPEG-rhGH 0.14 mg/kg/wk | No concerns | Some concerns | No concerns | Some concerns | No concerns | No concerns | Moderate | Reporting bias, Imprecision |
| Somatrogon 0.48 mg/kg/wk:YPEG-rhGH 0.12 mg/kg/wk | No concerns | Some concerns | No concerns | Some concerns | No concerns | No concerns | Moderate | Reporting bias, Imprecision |
| Somatrogon 0.48 mg/kg/wk:YPEG-rhGH 0.14 mg/kg/wk | No concerns | Some concerns | No concerns | Some concerns | No concerns | No concerns | Moderate | Reporting bias, Imprecision |
| YPEG-rhGH 0.12 mg/kg/wk:YPEG-rhGH 0.14 mg/kg/wk | No concerns | Some concerns | Some concerns | Major concerns | No concerns | No concerns | Low | Reporting bias, Indirectness, Imprecision |

S8.9 Certainty of evidence for Injection site pain

| Comparison | Within_Study_Bias | Reporting_Bias | Indirectness | Imprecision | Heterogeneity | Incoherence | Overall_Confidence | Reason_for_downgrade |
| --- | --- | --- | --- | --- | --- | --- | --- | --- |
| Daily rhGH:TransCon GH 0.14 mg/kg/wk | No concerns | Some concerns | Some concerns | Some concerns | Some concerns | No concerns | Low | Reporting bias, Indirectness, Imprecision, Heterogeneity |
| Daily rhGH:TransCon GH 0.21 mg/kg/wk | No concerns | Some concerns | Some concerns | Some concerns | Some concerns | No concerns | Low | Reporting bias, Indirectness, Imprecision, Heterogeneity |
| Daily rhGH:TransCon GH 0.30 mg/kg/wk | No concerns | Some concerns | Some concerns | Some concerns | Some concerns | No concerns | Low | Reporting bias, Indirectness, Imprecision, Heterogeneity |
| Daily rhGH:Somatrogon 0.66 mg/kg/wk | No concerns | Some concerns | No concerns | Major concerns | Some concerns | No concerns | Low | Reporting bias, Imprecision, Heterogeneity |
| Daily rhGH:Somapacitan 0.16 mg/kg/wk | Some concerns | Some concerns | No concerns | Major concerns | Some concerns | No concerns | Low | Within-study bias, Reporting bias, Imprecision, Heterogeneity |
| Daily rhGH:Somatrogon 0.25 mg/kg/wk | No concerns | Some concerns | No concerns | Major concerns | Some concerns | No concerns | Low | Reporting bias, Imprecision, Heterogeneity |
| Daily rhGH:Somatrogon 0.48 mg/kg/wk | No concerns | Some concerns | No concerns | Major concerns | Some concerns | No concerns | Low | Reporting bias, Imprecision, Heterogeneity |
| Daily rhGH:YPEG-rhGH 0.1 mg/kg/wk | No concerns | Some concerns | Some concerns | Major concerns | Some concerns | No concerns | Low | Reporting bias, Indirectness, Imprecision, Heterogeneity |
| Daily rhGH:YPEG-rhGH 0.12 mg/kg/wk | No concerns | Some concerns | Some concerns | Major concerns | Some concerns | No concerns | Low | Reporting bias, Indirectness, Imprecision, Heterogeneity |
| Daily rhGH:YPEG-rhGH 0.14 mg/kg/wk | No concerns | Some concerns | Some concerns | Major concerns | Some concerns | No concerns | Low | Reporting bias, Indirectness, Imprecision, Heterogeneity |
| TransCon GH 0.14 mg/kg/wk:TransCon GH 0.21 mg/kg/wk | No concerns | Some concerns | Some concerns | Major concerns | Some concerns | No concerns | Low | Reporting bias, Indirectness, Imprecision, Heterogeneity |
| TransCon GH 0.14 mg/kg/wk:TransCon GH 0.30 mg/kg/wk | No concerns | Some concerns | Some concerns | Major concerns | Some concerns | No concerns | Low | Reporting bias, Indirectness, Imprecision, Heterogeneity |
| Somatrogon 0.66 mg/kg/wk:TransCon GH 0.14 mg/kg/wk | No concerns | Some concerns | No concerns | Some concerns | Some concerns | No concerns | Low | Reporting bias, Imprecision, Heterogeneity |
| Somapacitan 0.16 mg/kg/wk:TransCon GH 0.14 mg/kg/wk | Some concerns | Some concerns | Some concerns | Major concerns | Some concerns | No concerns | Low | Within-study bias, Reporting bias, Indirectness, Imprecision, Heterogeneity |
| Somatrogon 0.25 mg/kg/wk:TransCon GH 0.14 mg/kg/wk | No concerns | Some concerns | No concerns | Some concerns | Some concerns | No concerns | Low | Reporting bias, Imprecision, Heterogeneity |
| Somatrogon 0.48 mg/kg/wk:TransCon GH 0.14 mg/kg/wk | No concerns | Some concerns | No concerns | Some concerns | Some concerns | No concerns | Low | Reporting bias, Imprecision, Heterogeneity |
| TransCon GH 0.14 mg/kg/wk:YPEG-rhGH 0.1 mg/kg/wk | No concerns | Some concerns | Some concerns | Major concerns | Some concerns | No concerns | Low | Reporting bias, Indirectness, Imprecision, Heterogeneity |
| TransCon GH 0.14 mg/kg/wk:YPEG-rhGH 0.12 mg/kg/wk | No concerns | Some concerns | Some concerns | Major concerns | Some concerns | No concerns | Low | Reporting bias, Indirectness, Imprecision, Heterogeneity |
| TransCon GH 0.14 mg/kg/wk:YPEG-rhGH 0.14 mg/kg/wk | No concerns | Some concerns | Some concerns | Major concerns | Some concerns | No concerns | Low | Reporting bias, Indirectness, Imprecision, Heterogeneity |
| TransCon GH 0.21 mg/kg/wk:TransCon GH 0.30 mg/kg/wk | No concerns | Some concerns | Some concerns | Major concerns | Some concerns | No concerns | Low | Reporting bias, Indirectness, Imprecision, Heterogeneity |
| Somatrogon 0.66 mg/kg/wk:TransCon GH 0.21 mg/kg/wk | No concerns | Some concerns | No concerns | Some concerns | Some concerns | No concerns | Low | Reporting bias, Imprecision, Heterogeneity |
| Somapacitan 0.16 mg/kg/wk:TransCon GH 0.21 mg/kg/wk | Some concerns | Some concerns | Some concerns | Major concerns | Some concerns | No concerns | Low | Within-study bias, Reporting bias, Indirectness, Imprecision, Heterogeneity |
| Somatrogon 0.25 mg/kg/wk:TransCon GH 0.21 mg/kg/wk | No concerns | Some concerns | No concerns | Some concerns | Some concerns | No concerns | Low | Reporting bias, Imprecision, Heterogeneity |
| Somatrogon 0.48 mg/kg/wk:TransCon GH 0.21 mg/kg/wk | No concerns | Some concerns | No concerns | Some concerns | Some concerns | No concerns | Low | Reporting bias, Imprecision, Heterogeneity |
| TransCon GH 0.21 mg/kg/wk:YPEG-rhGH 0.1 mg/kg/wk | No concerns | Some concerns | Some concerns | Major concerns | Some concerns | No concerns | Low | Reporting bias, Indirectness, Imprecision, Heterogeneity |
| TransCon GH 0.21 mg/kg/wk:YPEG-rhGH 0.12 mg/kg/wk | No concerns | Some concerns | Some concerns | Major concerns | Some concerns | No concerns | Low | Reporting bias, Indirectness, Imprecision, Heterogeneity |
| TransCon GH 0.21 mg/kg/wk:YPEG-rhGH 0.14 mg/kg/wk | No concerns | Some concerns | Some concerns | Major concerns | Some concerns | No concerns | Low | Reporting bias, Indirectness, Imprecision, Heterogeneity |
| Somatrogon 0.66 mg/kg/wk:TransCon GH 0.30 mg/kg/wk | No concerns | Some concerns | No concerns | Some concerns | Some concerns | No concerns | Low | Reporting bias, Imprecision, Heterogeneity |
| Somapacitan 0.16 mg/kg/wk:TransCon GH 0.30 mg/kg/wk | Some concerns | Some concerns | Some concerns | Major concerns | Some concerns | No concerns | Low | Within-study bias, Reporting bias, Indirectness, Imprecision, Heterogeneity |
| Somatrogon 0.25 mg/kg/wk:TransCon GH 0.30 mg/kg/wk | No concerns | Some concerns | No concerns | Some concerns | Some concerns | No concerns | Low | Reporting bias, Imprecision, Heterogeneity |
| Somatrogon 0.48 mg/kg/wk:TransCon GH 0.30 mg/kg/wk | No concerns | Some concerns | No concerns | Some concerns | Some concerns | No concerns | Low | Reporting bias, Imprecision, Heterogeneity |
| TransCon GH 0.30 mg/kg/wk:YPEG-rhGH 0.1 mg/kg/wk | No concerns | Some concerns | Some concerns | Major concerns | Some concerns | No concerns | Low | Reporting bias, Indirectness, Imprecision, Heterogeneity |
| TransCon GH 0.30 mg/kg/wk:YPEG-rhGH 0.12 mg/kg/wk | No concerns | Some concerns | Some concerns | Major concerns | Some concerns | No concerns | Low | Reporting bias, Indirectness, Imprecision, Heterogeneity |
| TransCon GH 0.30 mg/kg/wk:YPEG-rhGH 0.14 mg/kg/wk | No concerns | Some concerns | Some concerns | Major concerns | Some concerns | No concerns | Low | Reporting bias, Indirectness, Imprecision, Heterogeneity |
| Somapacitan 0.16 mg/kg/wk:Somatrogon 0.66 mg/kg/wk | Some concerns | Some concerns | No concerns | Major concerns | Some concerns | No concerns | Low | Within-study bias, Reporting bias, Imprecision, Heterogeneity |
| Somatrogon 0.25 mg/kg/wk:Somatrogon 0.66 mg/kg/wk | No concerns | Some concerns | No concerns | Some concerns | Some concerns | No concerns | Low | Reporting bias, Imprecision, Heterogeneity |
| Somatrogon 0.48 mg/kg/wk:Somatrogon 0.66 mg/kg/wk | No concerns | Some concerns | No concerns | Some concerns | Some concerns | No concerns | Low | Reporting bias, Imprecision, Heterogeneity |
| Somatrogon 0.66 mg/kg/wk:YPEG-rhGH 0.1 mg/kg/wk | No concerns | Some concerns | No concerns | Major concerns | Some concerns | No concerns | Low | Reporting bias, Imprecision, Heterogeneity |
| Somatrogon 0.66 mg/kg/wk:YPEG-rhGH 0.12 mg/kg/wk | No concerns | Some concerns | No concerns | Major concerns | Some concerns | No concerns | Low | Reporting bias, Imprecision, Heterogeneity |
| Somatrogon 0.66 mg/kg/wk:YPEG-rhGH 0.14 mg/kg/wk | No concerns | Some concerns | No concerns | Major concerns | Some concerns | No concerns | Low | Reporting bias, Imprecision, Heterogeneity |
| Somapacitan 0.16 mg/kg/wk:Somatrogon 0.25 mg/kg/wk | Some concerns | Some concerns | No concerns | Major concerns | Some concerns | No concerns | Low | Within-study bias, Reporting bias, Imprecision, Heterogeneity |
| Somapacitan 0.16 mg/kg/wk:Somatrogon 0.48 mg/kg/wk | Some concerns | Some concerns | No concerns | Major concerns | Some concerns | No concerns | Low | Within-study bias, Reporting bias, Imprecision, Heterogeneity |
| Somapacitan 0.16 mg/kg/wk:YPEG-rhGH 0.1 mg/kg/wk | Some concerns | Some concerns | Some concerns | Major concerns | Some concerns | No concerns | Low | Within-study bias, Reporting bias, Indirectness, Imprecision, Heterogeneity |
| Somapacitan 0.16 mg/kg/wk:YPEG-rhGH 0.12 mg/kg/wk | Some concerns | Some concerns | Some concerns | Major concerns | Some concerns | No concerns | Low | Within-study bias, Reporting bias, Indirectness, Imprecision, Heterogeneity |
| Somapacitan 0.16 mg/kg/wk:YPEG-rhGH 0.14 mg/kg/wk | Some concerns | Some concerns | Some concerns | Major concerns | Some concerns | No concerns | Low | Within-study bias, Reporting bias, Indirectness, Imprecision, Heterogeneity |
| Somatrogon 0.25 mg/kg/wk:Somatrogon 0.48 mg/kg/wk | No concerns | Some concerns | No concerns | Major concerns | Some concerns | No concerns | Low | Reporting bias, Imprecision, Heterogeneity |
| Somatrogon 0.25 mg/kg/wk:YPEG-rhGH 0.1 mg/kg/wk | No concerns | Some concerns | No concerns | Major concerns | Some concerns | No concerns | Low | Reporting bias, Imprecision, Heterogeneity |
| Somatrogon 0.25 mg/kg/wk:YPEG-rhGH 0.12 mg/kg/wk | No concerns | Some concerns | No concerns | Major concerns | Some concerns | No concerns | Low | Reporting bias, Imprecision, Heterogeneity |
| Somatrogon 0.25 mg/kg/wk:YPEG-rhGH 0.14 mg/kg/wk | No concerns | Some concerns | No concerns | Major concerns | Some concerns | No concerns | Low | Reporting bias, Imprecision, Heterogeneity |
| Somatrogon 0.48 mg/kg/wk:YPEG-rhGH 0.1 mg/kg/wk | No concerns | Some concerns | No concerns | Major concerns | Some concerns | No concerns | Low | Reporting bias, Imprecision, Heterogeneity |
| Somatrogon 0.48 mg/kg/wk:YPEG-rhGH 0.12 mg/kg/wk | No concerns | Some concerns | No concerns | Major concerns | Some concerns | No concerns | Low | Reporting bias, Imprecision, Heterogeneity |
| Somatrogon 0.48 mg/kg/wk:YPEG-rhGH 0.14 mg/kg/wk | No concerns | Some concerns | No concerns | Major concerns | Some concerns | No concerns | Low | Reporting bias, Imprecision, Heterogeneity |
| YPEG-rhGH 0.1 mg/kg/wk:YPEG-rhGH 0.12 mg/kg/wk | No concerns | Some concerns | Some concerns | Major concerns | Some concerns | No concerns | Low | Reporting bias, Indirectness, Imprecision, Heterogeneity |
| YPEG-rhGH 0.1 mg/kg/wk:YPEG-rhGH 0.14 mg/kg/wk | No concerns | Some concerns | Some concerns | Major concerns | Some concerns | No concerns | Low | Reporting bias, Indirectness, Imprecision, Heterogeneity |
| YPEG-rhGH 0.12 mg/kg/wk:YPEG-rhGH 0.14 mg/kg/wk | No concerns | Some concerns | Some concerns | Major concerns | Some concerns | No concerns | Low | Reporting bias, Indirectness, Imprecision, Heterogeneity |

S8.10 Certainty of evidence for IGF-1

| Comparison | Within_Study_Bias | Reporting_Bias | Indirectness | Imprecision | Heterogeneity | Incoherence | Overall_Confidence | Reason_for_downgrade |
| --- | --- | --- | --- | --- | --- | --- | --- | --- |
| Daily rhGH:PEG-rhGH 0.1 mg/kg/wk | No concerns | Some concerns | No concerns | No concerns | No concerns | No concerns | Moderate | Reporting bias |
| Daily rhGH:PEG-rhGH 0.2 mg/kg/wk | No concerns | Some concerns | No concerns | No concerns | No concerns | No concerns | Moderate | Reporting bias |
| Daily rhGH:Somapacitan 0.16 mg/kg/wk | Some concerns | Some concerns | No concerns | No concerns | No concerns | No concerns | Moderate | Within-study bias, Reporting bias |
| Daily rhGH:Somatrogon 0.25/0.48/0.66 mg/kg/wk | No concerns | Some concerns | No concerns | No concerns | No concerns | No concerns | Moderate | Reporting bias |
| Daily rhGH:YPEG-rhGH 0.14 mg/kg/wk | No concerns | Some concerns | No concerns | No concerns | No concerns | No concerns | Moderate | Reporting bias |
| Daily rhGH:Somapacitan 0.08 mg/kg/wk | Major concerns | Some concerns | Some concerns | Major concerns | No concerns | No concerns | Very low | Within-study bias, Reporting bias, Indirectness, Imprecision |
| Daily rhGH:Somapacitan 0.04mg/kg/wk | Major concerns | Some concerns | Some concerns | Some concerns | No concerns | No concerns | Low | Within-study bias, Reporting bias, Indirectness, Imprecision |
| Daily rhGH:Somapacitan 0.04/0.16 mg/kg/wk | Major concerns | Some concerns | No concerns | Major concerns | No concerns | No concerns | Very low | Within-study bias, Reporting bias, Imprecision |
| Daily rhGH:Somapacitan 0.08/0.16 mg/kg/wk | Major concerns | Some concerns | No concerns | Major concerns | No concerns | No concerns | Very low | Within-study bias, Reporting bias, Imprecision |
| Daily rhGH:Somapacitan 0.16/0.16 mg/kg/wk | Major concerns | Some concerns | No concerns | No concerns | No concerns | No concerns | Low | Within-study bias, Reporting bias |
| Daily rhGH:Lonapegsomatropin 0.24 mg/kg/wk | No concerns | Some concerns | No concerns | Major concerns | No concerns | No concerns | Low | Reporting bias, Imprecision |
| PEG-rhGH 0.1 mg/kg/wk:PEG-rhGH 0.2 mg/kg/wk | No concerns | Some concerns | No concerns | Major concerns | No concerns | No concerns | Low | Reporting bias, Imprecision |
| PEG-rhGH 0.1 mg/kg/wk:Somapacitan 0.16 mg/kg/wk | Some concerns | Some concerns | No concerns | Major concerns | No concerns | No concerns | Low | Within-study bias, Reporting bias, Imprecision |
| PEG-rhGH 0.1 mg/kg/wk:Somatrogon 0.25/0.48/0.66 mg/kg/wk | No concerns | Some concerns | No concerns | No concerns | No concerns | No concerns | Moderate | Reporting bias |
| PEG-rhGH 0.1 mg/kg/wk:YPEG-rhGH 0.14 mg/kg/wk | No concerns | Some concerns | No concerns | Major concerns | No concerns | No concerns | Low | Reporting bias, Imprecision |
| PEG-rhGH 0.1 mg/kg/wk:Somapacitan 0.08 mg/kg/wk | Some concerns | Some concerns | No concerns | Major concerns | No concerns | No concerns | Low | Within-study bias, Reporting bias, Imprecision |
| PEG-rhGH 0.1 mg/kg/wk:Somapacitan 0.04mg/kg/wk | Some concerns | Some concerns | No concerns | Some concerns | No concerns | No concerns | Low | Within-study bias, Reporting bias, Imprecision |
| PEG-rhGH 0.1 mg/kg/wk:Somapacitan 0.04/0.16 mg/kg/wk | Some concerns | Some concerns | No concerns | Major concerns | No concerns | No concerns | Low | Within-study bias, Reporting bias, Imprecision |
| PEG-rhGH 0.1 mg/kg/wk:Somapacitan 0.08/0.16 mg/kg/wk | Some concerns | Some concerns | No concerns | Major concerns | No concerns | No concerns | Low | Within-study bias, Reporting bias, Imprecision |
| PEG-rhGH 0.1 mg/kg/wk:Somapacitan 0.16/0.16 mg/kg/wk | Some concerns | Some concerns | No concerns | Major concerns | No concerns | No concerns | Low | Within-study bias, Reporting bias, Imprecision |
| Lonapegsomatropin 0.24 mg/kg/wk:PEG-rhGH 0.1 mg/kg/wk | No concerns | Some concerns | No concerns | No concerns | No concerns | No concerns | Moderate | Reporting bias |
| PEG-rhGH 0.2 mg/kg/wk:Somapacitan 0.16 mg/kg/wk | Some concerns | Some concerns | No concerns | No concerns | No concerns | No concerns | Moderate | Within-study bias, Reporting bias |
| PEG-rhGH 0.2 mg/kg/wk:Somatrogon 0.25/0.48/0.66 mg/kg/wk | No concerns | Some concerns | No concerns | No concerns | No concerns | No concerns | Moderate | Reporting bias |
| PEG-rhGH 0.2 mg/kg/wk:YPEG-rhGH 0.14 mg/kg/wk | No concerns | Some concerns | No concerns | No concerns | No concerns | No concerns | Moderate | Reporting bias |
| PEG-rhGH 0.2 mg/kg/wk:Somapacitan 0.08 mg/kg/wk | Some concerns | Some concerns | No concerns | Major concerns | No concerns | No concerns | Low | Within-study bias, Reporting bias, Imprecision |
| PEG-rhGH 0.2 mg/kg/wk:Somapacitan 0.04mg/kg/wk | Some concerns | Some concerns | No concerns | Some concerns | No concerns | No concerns | Low | Within-study bias, Reporting bias, Imprecision |
| PEG-rhGH 0.2 mg/kg/wk:Somapacitan 0.04/0.16 mg/kg/wk | Some concerns | Some concerns | No concerns | Major concerns | No concerns | No concerns | Low | Within-study bias, Reporting bias, Imprecision |
| PEG-rhGH 0.2 mg/kg/wk:Somapacitan 0.08/0.16 mg/kg/wk | Some concerns | Some concerns | No concerns | Major concerns | No concerns | No concerns | Low | Within-study bias, Reporting bias, Imprecision |
| PEG-rhGH 0.2 mg/kg/wk:Somapacitan 0.16/0.16 mg/kg/wk | Some concerns | Some concerns | No concerns | No concerns | No concerns | No concerns | Moderate | Within-study bias, Reporting bias |
| Lonapegsomatropin 0.24 mg/kg/wk:PEG-rhGH 0.2 mg/kg/wk | No concerns | Some concerns | No concerns | No concerns | No concerns | No concerns | Moderate | Reporting bias |
| Somapacitan 0.16 mg/kg/wk:Somatrogon 0.25/0.48/0.66 mg/kg/wk | Some concerns | Some concerns | No concerns | No concerns | No concerns | No concerns | Moderate | Within-study bias, Reporting bias |
| Somapacitan 0.16 mg/kg/wk:YPEG-rhGH 0.14 mg/kg/wk | Some concerns | Some concerns | No concerns | No concerns | No concerns | No concerns | Moderate | Within-study bias, Reporting bias |
| Somapacitan 0.08 mg/kg/wk:Somapacitan 0.16 mg/kg/wk | Some concerns | Some concerns | Some concerns | Major concerns | No concerns | No concerns | Low | Within-study bias, Reporting bias, Indirectness, Imprecision |
| Somapacitan 0.04mg/kg/wk:Somapacitan 0.16 mg/kg/wk | Some concerns | Some concerns | Some concerns | Major concerns | No concerns | No concerns | Low | Within-study bias, Reporting bias, Indirectness, Imprecision |
| Somapacitan 0.04/0.16 mg/kg/wk:Somapacitan 0.16 mg/kg/wk | Some concerns | Some concerns | No concerns | No concerns | No concerns | No concerns | Moderate | Within-study bias, Reporting bias |
| Somapacitan 0.08/0.16 mg/kg/wk:Somapacitan 0.16 mg/kg/wk | Some concerns | Some concerns | No concerns | Major concerns | No concerns | No concerns | Low | Within-study bias, Reporting bias, Imprecision |
| Somapacitan 0.16 mg/kg/wk:Somapacitan 0.16/0.16 mg/kg/wk | Some concerns | Some concerns | No concerns | No concerns | No concerns | No concerns | Moderate | Within-study bias, Reporting bias |
| Lonapegsomatropin 0.24 mg/kg/wk:Somapacitan 0.16 mg/kg/wk | Some concerns | Some concerns | No concerns | No concerns | No concerns | No concerns | Moderate | Within-study bias, Reporting bias |
| Somatrogon 0.25/0.48/0.66 mg/kg/wk:YPEG-rhGH 0.14 mg/kg/wk | No concerns | Some concerns | No concerns | No concerns | No concerns | No concerns | Moderate | Reporting bias |
| Somapacitan 0.08 mg/kg/wk:Somatrogon 0.25/0.48/0.66 mg/kg/wk | Some concerns | Some concerns | No concerns | Some concerns | No concerns | No concerns | Low | Within-study bias, Reporting bias, Imprecision |
| Somapacitan 0.04mg/kg/wk:Somatrogon 0.25/0.48/0.66 mg/kg/wk | Some concerns | Some concerns | No concerns | Some concerns | No concerns | No concerns | Low | Within-study bias, Reporting bias, Imprecision |
| Somapacitan 0.04/0.16 mg/kg/wk:Somatrogon 0.25/0.48/0.66 mg/kg/wk | Some concerns | Some concerns | No concerns | Some concerns | No concerns | No concerns | Low | Within-study bias, Reporting bias, Imprecision |
| Somapacitan 0.08/0.16 mg/kg/wk:Somatrogon 0.25/0.48/0.66 mg/kg/wk | Some concerns | Some concerns | No concerns | Some concerns | No concerns | No concerns | Low | Within-study bias, Reporting bias, Imprecision |
| Somapacitan 0.16/0.16 mg/kg/wk:Somatrogon 0.25/0.48/0.66 mg/kg/wk | Some concerns | Some concerns | No concerns | No concerns | No concerns | No concerns | Moderate | Within-study bias, Reporting bias |
| Lonapegsomatropin 0.24 mg/kg/wk:Somatrogon 0.25/0.48/0.66 mg/kg/wk | No concerns | Some concerns | No concerns | No concerns | No concerns | No concerns | Moderate | Reporting bias |
| Somapacitan 0.08 mg/kg/wk:YPEG-rhGH 0.14 mg/kg/wk | Some concerns | Some concerns | No concerns | Major concerns | No concerns | No concerns | Low | Within-study bias, Reporting bias, Imprecision |
| Somapacitan 0.04mg/kg/wk:YPEG-rhGH 0.14 mg/kg/wk | Some concerns | Some concerns | No concerns | Major concerns | No concerns | No concerns | Low | Within-study bias, Reporting bias, Imprecision |
| Somapacitan 0.04/0.16 mg/kg/wk:YPEG-rhGH 0.14 mg/kg/wk | Some concerns | Some concerns | No concerns | No concerns | No concerns | No concerns | Moderate | Within-study bias, Reporting bias |
| Somapacitan 0.08/0.16 mg/kg/wk:YPEG-rhGH 0.14 mg/kg/wk | Some concerns | Some concerns | No concerns | Major concerns | No concerns | No concerns | Low | Within-study bias, Reporting bias, Imprecision |
| Somapacitan 0.16/0.16 mg/kg/wk:YPEG-rhGH 0.14 mg/kg/wk | Some concerns | Some concerns | No concerns | Major concerns | No concerns | No concerns | Low | Within-study bias, Reporting bias, Imprecision |
| Lonapegsomatropin 0.24 mg/kg/wk:YPEG-rhGH 0.14 mg/kg/wk | No concerns | Some concerns | No concerns | No concerns | No concerns | No concerns | Moderate | Reporting bias |
| Somapacitan 0.04mg/kg/wk:Somapacitan 0.08 mg/kg/wk | Major concerns | Some concerns | Some concerns | Major concerns | No concerns | No concerns | Very low | Within-study bias, Reporting bias, Indirectness, Imprecision |
| Somapacitan 0.04/0.16 mg/kg/wk:Somapacitan 0.08 mg/kg/wk | Major concerns | Some concerns | No concerns | Major concerns | No concerns | No concerns | Very low | Within-study bias, Reporting bias, Imprecision |
| Somapacitan 0.08 mg/kg/wk:Somapacitan 0.08/0.16 mg/kg/wk | Major concerns | Some concerns | No concerns | Major concerns | No concerns | No concerns | Very low | Within-study bias, Reporting bias, Imprecision |
| Somapacitan 0.08 mg/kg/wk:Somapacitan 0.16/0.16 mg/kg/wk | Major concerns | Some concerns | No concerns | Some concerns | No concerns | No concerns | Low | Within-study bias, Reporting bias, Imprecision |
| Lonapegsomatropin 0.24 mg/kg/wk:Somapacitan 0.08 mg/kg/wk | Some concerns | Some concerns | No concerns | Some concerns | No concerns | No concerns | Low | Within-study bias, Reporting bias, Imprecision |
| Somapacitan 0.04/0.16 mg/kg/wk:Somapacitan 0.04mg/kg/wk | Major concerns | Some concerns | No concerns | Some concerns | No concerns | No concerns | Low | Within-study bias, Reporting bias, Imprecision |
| Somapacitan 0.04mg/kg/wk:Somapacitan 0.08/0.16 mg/kg/wk | Major concerns | Some concerns | No concerns | Major concerns | No concerns | No concerns | Very low | Within-study bias, Reporting bias, Imprecision |
| Somapacitan 0.04mg/kg/wk:Somapacitan 0.16/0.16 mg/kg/wk | Major concerns | Some concerns | No concerns | Major concerns | No concerns | No concerns | Very low | Within-study bias, Reporting bias, Imprecision |
| Lonapegsomatropin 0.24 mg/kg/wk:Somapacitan 0.04mg/kg/wk | Some concerns | Some concerns | No concerns | Some concerns | No concerns | No concerns | Low | Within-study bias, Reporting bias, Imprecision |
| Somapacitan 0.04/0.16 mg/kg/wk:Somapacitan 0.08/0.16 mg/kg/wk | Major concerns | Some concerns | No concerns | Major concerns | No concerns | No concerns | Very low | Within-study bias, Reporting bias, Imprecision |
| Somapacitan 0.04/0.16 mg/kg/wk:Somapacitan 0.16/0.16 mg/kg/wk | Major concerns | Some concerns | No concerns | Some concerns | No concerns | No concerns | Low | Within-study bias, Reporting bias, Imprecision |
| Lonapegsomatropin 0.24 mg/kg/wk:Somapacitan 0.04/0.16 mg/kg/wk | Some concerns | Some concerns | No concerns | No concerns | No concerns | No concerns | Moderate | Within-study bias, Reporting bias |
| Somapacitan 0.08/0.16 mg/kg/wk:Somapacitan 0.16/0.16 mg/kg/wk | Major concerns | Some concerns | No concerns | Some concerns | No concerns | No concerns | Low | Within-study bias, Reporting bias, Imprecision |
| Lonapegsomatropin 0.24 mg/kg/wk:Somapacitan 0.08/0.16 mg/kg/wk | Some concerns | Some concerns | No concerns | Some concerns | No concerns | No concerns | Low | Within-study bias, Reporting bias, Imprecision |
| Lonapegsomatropin 0.24 mg/kg/wk:Somapacitan 0.16/0.16 mg/kg/wk | Some concerns | Some concerns | No concerns | No concerns | No concerns | No concerns | Moderate | Within-study bias, Reporting bias |

S8.11 Certainty of evidence for HbA1C%

| Comparison | Within_Study_Bias | Reporting_Bias | Indirectness | Imprecision | Heterogeneity | Incoherence | Overall_Confidence | Reason_for_downgrade |
| --- | --- | --- | --- | --- | --- | --- | --- | --- |
| Daily rhGH:PEG-rhGH 0.2 mg/kg/wk | No concerns | Some concerns | No concerns | No concerns | Some concerns | No concerns | Moderate | Reporting bias, Heterogeneity |
| Daily rhGH:TransCon GH 0.14 mg/kg/wk | No concerns | Some concerns | Some concerns | No concerns | Some concerns | No concerns | Low | Reporting bias, Indirectness, Heterogeneity |
| Daily rhGH:TransCon GH 0.21 mg/kg/wk | No concerns | Some concerns | Some concerns | No concerns | Some concerns | No concerns | Low | Reporting bias, Indirectness, Heterogeneity |
| Daily rhGH:TransCon GH 0.30 mg/kg/wk | No concerns | Some concerns | Some concerns | No concerns | Some concerns | No concerns | Low | Reporting bias, Indirectness, Heterogeneity |
| Daily rhGH:Lonapegsomatropin 0.24 mg/kg/wk | No concerns | Some concerns | No concerns | No concerns | Some concerns | No concerns | Moderate | Reporting bias, Heterogeneity |
| PEG-rhGH 0.2 mg/kg/wk:TransCon GH 0.14 mg/kg/wk | No concerns | Some concerns | No concerns | No concerns | Some concerns | No concerns | Moderate | Reporting bias, Heterogeneity |
| PEG-rhGH 0.2 mg/kg/wk:TransCon GH 0.21 mg/kg/wk | No concerns | Some concerns | No concerns | No concerns | Some concerns | No concerns | Moderate | Reporting bias, Heterogeneity |
| PEG-rhGH 0.2 mg/kg/wk:TransCon GH 0.30 mg/kg/wk | No concerns | Some concerns | No concerns | No concerns | Some concerns | No concerns | Moderate | Reporting bias, Heterogeneity |
| Lonapegsomatropin 0.24 mg/kg/wk:PEG-rhGH 0.2 mg/kg/wk | No concerns | Some concerns | No concerns | No concerns | Some concerns | No concerns | Moderate | Reporting bias, Heterogeneity |
| TransCon GH 0.14 mg/kg/wk:TransCon GH 0.21 mg/kg/wk | No concerns | Some concerns | Some concerns | No concerns | Some concerns | No concerns | Low | Reporting bias, Indirectness, Heterogeneity |
| TransCon GH 0.14 mg/kg/wk:TransCon GH 0.30 mg/kg/wk | No concerns | Some concerns | Some concerns | No concerns | Some concerns | No concerns | Low | Reporting bias, Indirectness, Heterogeneity |
| Lonapegsomatropin 0.24 mg/kg/wk:TransCon GH 0.14 mg/kg/wk | No concerns | Some concerns | No concerns | No concerns | Some concerns | No concerns | Moderate | Reporting bias, Heterogeneity |
| TransCon GH 0.21 mg/kg/wk:TransCon GH 0.30 mg/kg/wk | No concerns | Some concerns | Some concerns | No concerns | Some concerns | No concerns | Low | Reporting bias, Indirectness, Heterogeneity |
| Lonapegsomatropin 0.24 mg/kg/wk:TransCon GH 0.21 mg/kg/wk | No concerns | Some concerns | No concerns | No concerns | Some concerns | No concerns | Moderate | Reporting bias, Heterogeneity |
| Lonapegsomatropin 0.24 mg/kg/wk:TransCon GH 0.30 mg/kg/wk | No concerns | Some concerns | No concerns | No concerns | Some concerns | No concerns | Moderate | Reporting bias, Heterogeneity |

S8.12 Certainty of evidence for cough

| Comparison | Within_Study_Bias | Reporting_Bias | Indirectness | Imprecision | Heterogeneity | Incoherence | Overall_Confidence | Reason_for_downgrade |
| --- | --- | --- | --- | --- | --- | --- | --- | --- |
| Daily rhGH:Somatrogon 0.66 mg/kg/wk | No concerns | Some concerns | No concerns | No concerns | Some concerns | No concerns | Moderate | Reporting bias, Heterogeneity |
| Daily rhGH:YPEG-rhGH 0.14 mg/kg/wk | No concerns | Some concerns | No concerns | No concerns | Some concerns | No concerns | Moderate | Reporting bias, Heterogeneity |
| Daily rhGH:Somapacitan 0.04mg/kg/wk | Major concerns | Some concerns | Some concerns | Major concerns | Some concerns | No concerns | Very low | Within-study bias, Reporting bias, Indirectness, Imprecision, Heterogeneity |
| Somatrogon 0.66 mg/kg/wk:YPEG-rhGH 0.14 mg/kg/wk | No concerns | Some concerns | No concerns | No concerns | Some concerns | No concerns | Moderate | Reporting bias, Heterogeneity |
| Somapacitan 0.04mg/kg/wk:Somatrogon 0.66 mg/kg/wk | Some concerns | Some concerns | No concerns | Major concerns | Some concerns | No concerns | Low | Within-study bias, Reporting bias, Imprecision, Heterogeneity |
| Somapacitan 0.04mg/kg/wk:YPEG-rhGH 0.14 mg/kg/wk | Some concerns | Some concerns | No concerns | Major concerns | Some concerns | No concerns | Low | Within-study bias, Reporting bias, Imprecision, Heterogeneity |

S8.13 Certainty of evidence for URTI

| Comparison | Within_Study_Bias | Reporting_Bias | Indirectness | Imprecision | Heterogeneity | Incoherence | Overall_Confidence | Reason_for_downgrade |
| --- | --- | --- | --- | --- | --- | --- | --- | --- |
| Daily rhGH:YPEG-rhGH 0.14 mg/kg/wk | No concerns | Some concerns | No concerns | No concerns | No concerns | No concerns | Moderate | Reporting bias |
| Daily rhGH:Somapacitan 0.04 mg/kg/wk | Major concerns | Some concerns | Some concerns | Major concerns | No concerns | No concerns | Very low | Within-study bias, Reporting bias, Indirectness, Imprecision |
| Daily rhGH:Somapacitan 0.08 mg/kg/wk | Major concerns | Some concerns | Some concerns | Major concerns | No concerns | No concerns | Very low | Within-study bias, Reporting bias, Indirectness, Imprecision |
| Daily rhGH:Somapacitan 0.16 mg/kg/wk | Major concerns | Some concerns | No concerns | Some concerns | No concerns | No concerns | Low | Within-study bias, Reporting bias, Imprecision |
| Daily rhGH:Lonapegsomatropin 0.24 mg/kg/wk | No concerns | Some concerns | No concerns | No concerns | No concerns | No concerns | Moderate | Reporting bias |
| Somapacitan 0.04 mg/kg/wk:YPEG-rhGH 0.14 mg/kg/wk | Some concerns | Some concerns | No concerns | Major concerns | No concerns | No concerns | Low | Within-study bias, Reporting bias, Imprecision |
| Somapacitan 0.08 mg/kg/wk:YPEG-rhGH 0.14 mg/kg/wk | Some concerns | Some concerns | No concerns | Major concerns | No concerns | No concerns | Low | Within-study bias, Reporting bias, Imprecision |
| Somapacitan 0.16 mg/kg/wk:YPEG-rhGH 0.14 mg/kg/wk | Some concerns | Some concerns | No concerns | Major concerns | No concerns | No concerns | Low | Within-study bias, Reporting bias, Imprecision |
| Lonapegsomatropin 0.24 mg/kg/wk:YPEG-rhGH 0.14 mg/kg/wk | No concerns | Some concerns | No concerns | No concerns | No concerns | No concerns | Moderate | Reporting bias |
| Somapacitan 0.04 mg/kg/wk:Somapacitan 0.08 mg/kg/wk | Major concerns | Some concerns | Some concerns | Major concerns | No concerns | No concerns | Very low | Within-study bias, Reporting bias, Indirectness, Imprecision |
| Somapacitan 0.04 mg/kg/wk:Somapacitan 0.16 mg/kg/wk | Major concerns | Some concerns | Some concerns | Major concerns | No concerns | No concerns | Very low | Within-study bias, Reporting bias, Indirectness, Imprecision |
| Lonapegsomatropin 0.24 mg/kg/wk:Somapacitan 0.04 mg/kg/wk | Some concerns | Some concerns | No concerns | Major concerns | No concerns | No concerns | Low | Within-study bias, Reporting bias, Imprecision |
| Somapacitan 0.08 mg/kg/wk:Somapacitan 0.16 mg/kg/wk | Major concerns | Some concerns | Some concerns | Major concerns | No concerns | No concerns | Very low | Within-study bias, Reporting bias, Indirectness, Imprecision |
| Lonapegsomatropin 0.24 mg/kg/wk:Somapacitan 0.08 mg/kg/wk | Some concerns | Some concerns | No concerns | Major concerns | No concerns | No concerns | Low | Within-study bias, Reporting bias, Imprecision |
| Lonapegsomatropin 0.24 mg/kg/wk:Somapacitan 0.16 mg/kg/wk | Some concerns | Some concerns | No concerns | Some concerns | No concerns | No concerns | Low | Within-study bias, Reporting bias, Imprecision |

S8.14 Certainty of evidence for FPG

| Comparison | Within_Study_Bias | Reporting_Bias | Indirectness | Imprecision | Heterogeneity | Incoherence | Overall_Confidence | Reason_for_downgrade |
| --- | --- | --- | --- | --- | --- | --- | --- | --- |
| Daily rhGH:PEG-rhGH 0.2 mg/kg/wk | No concerns | Some concerns | No concerns | No concerns | Some concerns | No concerns | Moderate | Reporting bias, Heterogeneity |
| Daily rhGH:TransCon GH 0.14 mg/kg/wk | No concerns | Some concerns | Some concerns | Major concerns | Some concerns | No concerns | Low | Reporting bias, Indirectness, Imprecision, Heterogeneity |
| Daily rhGH:TransCon GH 0.21 mg/kg/wk | No concerns | Some concerns | Some concerns | Major concerns | Some concerns | No concerns | Low | Reporting bias, Indirectness, Imprecision, Heterogeneity |
| Daily rhGH:TransCon GH 0.30 mg/kg/wk | No concerns | Some concerns | Some concerns | Major concerns | Some concerns | No concerns | Low | Reporting bias, Indirectness, Imprecision, Heterogeneity |
| Daily rhGH:Lonapegsomatropin 0.24 mg/kg/wk | No concerns | Some concerns | No concerns | Some concerns | Some concerns | No concerns | Low | Reporting bias, Imprecision, Heterogeneity |
| PEG-rhGH 0.2 mg/kg/wk:TransCon GH 0.14 mg/kg/wk | No concerns | Some concerns | No concerns | Major concerns | Some concerns | No concerns | Low | Reporting bias, Imprecision, Heterogeneity |
| PEG-rhGH 0.2 mg/kg/wk:TransCon GH 0.21 mg/kg/wk | No concerns | Some concerns | No concerns | Major concerns | Some concerns | No concerns | Low | Reporting bias, Imprecision, Heterogeneity |
| PEG-rhGH 0.2 mg/kg/wk:TransCon GH 0.30 mg/kg/wk | No concerns | Some concerns | No concerns | Major concerns | Some concerns | No concerns | Low | Reporting bias, Imprecision, Heterogeneity |
| Lonapegsomatropin 0.24 mg/kg/wk:PEG-rhGH 0.2 mg/kg/wk | No concerns | Some concerns | No concerns | Major concerns | Some concerns | No concerns | Low | Reporting bias, Imprecision, Heterogeneity |
| TransCon GH 0.14 mg/kg/wk:TransCon GH 0.21 mg/kg/wk | No concerns | Some concerns | Some concerns | Major concerns | Some concerns | No concerns | Low | Reporting bias, Indirectness, Imprecision, Heterogeneity |
| TransCon GH 0.14 mg/kg/wk:TransCon GH 0.30 mg/kg/wk | No concerns | Some concerns | Some concerns | Major concerns | Some concerns | No concerns | Low | Reporting bias, Indirectness, Imprecision, Heterogeneity |
| Lonapegsomatropin 0.24 mg/kg/wk:TransCon GH 0.14 mg/kg/wk | No concerns | Some concerns | No concerns | Some concerns | Some concerns | No concerns | Low | Reporting bias, Imprecision, Heterogeneity |
| TransCon GH 0.21 mg/kg/wk:TransCon GH 0.30 mg/kg/wk | No concerns | Some concerns | Some concerns | Major concerns | Some concerns | No concerns | Low | Reporting bias, Indirectness, Imprecision, Heterogeneity |
| Lonapegsomatropin 0.24 mg/kg/wk:TransCon GH 0.21 mg/kg/wk | No concerns | Some concerns | No concerns | Some concerns | Some concerns | No concerns | Low | Reporting bias, Imprecision, Heterogeneity |
| Lonapegsomatropin 0.24 mg/kg/wk:TransCon GH 0.30 mg/kg/wk | No concerns | Some concerns | No concerns | Major concerns | Some concerns | No concerns | Low | Reporting bias, Imprecision, Heterogeneity |

**Supplement 9: Network Geometry Summary**

S9.1 Network Geometry Summary for Height Velocity


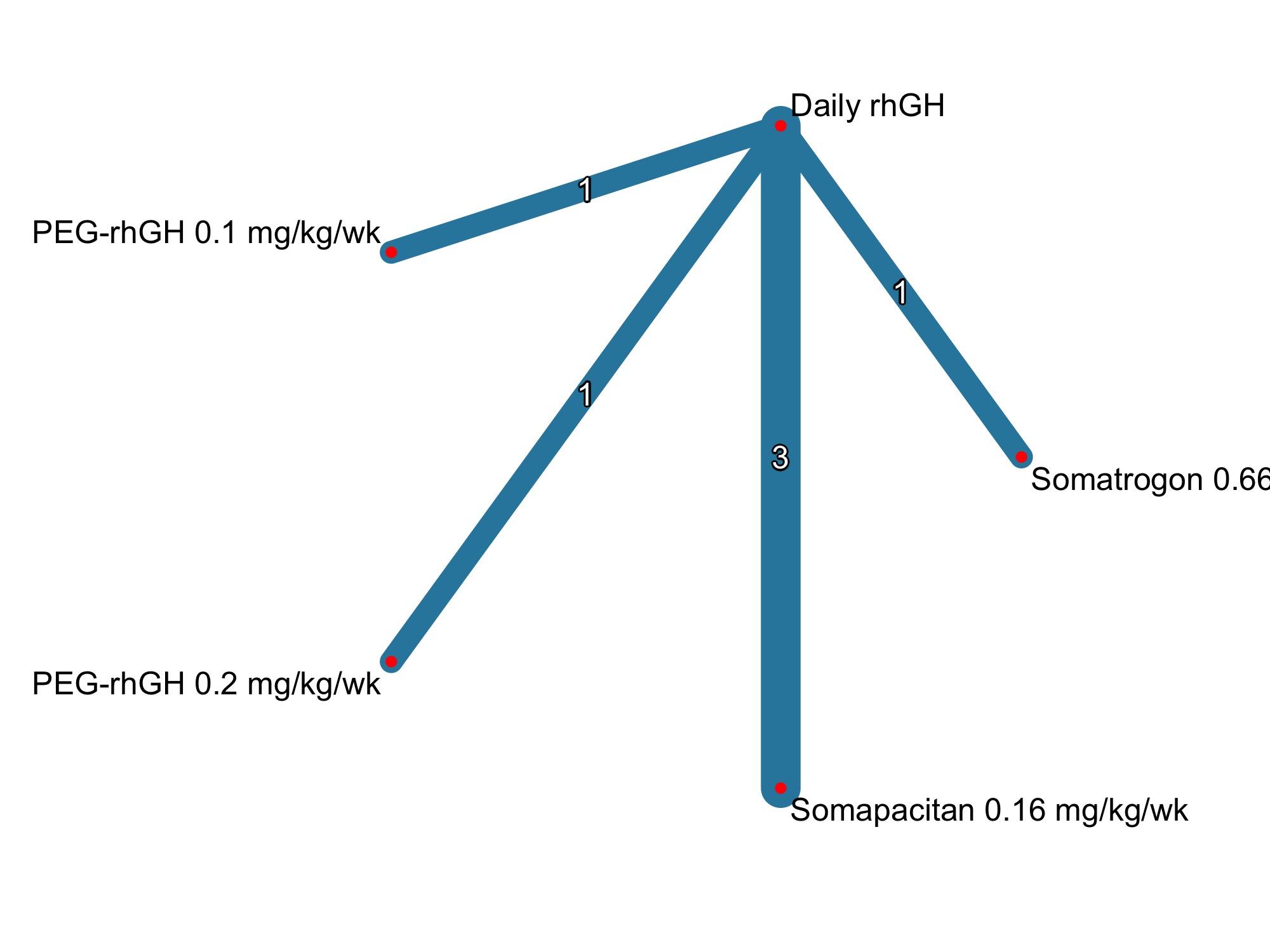


S9.2 Network Geometry Summary for Height SDS


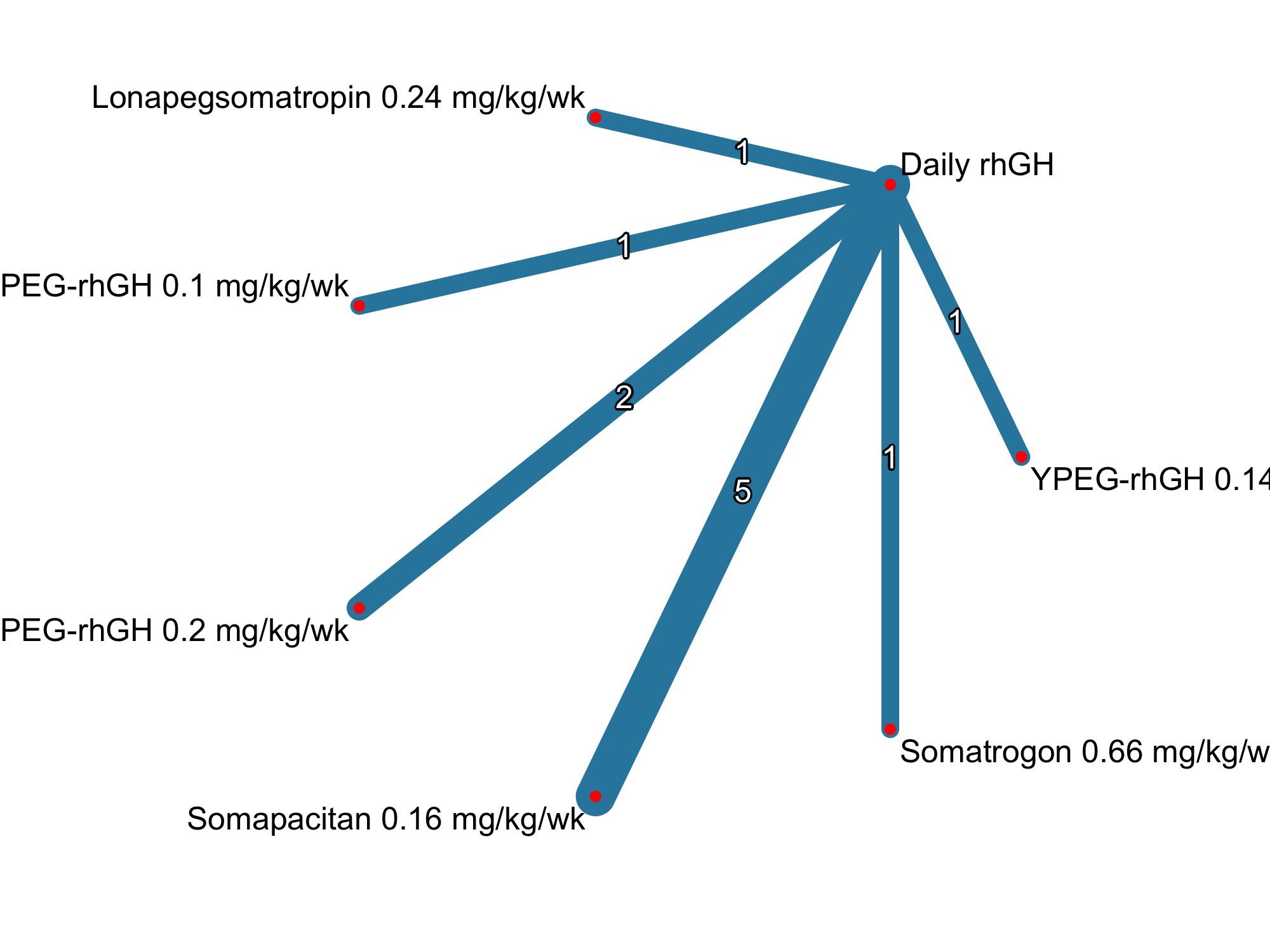


S9.3 Network Geometry Summary for Treatment Discontinuation


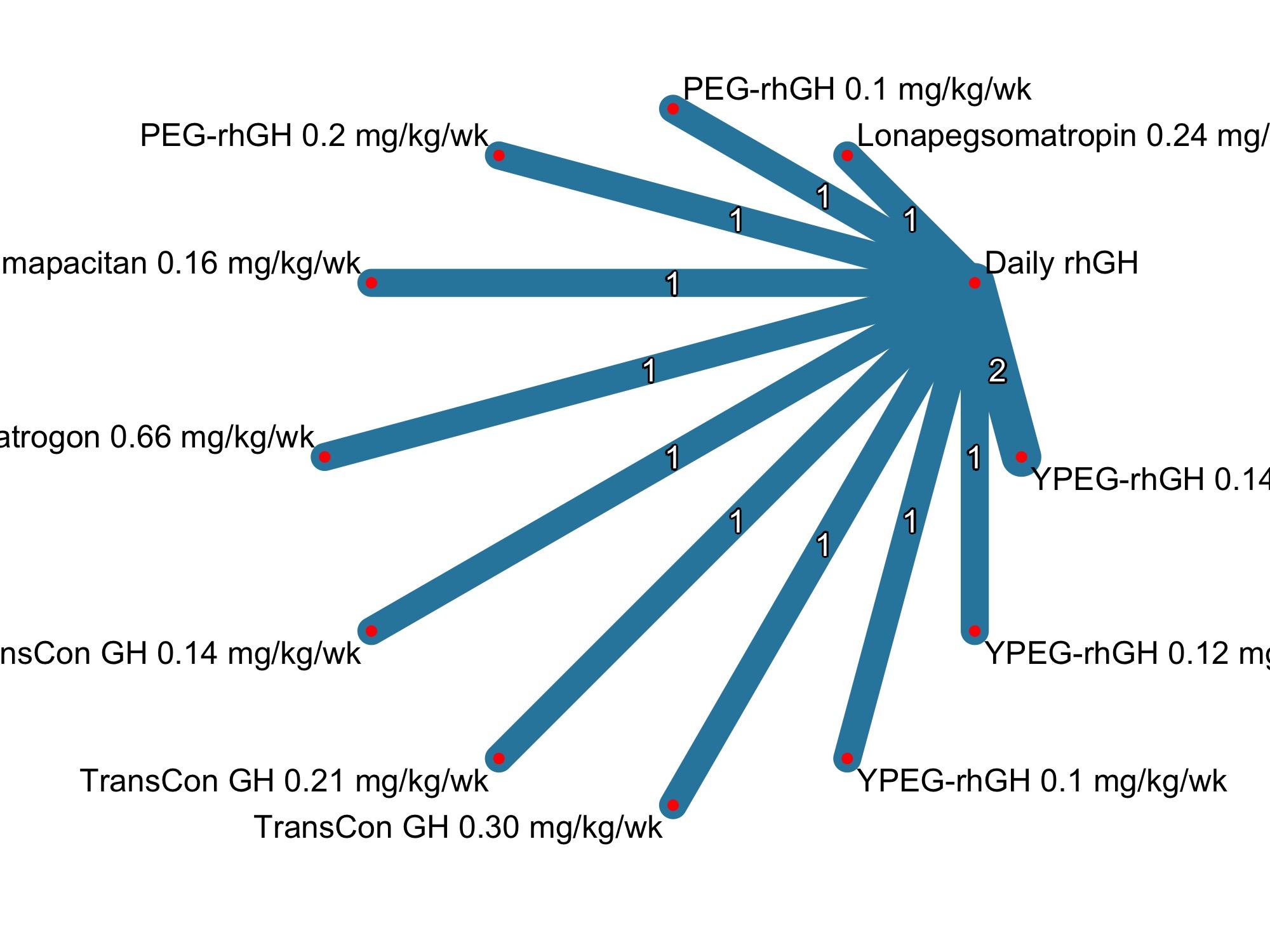


S9.4 Network Geometry Summary for Injection Site Erythema


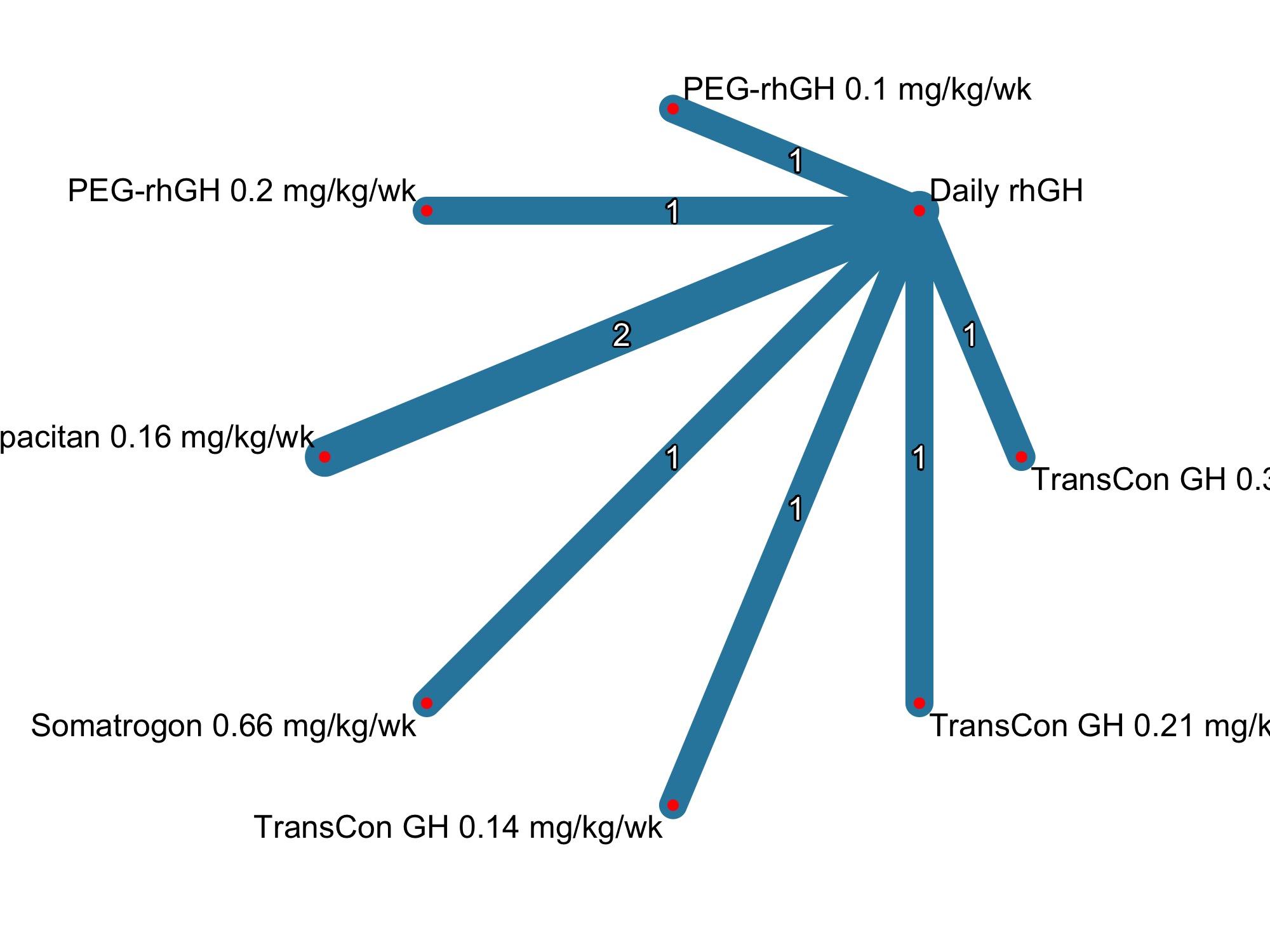


S9.5 Network Geometry Summary for Influenza


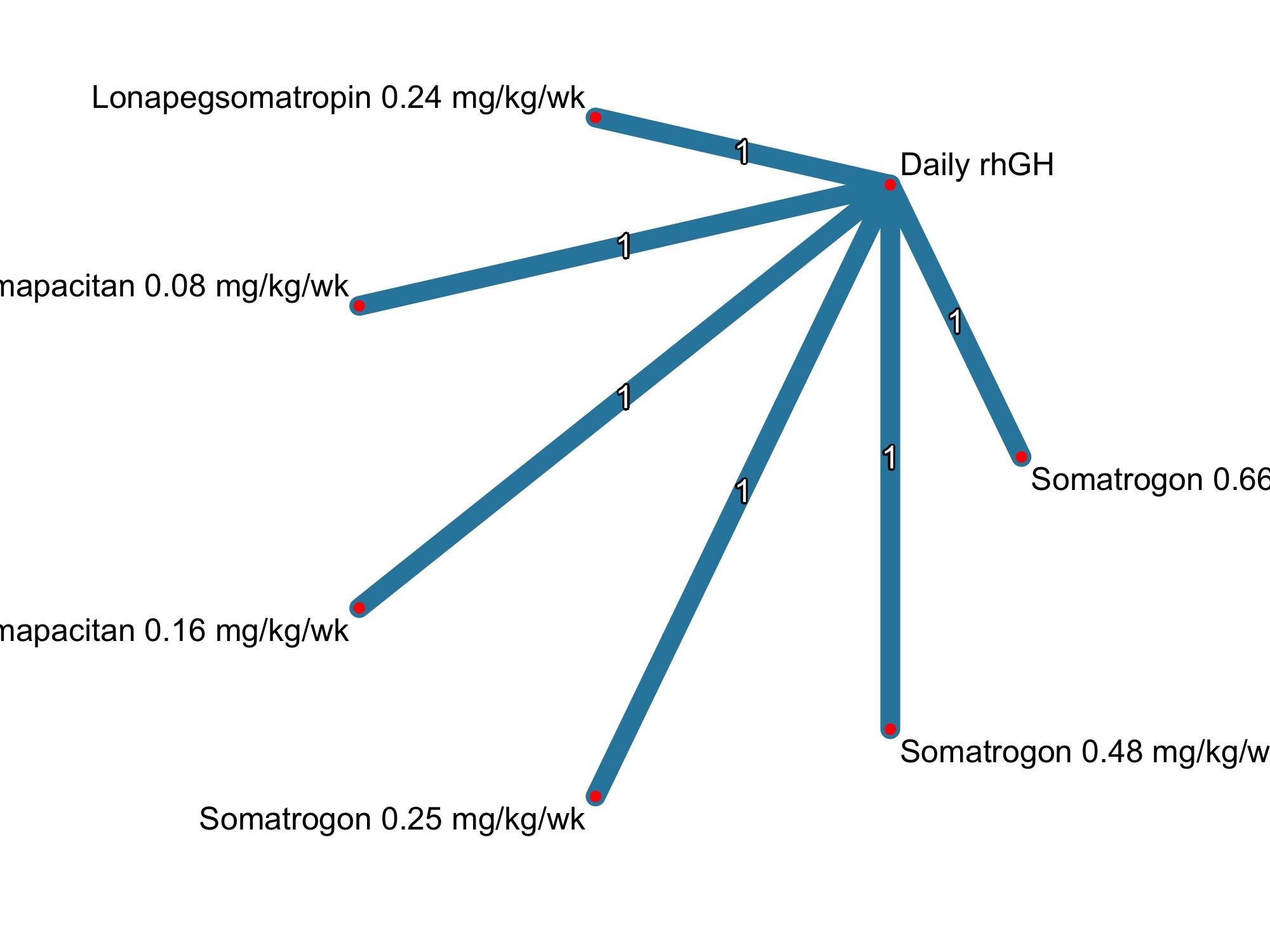


S9.6 Network Geometry Summary for Headache


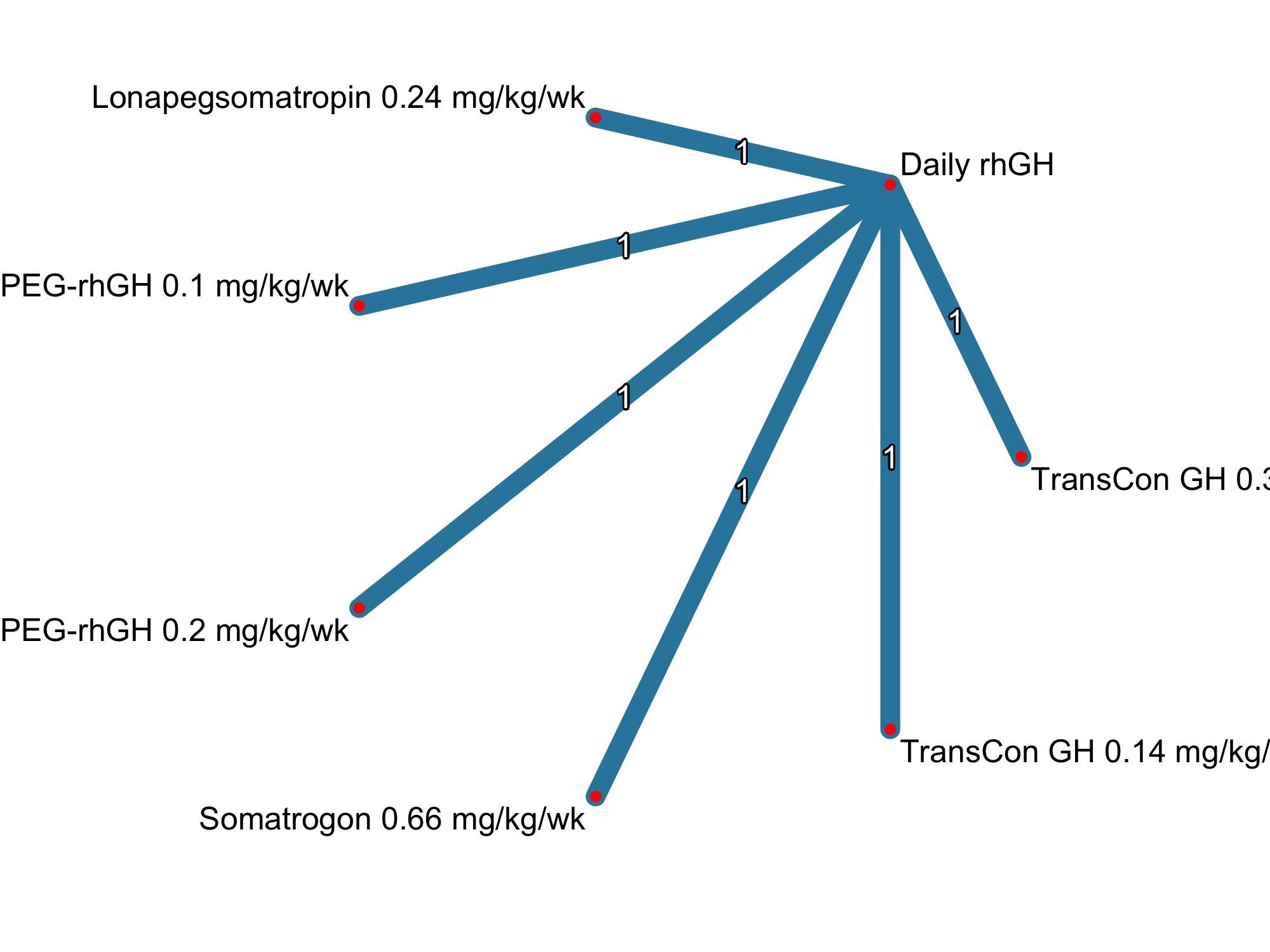


S9.7 Network Geometry Summary for Fever


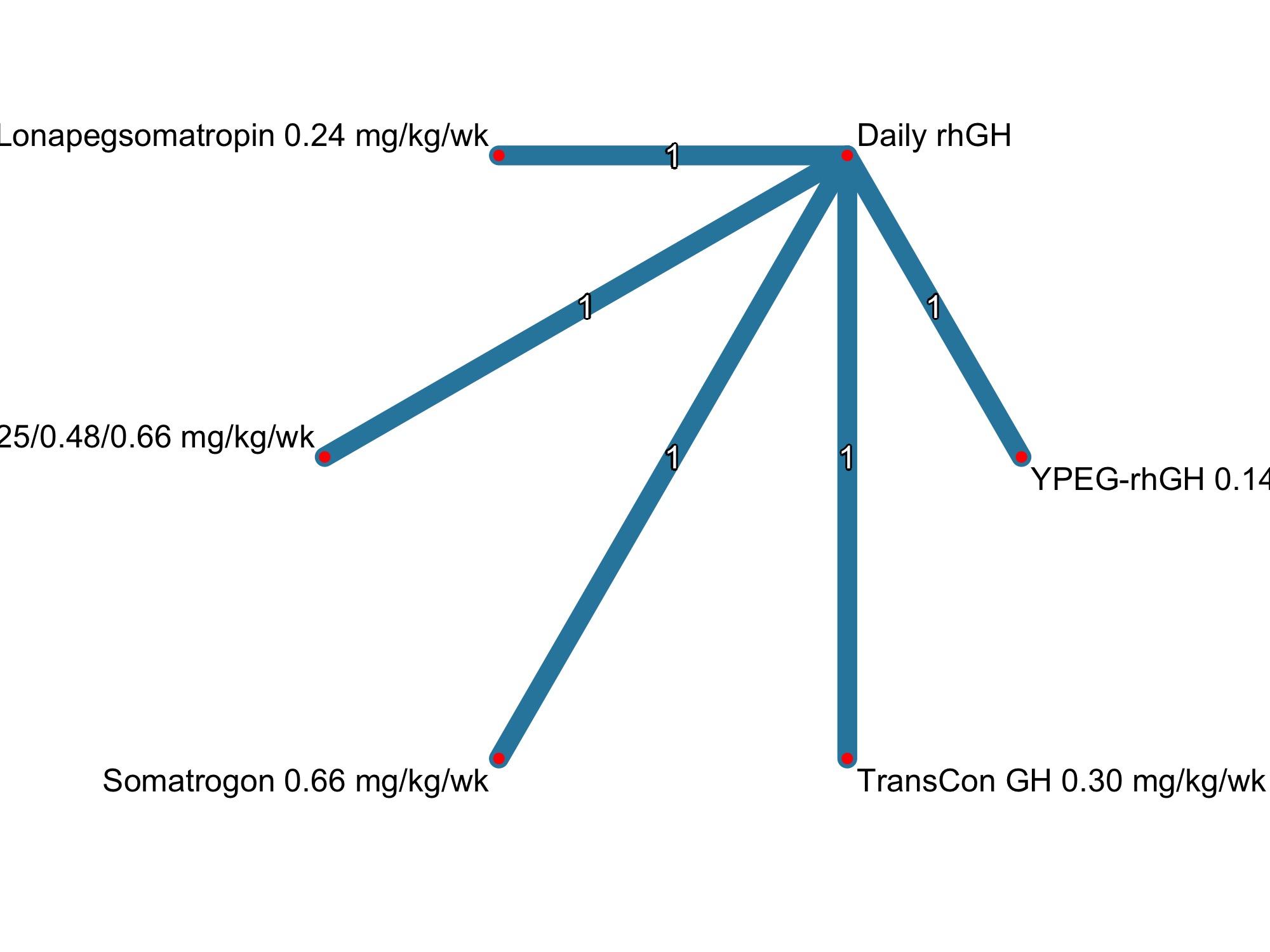


S9.8 Network Geometry Summary for hypothyroidism


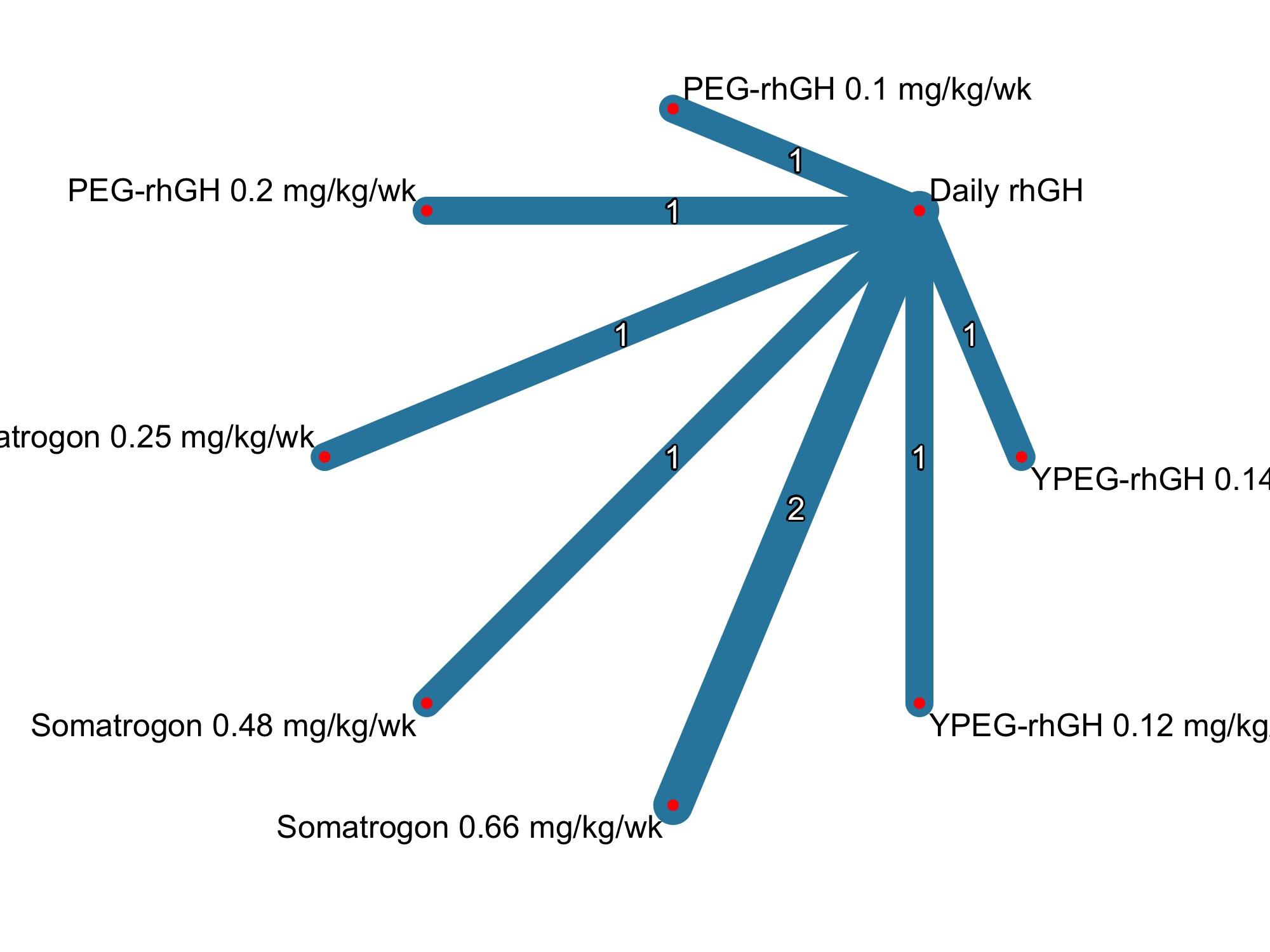


S9.9 Network Geometry Summary for Injection Site Pain


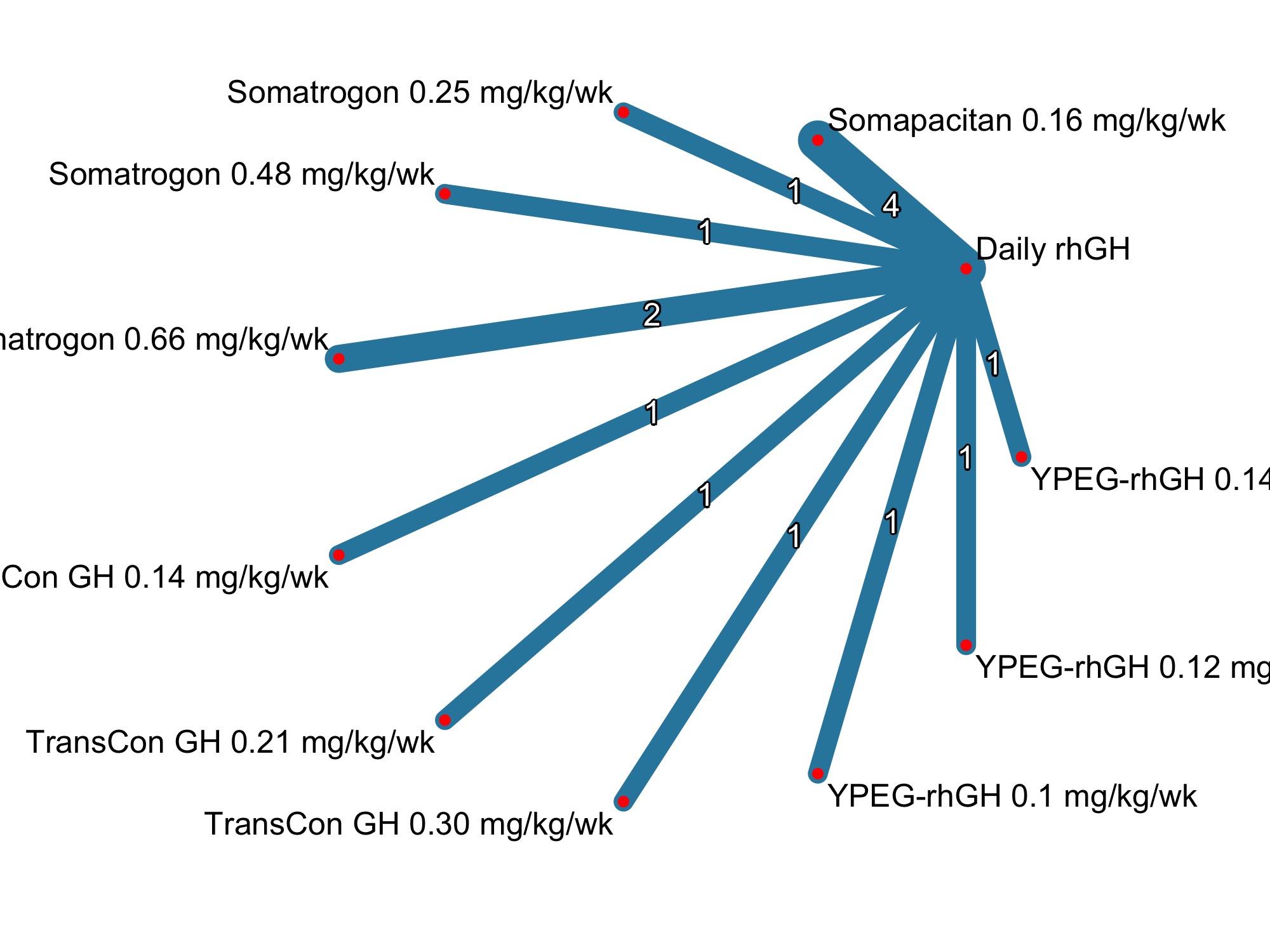


S9.10 Network Geometry Summary for IGF-1


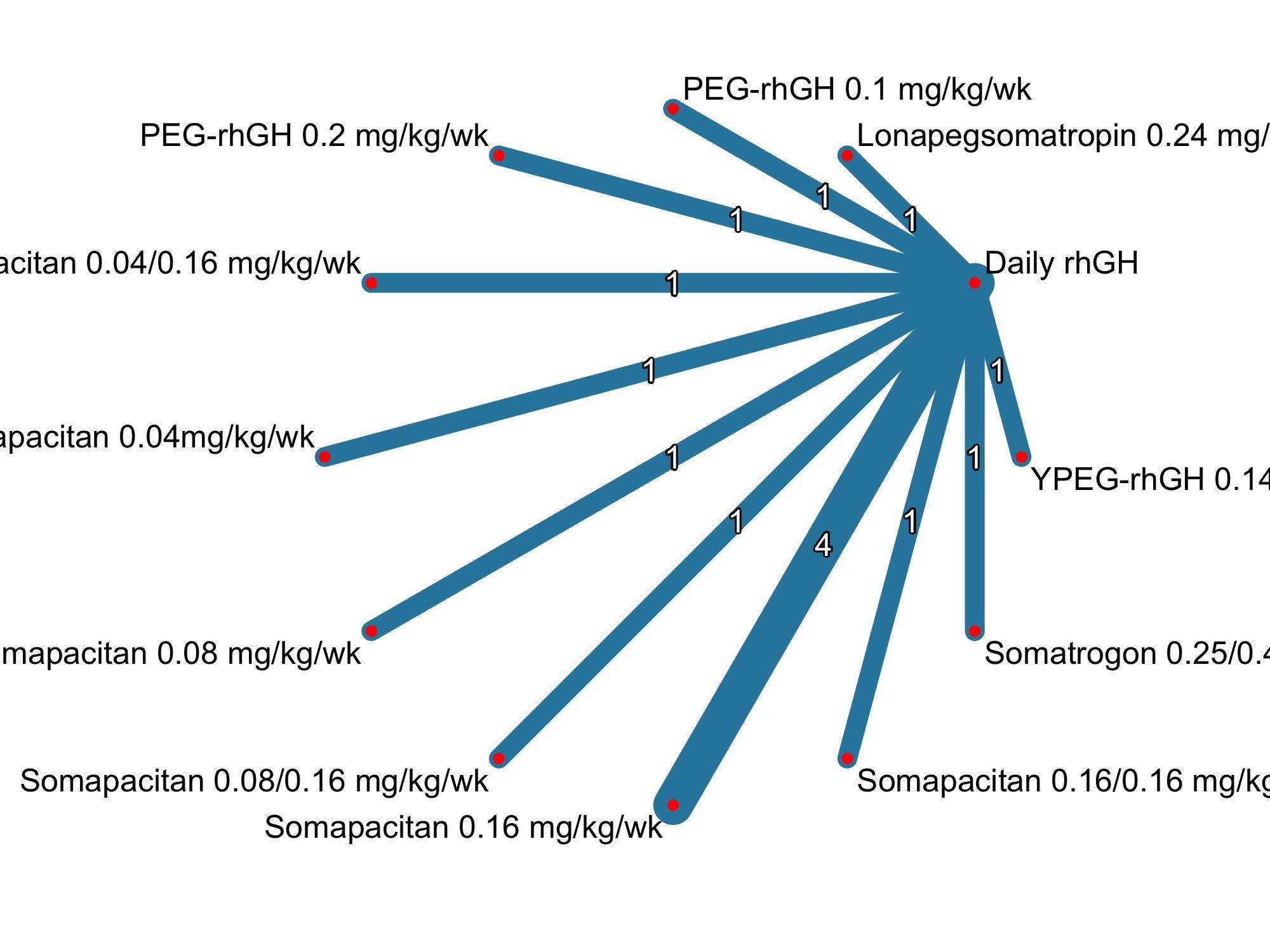


S9.11 Network Geometry Summary for HbA1C%


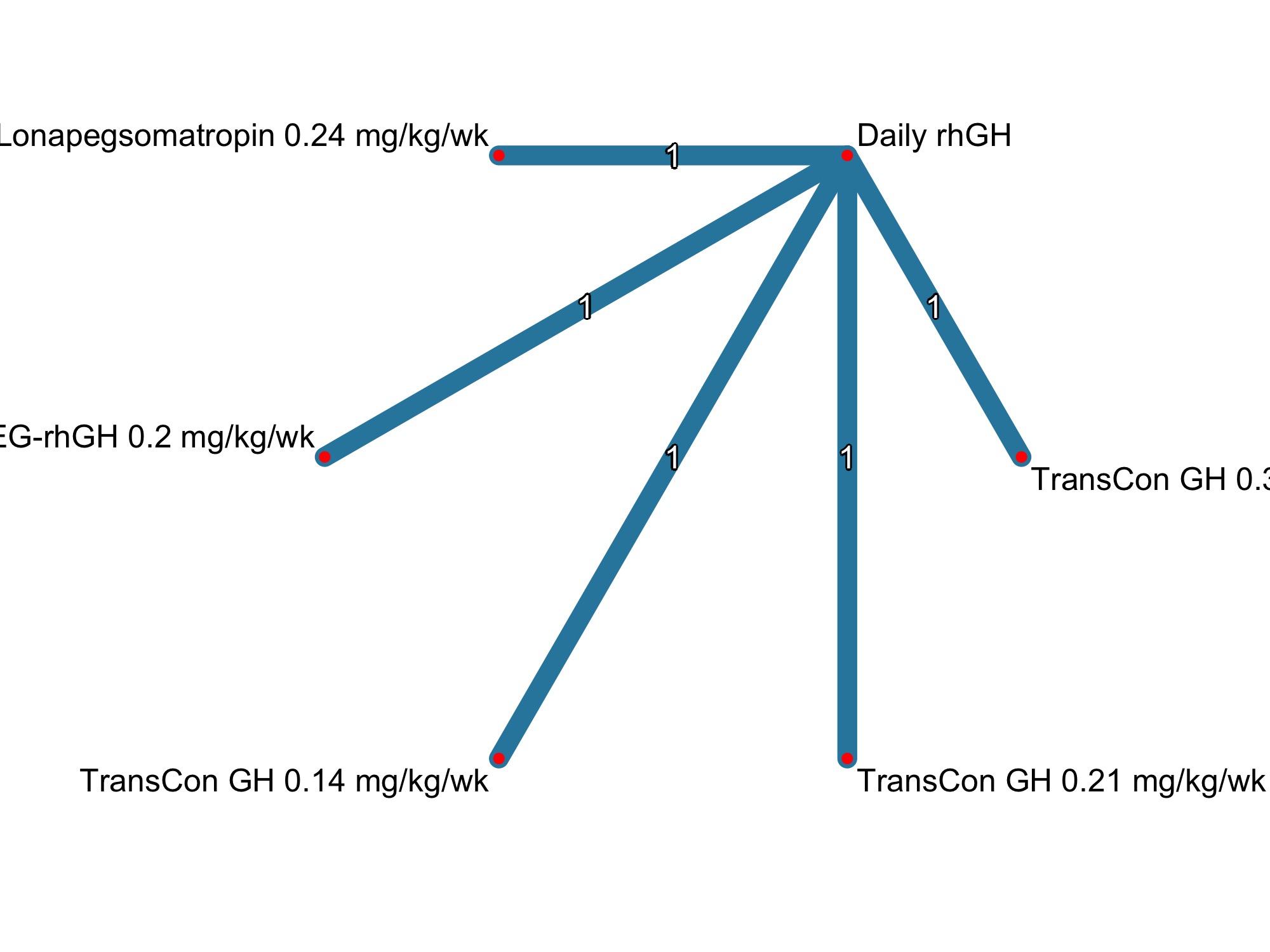


S9.12 Network Geometry Summary for cough


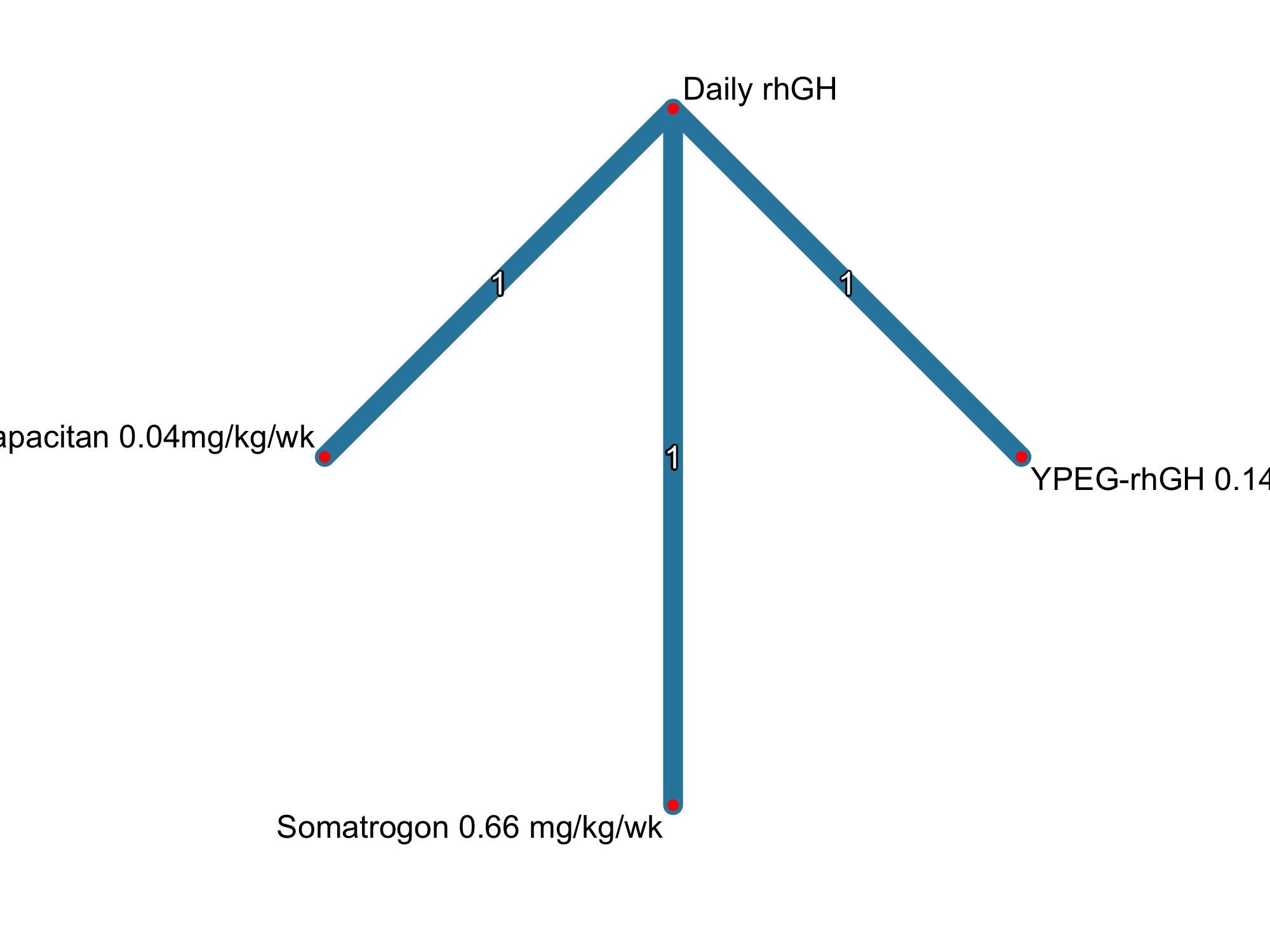


S9.13 Network Geometry Summary for URTI


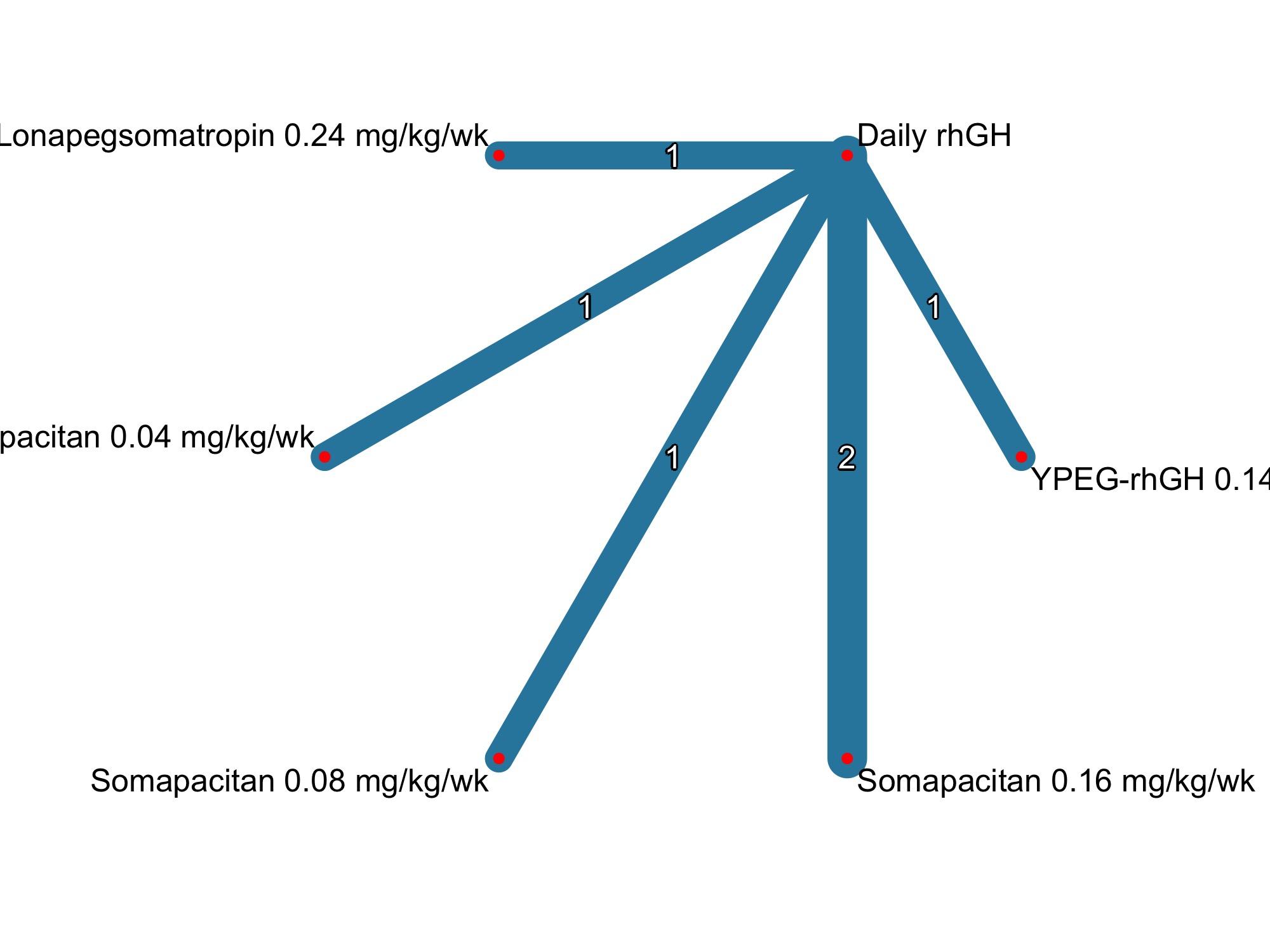


S9.14 Network Geometry Summary for FPG


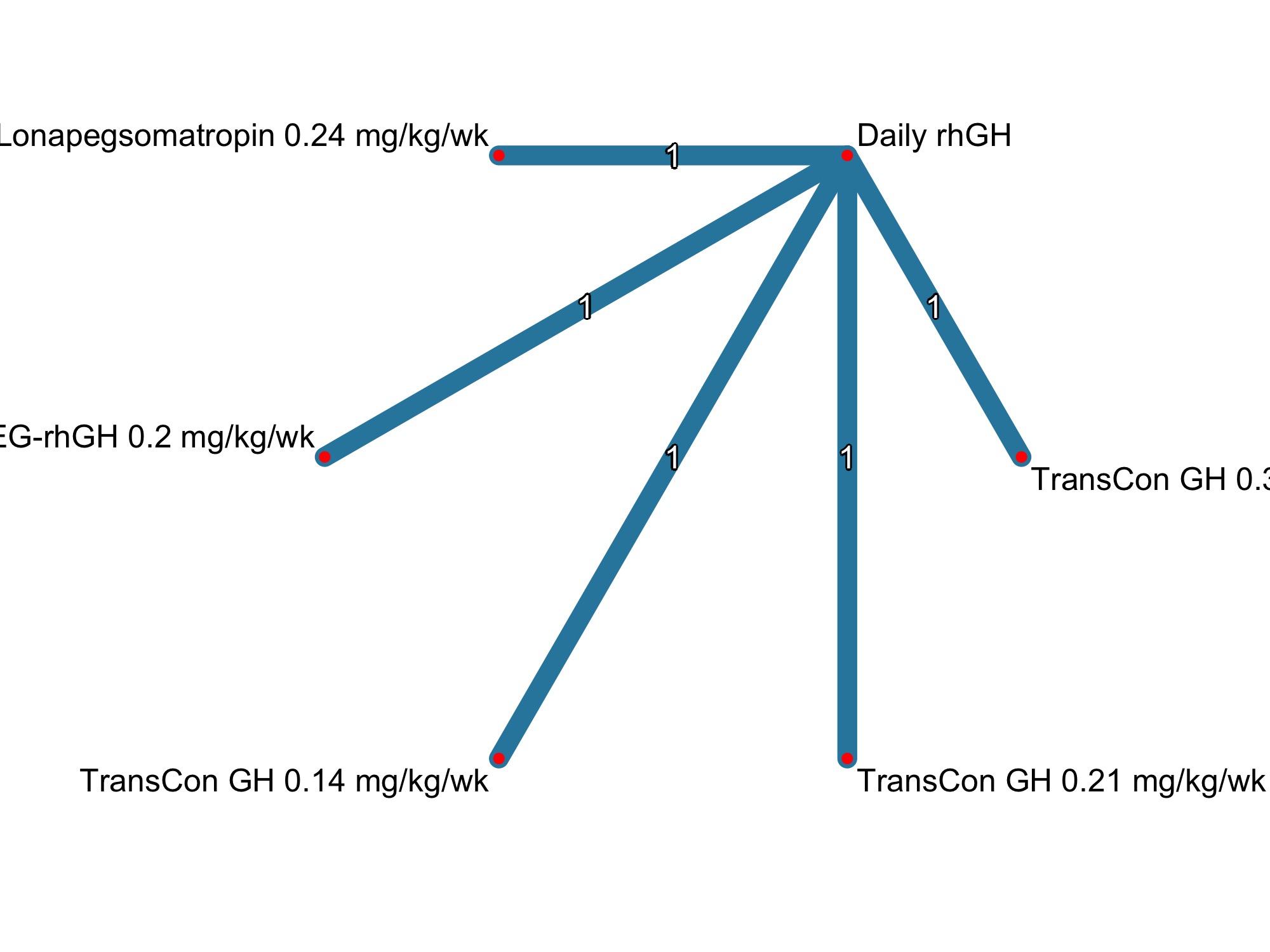


**Supplement 10: PRISMA Checklist**

| **Section and Topic** | **Item #** | **Checklist item** | **Location where item is reported** |
| --- | --- | --- | --- |
| **TITLE** | | | 1 |
| Title | 1 | Identify the report as a systematic review. |  |
| **ABSTRACT** | | | 1 |
| Abstract | 2 | See the PRISMA 2020 for Abstracts checklist. |  |
| **INTRODUCTION** | | | 2 |
| Rationale | 3 | Describe the rationale for the review in the context of existing knowledge. | 2 |
| Objectives | 4 | Provide an explicit statement of the objective(s) or question(s) the review addresses. | 3 |
| **METHODS** | | |  |
| Eligibility criteria | 5 | Specify the inclusion and exclusion criteria for the review and how studies were grouped for the syntheses. | 3 |
| Information sources | 6 | Specify all databases, registers, websites, organisations, reference lists and other sources searched or consulted to identify studies. Specify the date when each source was last searched or consulted. | 4 |
| Search strategy | 7 | Present the full search strategies for all databases, registers and websites, including any filters and limits used. | S1 |
| Selection process | 8 | Specify the methods used to decide whether a study met the inclusion criteria of the review, including how many reviewers screened each record and each report retrieved, whether they worked independently, and if applicable, details of automation tools used in the process. | 4 |
| Data collection process | 9 | Specify the methods used to collect data from reports, including how many reviewers collected data from each report, whether they worked independently, any processes for obtaining or confirming data from study investigators, and if applicable, details of automation tools used in the process. | 4 |
| Data items | 10a | List and define all outcomes for which data were sought. Specify whether all results that were compatible with each outcome domain in each study were sought (e.g. for all measures, time points, analyses), and if not, the methods used to decide which results to collect. |  |
|  | 10b | List and define all other variables for which data were sought (e.g. participant and intervention characteristics, funding sources). Describe any assumptions made about any missing or unclear information. |  |
| Study risk of bias assessment | 11 | Specify the methods used to assess risk of bias in the included studies, including details of the tool(s) used, how many reviewers assessed each study and whether they worked independently, and if applicable, details of automation tools used in the process. | 4 |
| Effect measures | 12 | Specify for each outcome the effect measure(s) (e.g. risk ratio, mean difference) used in the synthesis or presentation of results. |  |
| Synthesis methods | 13a | Describe the processes used to decide which studies were eligible for each synthesis (e.g. tabulating the study intervention characteristics and comparing against the planned groups for each synthesis (item #5)). | 4 |
|  | 13b | Describe any methods required to prepare the data for presentation or synthesis, such as handling of missing summary statistics, or data conversions. | 4 |
|  | 13c | Describe any methods used to tabulate or visually display results of individual studies and syntheses. | 4 |
|  | 13d | Describe any methods used to synthesize results and provide a rationale for the choice(s). If meta-analysis was performed, describe the model(s), method(s) to identify the presence and extent of statistical heterogeneity, and software package(s) used. | 4 |
|  | 13e | Describe any methods used to explore possible causes of heterogeneity among study results (e.g. subgroup analysis, meta-regression). | 4 |
|  | 13f | Describe any sensitivity analyses conducted to assess robustness of the synthesized results. | 4 |
| Reporting bias assessment | 14 | Describe any methods used to assess risk of bias due to missing results in a synthesis (arising from reporting biases). | 4 |
| Certainty assessment | 15 | Describe any methods used to assess certainty (or confidence) in the body of evidence for an outcome. | 4 |
| **RESULTS** | | |  |
| Study selection | 16a | Describe the results of the search and selection process, from the number of records identified in the search to the number of studies included in the review, ideally using a flow diagram. | 5 |
|  | 16b | Cite studies that might appear to meet the inclusion criteria, but which were excluded, and explain why they were excluded. | 5 |
| Study characteristics | 17 | Cite each included study and present its characteristics. | S2, S3 |
| Risk of bias in studies | 18 | Present assessments of risk of bias for each included study. | S4 |
| Results of individual studies | 19 | For all outcomes, present, for each study: (a) summary statistics for each group (where appropriate) and (b) an effect estimate and its precision (e.g. confidence/credible interval), ideally using structured tables or plots. | 5 |
| Results of syntheses | 20a | For each synthesis, briefly summarise the characteristics and risk of bias among contributing studies. | 5-10 |
|  | 20b | Present results of all statistical syntheses conducted. If meta-analysis was done, present for each the summary estimate and its precision (e.g. confidence/credible interval) and measures of statistical heterogeneity. If comparing groups, describe the direction of the effect. | 5-10 |
|  | 20c | Present results of all investigations of possible causes of heterogeneity among study results. | 5-10 |
|  | 20d | Present results of all sensitivity analyses conducted to assess the robustness of the synthesized results. | 5-10 |
| Reporting biases | 21 | Present assessments of risk of bias due to missing results (arising from reporting biases) for each synthesis assessed. | 5-10 |
| Certainty of evidence | 22 | Present assessments of certainty (or confidence) in the body of evidence for each outcome assessed. | 5-10 |
| **DISCUSSION** | | |  |
| Discussion | 23a | Provide a general interpretation of the results in the context of other evidence. | 10-13 |
|  | 23b | Discuss any limitations of the evidence included in the review. | 14 |
|  | 23c | Discuss any limitations of the review processes used. | 14 |
|  | 23d | Discuss implications of the results for practice, policy, and future research. | 15 |
| **OTHER INFORMATION** | | |  |
| Registration and protocol | 24a | Provide registration information for the review, including register name and registration number, or state that the review was not registered. | 3 |
|  | 24b | Indicate where the review protocol can be accessed, or state that a protocol was not prepared. | 3 |
|  | 24c | Describe and explain any amendments to information provided at registration or in the protocol. | - |
| Support | 25 | Describe sources of financial or non-financial support for the review, and the role of the funders or sponsors in the review. | 1 |
| Competing interests | 26 | Declare any competing interests of review authors. | 1 |
| Availability of data, code and other materials | 27 | Report which of the following are publicly available and where they can be found: template data collection forms; data extracted from included studies; data used for all analyses; analytic code; any other materials used in the review. | 1 |

*From:*  Page MJ, McKenzie JE, Bossuyt PM, Boutron I, Hoffmann TC, Mulrow CD, et al. The PRISMA 2020 statement: an updated guideline for reporting systematic reviews. BMJ 2021;372:n71. doi: 10.1136/bmj.n71. This work is licensed under CC BY 4.0. To view a copy of this license, visit <https://creativecommons.org/licenses/by/4.0/>
